# Evolution of resource specialisation in competitive metacommunities

**DOI:** 10.1111/ele.13338

**Published:** 2019-08-07

**Authors:** Jonas Wickman, Sebastian Diehl, Åke Brännström

**Affiliations:** ^1^ Integrated Science Lab, Department of Mathematics and Mathematical Statistics Umeå University SE‐90187 Umeå Sweden; ^2^ Integrated Science Lab, Department of Ecology and Environmental Science Umeå University SE‐90187 Umeå Sweden; ^3^ Evolution and Ecology Program International Institute for Applied Systems Analysis (IIASA) Schlossplatz 12361 Laxenburg Austria

**Keywords:** Adaptive dynamics, coexistence, consumer–resource interactions, ESS, spatial models

## Abstract

Spatial environmental heterogeneity coupled with dispersal can promote ecological persistence of diverse metacommunities. Does this premise hold when metacommunities evolve? Using a two‐resource competition model, we studied the evolution of resource‐uptake specialisation as a function of resource type (substitutable to essential) and shape of the trade‐off between resource uptake affinities (generalist‐ to specialist‐favouring). In spatially homogeneous environments, evolutionarily stable coexistence of consumers is only possible for sufficiently substitutable resources and specialist‐favouring trade‐offs. Remarkably, these same conditions yield comparatively low diversity in heterogeneous environments, because they promote sympatric evolution of two opposite resource specialists that, together, monopolise the two resources everywhere. Consumer diversity is instead maximised for intermediate trade‐offs and clearly substitutable or clearly essential resources, where evolved metacommunities are characterised by contrasting selection regimes. Taken together, our results present new insights into resource‐competition‐mediated evolutionarily stable diversity in homogeneous and heterogeneous environments, which should be applicable to a wide range of systems.

## Introduction

A fundamental and enigmatic question in ecology and evolution is as follows: Why are there so many species (Hutchinson 1959)? From a resource competition perspective, high biodiversity is unexpected, because membership in guilds of competitors is limited by the number of unique resources (Hardin 1960; Levin 1970; Gyllenberg & Meszéna 2005; Meszéna *et al.* 2006). The formation and maintenance of diversity is therefore believed to depend on temporal and spatial variability. In particular, many ecological concepts of biodiversity invoke spatial heterogeneity and resulting source–sink dynamics as driven by disturbance–competition trade‐offs (Connell 1978), colonisation–competition trade‐offs (Tilman 1994), or regional species sorting (Tilman 1982; Amarasekare & Nisbet 2001; Mouquet & Loreau 2003). But are these ecological scenarios of high diversity also evolutionarily plausible? This is by no means obvious, because eco‐evolutionary models commonly predict that the number of distinct phenotypes at an evolutionary endpoint is considerably lower than the number of phenotypes that could coexist ecologically (Koffel *et al.* 2016; Edwards *et al.* 2018).

A recent hypothesis posits that spatial heterogeneity in the supply ratio of as few as two limiting resources can sustain an unlimited regional diversity of competing consumers over evolutionary time scales, if consumers exploit the two resources differently but are constrained to a universal interspecific trade‐off in their utilisation (Tilman 2011). Strictly, this can only hold in the limit of no dispersal and perfect adaptation to the differences in local resource supply ratios (Abrams 1988). Once dispersal is acknowledged, a challenging question arises: Under which circumstances will the antagonistic interplay of spatially variable local selection and homogenising dispersal engender regional diversification of resource competitors? In this paper, we explore this question with a focus on trade‐offs in resource–acquisition traits and the degree of resource substitutability.

Limiting resources are, by definition, scarce. The ability to extract scarce resources from the environment therefore conveys a competitive advantage and will be under natural selection. This trait, conventionally termed affinity (Button 1986), is a fundamental component of all functions describing resource uptake in models of consumer–resource interactions. For example, in predator–prey and plant–herbivore models, affinity takes on the form of a search rate (Holling 1959; Real 1977), and models of substrate uptake by microorganisms use an equivalent, inverse form called the half‐saturation constant (Grover *et al.* 1997; Litchman & Klausmeier 2008). A high affinity conveys a competitive advantage, because a consumer's ability to subsist on a scarce resource is proportional to its affinity, and the outcome of competition is dictated by the lowest resource density a species can tolerate (Tilman 1982). Typically, an organism’s ability to increase uptake of one resource comes at the expense of reduced uptake of some other resource (e.g. Edwards *et al.* 2011). The resulting trade‐off in affinities will constrain the evolution of resource uptake. Trade‐offs can be either specialist‐favouring – for which total resource uptake is the greatest when uptake is biased towards the preferred resource – or generalist‐favouring, for which total uptake is greatest when resource uptake is balanced.

To date, the evolution of uptake specialisation has been primarily studied in models of spatially homogeneous systems, where coexistence between consumers differing in resource affinities was found to be possible for specialist‐favouring trade‐offs (Levins 1962; MacArthur & Levins 1964; Lawlor & Smith 1976; Rueffler *et al.* 2006). These studies only considered perfectly substitutable resources, and it remains unclear whether similar patterns would arise when resources are essential or antagonistic. Conversely, the only study that included different resources types (antagonistic to substitutable to essential, Schreiber & Tobiason 2003) considered, in turn, only linear affinity trade‐offs, which are neither specialist nor generalist‐favouring. Finally, the few studies considering heterogeneous environments focused, again, on perfectly substitutable resources (Parvinen & Egas 2004; Nurmi & Parvinen 2008; Débarre & Gandon 2010; Wickman *et al.* 2017). Yet, resource types are rarely perfectly substitutable, and trade‐offs are rarely perfectly linear, and their joint effects on trait evolution and coexistence of resource competitors are poorly understood. A systematic investigation of these interactions is needed if we wish to understand and predict the evolution of metacommunities of resource competitors.

In this study, we investigate the evolution of resource‐uptake specialisation for different types of resources that range continuously from substitutable to essential, and for trade‐offs in resource affinity that range continuously from generalist‐ to specialist‐favouring. We explore an evolutionary model of competition for two resources and identify combinations of resource types and trade‐offs that promote evolutionarily stable coexistence of competiting consumers. We first analytically characterise the combinations of resource types and trade‐offs that permit evolutionarily stable coexistence of two consumers in spatially homogeneous environments. This enables us to subsequently explain why consumer diversity in evolutionarily stable metacommunities is predicted to be high for some combinations of resource types and affinity trade‐offs, but can be extremely low for others, given otherwise identical landscapes of spatially variable resource supply ratios.

## Models and Methods

To investigate how different resource types and affinity trade‐offs affect evolutionary community assembly, we explore differential equation models describing how consumers compete for two resources. We begin our investigations in homogeneous environments where all variables are constant in space, and subsequently extend the model to heterogeneous environments where resource supply rates vary across the landscape.

### Homogeneous environments

We let *N* consumers with densities *u_i_*,* i* = 1, 2, …, *N*, compete for two resources with densities *R*_1_ and *R*_2_. Each consumer takes up resources 1 and 2 with affinities *a_i_*_1_ and *a_i_*_2_, respectively, where affinity is defined as a constant that enters multiplicatively with resource density into the consumers’ uptake and growth rates. We let these affinities be under selection within the constraints of a trade‐off. The ecological dynamics are described by the following:

(1a) $$\frac{du_{i}\left(t\right)}{dt}=G\left(a_{i1}R_{1},a_{i2}R_{2}\right)u_{i}-\mu u_{i},$$

(1b) $$\frac{dR_{1}\left(t\right)}{dt}=r\left(K-R_{1}\right)-\sum_{i=1}^{N}C_{1}\left(a_{i1}R_{1},\;a_{i2}R_{2}\right)G\left(a_{i1}R_{1},\;a_{i2}R_{2}\right)u_{i},$$

(1c) $$\frac{dR_{2}\left(t\right)}{dt}=r\left(K-R_{2}\right)-\sum_{i=1}^{N}C_{2}\left(a_{i1}R_{1},\;a_{i2}R_{2}\right)G\left(a_{i1}R_{1},\;a_{i2}R_{2}\right)u_{i}.$$

Here, *G* is the per capita growth function of consumer *i* depending on resource densities and affinities, and *μ* is the consumer's mortality rate. Resources are supplied with chemostat dynamics at rate *r* and supply density *K*. Each resource is consumed in proportion to its fractional contribution *C_j_* to gross consumer growth *Gu_i_*. For notational simplicity, all resource conversion efficiencies are set to 1. Thus, *C*_1_ + *C*_2_ ≡ 1 for all combinations of affinities and resource densities, and consumer growth equals the sum of removed resources. Relaxing this assumption does not affect the results when conversion efficiencies are equal for all resources.

The functions *G*,* C*_1_ and *C*_2_ depend on what we subsequently call *effective resource availabilities*, that is, the products *a_ij_R_j_* of the affinities and resource densities *j* = 1, 2. A multiplicative affinity of this type is found in all common specific resource‐uptake and growth functions, including linear and type II functional responses, and minimum and multiplicative growth functions. Our general formulation of functions *G* and *C_j_* includes all of these specific functions as special cases (Appendix S2.3 in Supporting Information). We illustrate this with an example assuming a type II functional response for substitutable resources:

(2a) $$\frac{du_{i}\left(t\right)}{dt}=\frac{a_{i1}R_{1}+a_{i2}R_{2}}{1+ha_{i1}R_{1}+ha_{i2}R_{2}}u_{i}-\mu u_{i},$$

(2b) $$\frac{dR_{1}\left(t\right)}{dt}=r\left(K-R_{1}\right)-\sum_{i=1}^{N}\frac{a_{i1}R_{1}}{1+ha_{i1}R_{1}+ha_{i2}R_{2}}u_{i},$$

(2c) $$\frac{dR_{2}\left(t\right)}{dt}=r\left(K-R_{2}\right)-\sum_{i=1}^{N}\frac{a_{i2}R_{2}}{1+ha_{i1}R_{1}+ha_{i2}R_{2}}u_{i}.$$

Here, the per capita growth function is *G*(*a_i_*_1_ *R*_1_, *a_i_*_2_ *R*_2_) = (*a_i_*_1_ *R*_1_ + *a_i_*_2_ *R*_2_)/(1 + *ha_i_*_1_ *R*_1_ + *ha_i_*_2_ *R*_2_) (assuming a conversion efficiency of 1), the proportional consumption of resource *j* is *C_j_*(*a_i_*_1_ *R*_1_, *a_i_*_2_ *R*_2_) = *a_ij_R_j_*/(*a_i_*_1_ *R*_1_ + *a_i_*_2_ *R*_2_), *h* is handling time (which is identical for all consumers and resources), and the attack rates *a_i_*_1_ and *a_i_*_2_ correspond to the affinities of the general model.

Given our focus on the evolution of specialisation in resource uptake, we let evolution act exclusively on each consumer's affinities, and assume that resource uptake only differs with respect to *a_i_*_1_ and *a_i_*_2_. This implies that supply *K*, renewal rate *r*, and any other resource‐related consumer trait (such as handling time *h*, eqn 2) are the same for both resources. The above assumptions entail that the functions describing a consumer's per capita growth and proportional consumption are both symmetrical across the diagonal in a state space of effective resource availabilities *a_ij_R_j_*. Finally, we assume that consumers consume proportionally more of the resource for which *a_ij_R_j_* is highest. Mathematically, this implies that, if *a_i_*_1_ *R*_1_ ≥ *a_i_*_2_ *R*_2_, then *C*_1_(*a_i_*_1_ *R*_1_, *a_i_*_2_ *R*_2_) ≥ *C*_2_(*a_i_*_1_ *R*_1_, *a_i_*_2_ *R*_2_).

**Figure 1:**
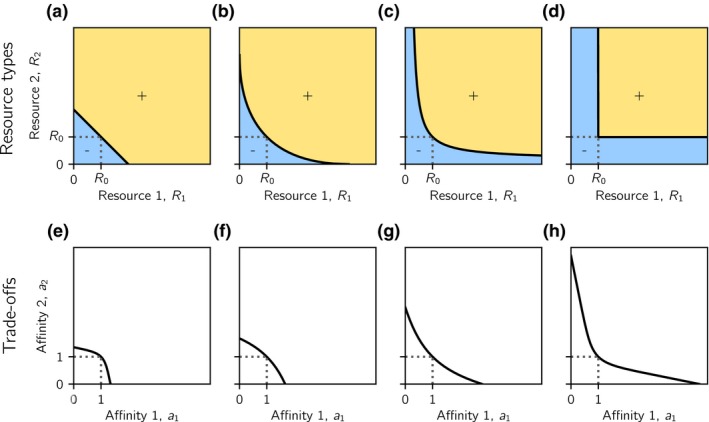
Examples of resource types and trade‐offs. Panels (a–d): Sign of the net growth rate of a generalist consumer (for which *a*_1_ = *a*_2_ = 1) as a function of the densities of resources 1 and 2 for (a) perfectly substitutable resources, (b) complementary, substitutable resources, (c) interactively essential resources and (d) strictly essential resources. Net growth is positive in the yellow region, negative in the blue region and zero on the black line, which is the generalist consumer's zero net growth isocline (generalist ZNGI). Different resource types are characterised by different generalist ZNGIs. All generalist ZNGIs go through the point (*R*_0_, *R*_0_). Panels (e–h): Trade‐off curves between the affinities *a*_1_ and *a*_2_ for the two resources. Shown are trade‐offs that (e) strongly favour resource generalists, (f) weakly favour resource generalists, (g) weakly favour resource specialists and (h) strongly favour resource specialists. All trade‐offs go through the point (1, 1). For comparison with Fig. 4 and Fig. 6, the normalised curvatures of the generalist ZNGIs, ${\hat{\kappa }}_{Z}$, in panels (a)–(d) are 0, 0.5, 1.25 and 101 respectively. The normalised curvatures of the trade‐off curves, ${\hat{\kappa }}_{T}$, in panels (e)–(h) are approximately − 2.47, − 0.50, 0.47 and 1.42 respectively.

To quantify the degree to which resources are substitutable or essential, we introduce the concept of a *generalist ZNGI*, which is the zero net growth isocline (ZNGI) of a generalist consumer, that is, a consumer that has equal affinities for both resources (*a*_1_ = *a*_2_ = 1). The generalist ZNGI is the curve in resource space that satisfies *G*(*R*_1_, *R*_2_)*u_i_* − *μu_i_* = 0, that is, any combination of resources for which the generalist consumer has zero net growth. Resources are perfectly substitutable (Fig. 1a), if a generalist consumer can substitute a given intake of one resource by a proportional intake of the other resource, and still remain in equilibrium. Resources are strictly essential (Fig. 1d), if a generalist consumer requires both resources for growth but is limited by only one of them anywhere on its ZNGI. A family of generalist ZNGIs with increasingly sharp curvatures continuously span across these two endpoints (Fig. 1b,1c). The symmetry conditions described above entail that all generalist ZNGIs are symmetric about the diagonal *R*_1_ = *R*_2_.

We assume that a consumer can only increase its affinity for one resource at the expense of decreasing its affinity for the other. The trade‐off between *a*_1_ and *a*_2_ may favour either resource generalists (Fig. 1e–f) or resource specialists (Fig. 1g–h). Our assumption that resources can only differ with respect to affinity implies that all trade‐off curves are symmetrical about the diagonal *a*_1_ = *a*_2_.

We restrict our analyses to trade‐off curves and generalist ZNGIs that are either everywhere convex, everywhere concave, or linear, and fix all generalist ZNGIs and trade‐offs such that a single generalist consumer with affinities *a*_1_ = *a*_2_ = 1 can always persist at the resource densities *R*_1_ = *R*_2_ = *R*_0_ for all combinations of trade‐offs and resource types. Consequently, all generalist ZNGIs pass through the point *R*_1_ = *R*_2_ = *R*_0_ and all trade‐offs pass through the point *a*_1_ = *a*_2_ = 1 (Fig. 1). We thus vary the trade‐off around a fixed midpoint rather than around two fixed endpoints. While the latter has been the convention in trade‐off studies considering substitutable resources, this convention is not suitable for essential resources, because no consumer would persist under strongly specialist‐favouring trade‐offs.

We use the framework of adaptive dynamics (Metz *et al.* 1992; Geritz *et al.* 1998; Dercole & Rinaldi 2008; Brännström *et al.* 2013) to study evolutionary community assembly as a function of resource type and trade‐off shape. We use this framework to find the maximal number of consumers that can coexist at an evolutionarily stable equilibrium, where different consumers are characterised by unique combinations of resource affinities. We require global evolutionary stability, so that no other affinity combination on the trade‐off curve can invade the community. We made the above assumptions about symmetries in the generalist ZNGIs and trade‐off curves, because they facilitate the derivation of analytical results. Specifically, they enable us to derive conditions for the feasibility of evolutionary coexistence in terms of the curvatures of the generalist ZNGI at the point *R*_1_ = *R*_2_ = *R*_0_ and of the trade‐off curve at *a*_1_ = *a*_2_ = 1 (Fig. 1). Yet, the symmetry assumptions also imply that the possibility for coexistence of different consumers is maximised (see Results and Appendix S3.2).

To illustrate our analytical results for homogeneous environments with numerical examples, and for comparison with the environmentally heterogeneous case, we implemented specific trade‐off, consumption and growth functions that allow resources to vary continuously from perfectly substitutable to fully essential. The specific model (described in Appendix S2.1.1) produces saturating growth similar to a Holling type II functional response when resources are perfectly substitutable, and approaches a saturating minimum model as resources approach perfect essentiality. The numerical methods used to solve for the evolutionarily coexisting consumer community are described in Appendix S2.2.1.

### Heterogeneous environments

**Figure 2:**
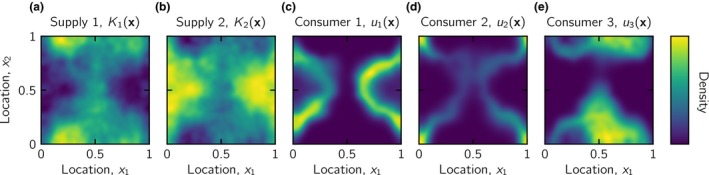
A spatially heterogeneous resource supply landscape and the distributions of three consumers from a resulting evolutionarily stable community. Resource supply densities for (a) resource 1, *K*_1_(**x**), and (b) resource 2, *K*_2_(**x**). Both resource supply densities satisfy (1/*L* ^2^) ∫ *K_j_*(**x**)d**x** = *K*, where *L* = 1 is the length of the side of the square landscape, and Corr(*K*_1_, *K*_2_) = − 1, implying that the supplies are entirely anticorrelated. (c–e) Spatial distributions of three consumers picked from the evolutionarily stable community of seven consumers, where the normalised curvature of the generalist ZNGI is given by ${\hat{\kappa }}_{Z}\approx 1.76$ (i.e. interactive‐essential resources), and the normalised curvature of the trade‐off curve is given by ${\hat{\kappa }}_{T}\approx 0.396$ (a weakly specialist‐favouring trade‐off). Consumer 1 is more specialised on resource 1 with affinities (*a*_1_, *a*_2_) ≈ (1.4, 0.64), consumer 2 is a generalist with affinities (*a*_1_, *a*_2_) ≈ (1.0, 1.0) and consumer 3 is more specialised on resource 2 with affinities (*a*_1_, *a*_2_) ≈ (0.59, 1.5). The colour scale is relative within each panel.

We introduce spatial heterogeneity into eqns 1 by letting the supply of resources and the densities of consumers and resources vary in space, and by allowing random, diffusive movement/transport of consumers and resources. We cannot treat the resulting partial differential equation system completely analytically and therefore solve a specific system numerically. We let the dynamics take place on a unit square with periodic boundaries and spatial coordinates **x** = (*x*_1_, *x*_2_). We randomly generated nine different landscapes where the local supplies of the two resources, *K*_1_(**x**) and *K*_2_(**x**), varied smoothly in space (either independently or with positive or negative correlation), but the mean supplies were the same as in the homogeneous environment. Technically, this means that (1/*L* ^2^) ∫ *K*_1_(**x**)d**x** = (1/*L* ^2^) ∫ *K*_2_(**x**)d**x** = *K*, where $0<x_{1}\leq 1$, $0<x_{2}\leq 1$ and *L* = 1 is the side length of the unit square‐sized landscape. The details of landscape generation are described in Appendix S2.2.2 and an example is depicted in Fig. 2a,b. The equations describing per capita growth functions, proportional consumptions and trade‐offs (eqns S2.7) are identical to the numerical examples for the homogeneous environments (eqns S2.1). When rates of diffusion become very large, the heterogeneous model therefore approaches the homogeneous model.

We assemble communities in the same way as for the homogeneous environment, until the ensemble of consumers is globally closed to invasion by any other affinity combination on the trade‐off curve, and we have the maximal number of consumers that can evolutionarily stably coexist (see Appendix S2.2.2 for numerical implementation). As local adaptation is possible, this often results in communities that have several consumers with different spatial distributions (see examples in Fig. 2c–e).

## Results

### Homogeneous environments

Two major results arise from our analyses of evolutionary community assembly in homogeneous environments.

**Figure 3:**
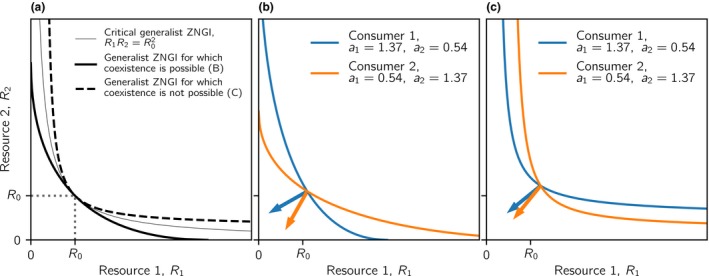
Examples of generalist ZNGIs and corresponding ZNGIs of non‐generalist consumers leading to ecologically stable vs. unstable coexistence in homogeneous environments. (a) Critical generalist ZNGI given by $R_{1}R_{2}=R_{0}^{2}$ (thin gray line). If a generalist ZNGI lies entirely above this critical generalist ZNGI (e.g. dashed line), resources are too essential and ecologically stable coexistence is not possible. If a generalist ZNGI lies entirely below the critical generalist ZNGI (e.g. black solid line), resources are sufficiently substitutable and ecologically stable coexistence is possible. (b) ZNGIs for substitutable resources derived from the black solid line in panel (a), leading to ecologically stable coexistence. (c) ZNGIs for essential resources derived from the dashed line in panel (a), leading to unstable coexistence. Consumer 1 is more specialised on resource 1 (blue ZNGIs, *a*_1_ = 1.37, *a*_2_ = 0.54) and consumer 2 is more specialised on resource 2 (orange ZNGI, *a*_1_ = 0.54, *a*_2_ = 1.37). Equilibrium resource densities, $R_{j}^{*}$, are indicated by the intersections of the ZNGIs. Consumption vectors − (*C*_1_, *C*_2_), illustrating the relative consumption rates of resources 1 and 2, are indicated by arrows with slopes − *C*_2_/*C*_1_. Because $R_{1}^{*}=R_{2}^{*}$, the consumption vectors of consumer 1 (blue arrows) have relatively shallower slopes than the consumption vectors of consumer 2 (orange arrows), which in combination with the relative slopes of the ZNGIs at their intersection implies that coexistence is stable in B, but not in C. Resource supply densities are *K*_1_ = *K*_2_ = 1.

First, stable ecological coexistence is only possible when resources are sufficiently substitutable (e.g. Fig. 1a,b). The exact definition of ‘sufficiently substitutable’ is given by what we subsequently call the *critical generalist ZNGI*, which is the ZNGI of a generalist consumer (with affinities *a*_1_ = *a*_2_) described by $R_{1}R_{2}=R_{0}^{2}$ (Fig. 3a). Resource types characterised by generalist ZNGIs entirely below this curve permit ecological coexistence, whereas resource types characterised by generalist ZNGIs entirely above this curve preclude ecological coexistence (Fig. 3). The reason is that only sufficiently substitutable resources fulfil a necessary condition for stable ecological coexistence: each consumer must consume relatively more of the resource that relatively more limits its own growth (Tilman 1980). This is illustrated with an example of two consumers with opposite affinities (Fig. 3b–c). Consumer 1 has higher affinity for resource 1, and therefore consumes relatively more of resource 1 (indicated by a shallower consumption vector with slope − *C*_2_/*C*_1_) than consumer 2. Compared to consumer 2, consumer 1 is, at ecological equilibrium, more limited by resource 1 when resources are substitutable, but more limited by resource 2 when resources are essential, as indicated by a reversion in the order of the consumers’ ZNGIs between the panels (see formal derivation in Appendix S1.1.3). Consequently, the equilibrium is stable when resources are substitutable (Fig. 3b), but unstable when resources are essential (Fig. 3c). The switch in relative limitation from resource 1 to resource 2 occurs at exactly the level of resource essentiality described by the critical generalist ZNGI (see Appendix S1.1 for a formal derivation). Note that this result hinges critically on our assumption that consumers consume more of the resource with higher effective availability *a_ij_R_j_*. We address the generality of this assumption in the discussion section ‘Critical essentiality’.

Second, conditions for evolutionary coexistence are more restrictive than conditions for ecological coexistence. Specifically, evolutionarily stable coexistence requires that ecological coexistence is possible and that the trade‐off in affinities is sufficiently specialist‐favouring. To make precise this notion, we classify resources on the substitutable–essential spectrum by the curvature of the generalist ZNGI at the point *R*_1_ = *R*_2_ = *R*_0_, multiplied by $R_{0}\sqrt{2}$. We label this the *normalised curvature* ${\hat{\kappa }}_{Z}$. When ${\hat{\kappa }}_{Z}=0$ (Fig. 1a), resources are perfectly substitutable. As ${\hat{\kappa }}_{Z}$ gets bigger, resources become less substitutable (Fig. 1b,c), until as ${\hat{\kappa }}_{Z}\to \infty$, resources become strictly essential (Fig. 1d). Similarly, we classify the affinity trade‐off by its *normalised curvature* ${\hat{\kappa }}_{T}$ (=curvature of the trade‐off curve multiplied by $\sqrt{2}$) at the point *a*_1_ = *a*_2_ = 1. The more negative ${\hat{\kappa }}_{T}$ is, the more are resource generalists favoured (Fig. 1e,f), and the more positive ${\hat{\kappa }}_{T}$ is, the more are resource specialists favoured (Fig. 1g,h).

**Figure 4:**
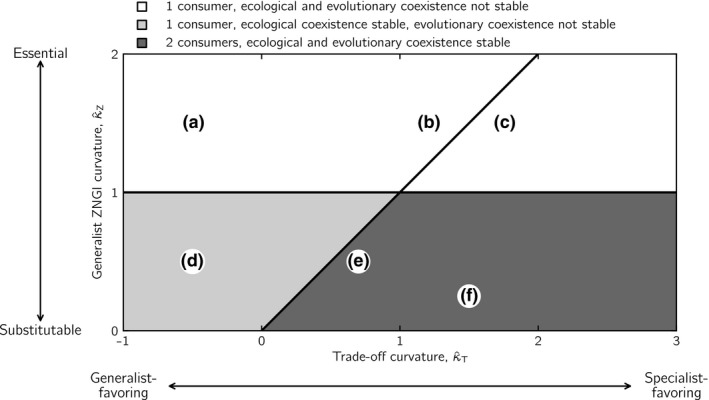
Ecological and evolutionary outcomes of competition for two resources in homogeneous environments as a function of the resource type (substitutable to essential) and the shape of the affinity trade‐off (generalist‐ to specialist‐favouring). On the *x*‐axis, a family of trade‐off curves characterised by their normalised curvature ${\hat{\kappa }}_{T}$ at the point *a*_1_ = *a*_2_ = 1 in affinity space go from being generalist‐favouring for ${\hat{\kappa }}_{T}<0$ to being increasingly specialist‐favouring for ${\hat{\kappa }}_{T}>0$. On the *y*‐axis, a family of generalist ZNGIs characterised by their normalised curvature ${\hat{\kappa }}_{Z}$ at the point *R*_1_ = *R*_2_ = *R*_0_ go from perfectly substitutable resources for ${\hat{\kappa }}_{Z}=0$ to increasingly essential resources as ${\hat{\kappa }}_{Z}$ increases. The lines ${\hat{\kappa }}_{Z}=1$ and ${\hat{\kappa }}_{Z}={\hat{\kappa }}_{T}$ separate different outcomes. Ecologically stable coexistence is possible when ${\hat{\kappa }}_{Z}<1$. Evolutionarily stable coexistence is possible when ${\hat{\kappa }}_{Z}<1$ and ${\hat{\kappa }}_{Z}<{\hat{\kappa }}_{T}$. See Fig. 1 for examples of generalist ZNGIs and trade‐off curves corresponding to specific numerical values of ${\hat{\kappa }}_{Z}$ and ${\hat{\kappa }}_{T}$. The letters (a)–(f) cross‐reference the generalist ZNGI and trade‐off curvatures which lead to the evolutionary outcomes in the corresponding panels of Fig. 5.

Using these classifications, we derive analytically in Appendix S1.1 two conditions for evolutionarily stable coexistence (Fig. 4). First, the normalised curvature of the critical generalist ZNGI, $R_{1}R_{2}=R_{0}^{2}$, is ${\hat{\kappa }}_{Z}^{\text{crit}}=1$. Hence, when ${\hat{\kappa }}_{Z}>1$, resources are too essential, and ecologically stable coexistence is impossible. Consequently, evolutionarily stable coexistence is also impossible. Second, when ${\hat{\kappa }}_{Z}<1$ and ${\hat{\kappa }}_{T}<{\hat{\kappa }}_{Z}$, ecologically stable coexistence is possible, but evolution will either drive one of the coexisting consumers to extinction, or both consumers will converge onto the same phenotype. Consequently, evolutionarily stable coexistence occurs if and only if resources are sufficiently substitutable (${\hat{\kappa }}_{Z}<1$) and trade‐offs are sufficiently specialist‐favouring (${\hat{\kappa }}_{Z}<{\hat{\kappa }}_{T}$, Fig. 4).

**Figure 5:**
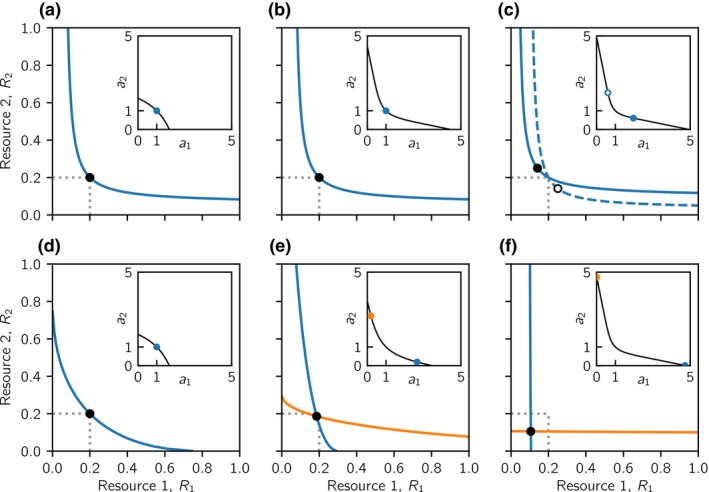
Examples of evolutionary outcomes for different resource types and trade‐offs in homogeneous environments. Shown are zero net growth isoclines (ZNGIs) and trade‐off curves of consumers in evolutionarily stable communities. Panels (a)–(f) cross‐reference Fig. 4 for the combinations of resource types (normalised curvatures ${\hat{\kappa }}_{Z}$ of the generalist ZNGI) and normalised trade‐off curvatures ${\hat{\kappa }}_{T}$ that were used in evolutionary community assembly. The large panels show the ZNGIs of the consumer(s) in resource space as curves, and the resource densities at the evolutionary equilibrium as dots. The insets show the trade‐off between resource affinities *a*_1_ and *a*_2_ for all consumers as curves, and the affinity combination(s) of the consumer(s) at the evolutionary equilibrium as dots. In panel (c), two alternative stable states are depicted. Initial conditions *a*_1_ > *a*_2_ produce the outcome depicted by the solid line and filled dots, whereas initial conditions *a*_2_ > *a*_1_ produce the outcome depicted by the broken line and open dots. For the specific family of per capita growth functions and parameter values used in the numerical simulations, *R*_0_ = 0.2. Resource supply densities are *K*_1_ = *K*_2_ = 1.

The numerical examples in Fig. 5 illustrate the analytical results. When resources are sufficiently essential, evolution always selects for a single consumer (Fig. 5a–c). For sufficiently generalist‐favouring trade‐offs this single consumer will be a generalist (Fig. 5a,b), whereas strongly specialist‐favouring trade‐offs will result in evolutionary bi‐stability with a single intermediate specialist (Fig. 5c). When resources are sufficiently substitutable, sufficiently specialist‐favouring trade‐offs select for coexistence of two specialist consumers (Fig. 5e,f). In contrast, a generalist‐favouring trade‐off will drive evolution into a single generalist consumer, as illustrated with an example of ecologically stable coexistence that is evolutionarily unstable and converges onto a single generalist phenotype (Figs 3b and 5d).

The results above rely on the various symmetries that follow from our assumption that consumers and resources only differ in affinities. While this assumption may seem restrictive, we show numerically in Appendix S3.2 that the symmetric case serves as a very good representation, and that the above results are qualitatively (and often quantitatively) robust to various asymmetries. As expected, any imposed asymmetries (such as skewed resource supply ratios or a skewed affinity trade‐off) tend to reduce the parameter space yielding evolutionarily stable coexistence between two types of consumers (Figs S3.2–S3.9).

### Heterogeneous environments

**Figure 6:**
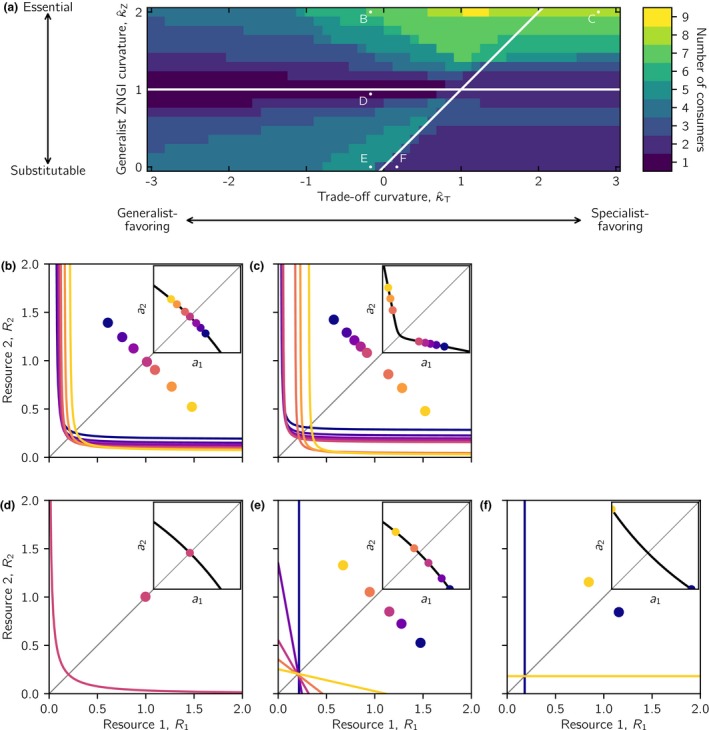
Evolutionary outcomes of competition for two resources in heterogeneous environments. (a) Consumer diversity as a function of the resource type (substitutable to essential) and the shape of the affinity trade‐off (generalist‐ to specialist‐favouring), with the axes being quantified in units of the normlised curvatures of the trade‐off curve ${\hat{\kappa }}_{T}$ and the generalist ZNGI ${\hat{\kappa }}_{Z}$ (see Fig. 4 for details). The horizontal and diagonal lines depict the relations ${\hat{\kappa }}_{Z}=1$ and ${\hat{\kappa }}_{Z}={\hat{\kappa }}_{T}$ respectively. Each square is the outcome of a numerical simulation, where the colour of the square indicates the number of evolutionarily stably coexisting consumers on the entire landscape. The letters B–F cross‐reference the generalist ZNGI and trade‐off curvatures which lead to the evolutionary outcomes in panels (b)–(f). (b–f) ZNGIs and *effective supply points* of all consumers in resource space as curves and dots respectively. An *effective supply point* is the average resource supply $\left({\overset{¯}{K}}_{i1},\;{\overset{¯}{K}}_{i2}\right)$ experienced by consumer *i* across the entire landscape, weighted by its local abundances, that is, ${\overset{¯}{K}}_{\mathrm{ij}}={\left(\int u_{i}\left(x\right)dx\right)}^{-1}\int u_{i}\left(x\right)K_{j}\left(x\right)dx$. The insets show the trade‐off between resource affinities *a*_1_ and *a*_2_ for all consumers as curves, and the affinity combination(s) of the consumer(s) at the evolutionary equilibrium as dots. Note that the effective supply points of resource specialists (e.g. yellow dots) in panels (b) and (e) are on opposite sides of the diagonal in resource space, illustrating that consumers are most abundant in habitats where the supply of their preferred resource is either highest (substitutable resources) or lowest (essential resources). In each of panels (b)–(f), the *outer geometrical envelope* would be the line tracing the parts of the ZNGIs that are closest to the axes.

In heterogeneous environments, the outcome of evolutionary community assembly also depends on the degree to which resources are substitutable/essential and on the shape of the affinity trade‐off in ways that can be described by the curvatures of the generalist ZNGI (${\hat{\kappa }}_{Z}$) and the trade‐off curve (${\hat{\kappa }}_{T}$). In all nine resource‐supply landscapes that we explored, we found three distinct diversity patterns. Below, we describe these patterns and illustrate them with a representative example of a landscape in which local supply rates of resources 1 and 2 are anticorrelated (Fig. 2). Interestingly, the lines ${\hat{\kappa }}_{Z}=1$ and ${\hat{\kappa }}_{Z}={\hat{\kappa }}_{T}$ help us again to understand the evolutionary outcomes, albeit for different reasons than in homogeneous environments (Fig. 6, see Appendix S3.3 for the remaining eight resource–supply landscapes).

First, under most conditions, evolved consumer diversity is higher in heterogeneous than in homogeneous environments (Fig. 6a). Intriguingly, however, the conditions promoting coexistence in homogeneous environments – substitutable resources and specialist‐favouring trade‐offs – engender among the least diverse communities in heterogeneous environments. To understand this, consider the bottom row in Fig. 6a, where resources are perfectly substitutable. If the trade‐off favours specialisation, the two extreme specialists will be selected for (Fig. 6f). These deplete their respective resources everywhere on the landscape to levels that make it impossible for any other affinity combination to persist. Such resource monopolisation is, however, not possible when trade‐offs favour generalists or when resources are essential. In fact, the highest consumer diversity is attained when resources are essential (Fig. 6a), that is, under conditions that prevent ecological coexistence in homogeneous environments.

Second, highly diverse communities only form for resources that are clearly substitutable or clearly essential. In contrast, diversity is lowest for intermediate resource types (near the horizontal line ${\hat{\kappa }}_{Z}=1$, Fig. 6a), where a regime shift in local adaptation occurs along the resource substitutability axis. When resources are substitutable, consumers adapt locally to where their favoured resource is more abundant. Consequently, consumers with high affinity for resource 1 are abundant where the supply of resource 1 is high (Fig. 6e). In contrast, when resources are clearly essential, consumers adapt locally to where their favoured resource is scarce. Consequently, consumers with high affinity for resource 1 are abundant where the supply of resource 1 is low (Fig. 6b). The switch between these contrasting local selection regimes occurs when resources are of intermediate type (${\hat{\kappa }}_{Z}=1$ in Fig. 6a). For such resource types, the possibilities for local adaptation through specialisation are strongly limited, because either a perfect generalist (for ${\hat{\kappa }}_{T}$ approximately ≤ 0), or a pair of mild specialists (for ${\hat{\kappa }}_{T}$ approximately > 0), outcompete all other phenotypes everywhere in the landscape, regardless of the local resource supply ratios (Fig. 6d, see Appendix S1.2.1 for technical derivations). Diversity increases away from this line in both directions, indicating that more consumers can locally adapt and coexist by partitioning space. The exception is the lower right corner of Fig. 6a, where the previously described monopolisation effects are operating.

Third, the most diverse communities evolve when trade‐offs are near the diagonal line ${\hat{\kappa }}_{Z}={\hat{\kappa }}_{T}$ (Fig. 6a). This can be understood as follows. When trade‐offs are strongly generalist‐favouring, highly specialised consumers cannot compete. Moving from left to right in Fig. 6a, the penalty on resource specialisation is gradually relaxed, and more specialist consumers can join the community. Diversity declines again when the trade‐off becomes too specialist‐favouring, but the underlying mechanisms differ for substitutable vs. essential resources.

For substitutable resources (${\hat{\kappa }}_{Z}<1$), diversity declines abruptly at the diagonal ${\hat{\kappa }}_{Z}={\hat{\kappa }}_{T}$, where the system undergoes a shift in selection regime. To understand this regime shift, we use ideas from Wickman *et al.* (2017), who showed that selection in spatially heterogeneous systems described by reaction‐diffusion equations can be divided into sympatric and parapatric selection terms. Sympatric selection is a spatially weighted average of local disrupting or stabilising selection, and parapatric selection is, roughly speaking, a measure of the variance in local directional selection. Analysing sympatric selection on a single consumer at the evolutionarily singular point close to (*a*_1_, *a*_2_) = (1, 1), we notice that sympatric selection goes from stabilising to disruptive close to the line ${\hat{\kappa }}_{Z}={\hat{\kappa }}_{T}$ when we cross from left to right (Fig. 6a). Hence, evolutionary dynamics change qualitatively when this line is crossed from a regime where diversity is generated through local adaptation (Fig. 6e) to a regime where the community is dominated by a few global resource specialists (Fig. 6f).

For essential resources (${\hat{\kappa }}_{Z}>1$), a maximum or plateau in diversity is reached to the left of the diagonal ${\hat{\kappa }}_{Z}={\hat{\kappa }}_{T}$. Because essential resources cannot be monopolised by extreme specialists, a regime shift does not occur. Yet, as trade‐offs become more specialist‐favouring, the community loses generalist consumers, and all consumers become specialised to varying degrees (Fig. 6c). The most diverse communities form when the trade‐off strikes a balance allowing both generalists and specialists to coexist.

## Discussion

In this paper, we investigated how selection on resource‐uptake affinities shapes the evolution of competitive communities. We focused on affinity because it is at the very core of resource competition, affecting for any phenotype both its effects on and its responses to resource levels. We explored the evolution of uptake affinities comprehensively across a two‐factorial continuum from substitutable to essential resources and from generalist‐ to specialist‐favouring trade‐offs, and did so in spatially homogeneous and heterogeneous landscapes.

### Homogeneous environments – generalisations of earlier findings

In homogeneous environments, we showed that evolutionarily stable coexistence requires both sufficiently substitutable resources and sufficiently specialist‐favouring trade‐offs. This finding provides context and deeper understanding to earlier studies. Schreiber & Tobiason (2003) studied competition for antagonistic to essential resources under a linear affinity trade‐off and found that evolutionarily stable coexistence is possible only for perfectly substitutable or antagonistic resources. This corresponds to tracing a vertical line at ${\hat{\kappa }}_{T}=0$ in Fig. 4. In Appendix S3.1, we show that our results also extend to antagonistic resources. Similarly, Levins (1962), MacArthur & Levins (1964), Lawlor & Smith (1976), and Rueffler *et al.* (2006) studied competition for two perfectly substitutable resources, and found that coexistence required a specialist‐favouring trade‐off. This corresponds to tracing a horizontal line at ${\hat{\kappa }}_{Z}=0$ in Fig. 4. Our findings for homogeneous environments thus unify and put into a single framework two disparate strands of evolutionary theory of resource specialisation, and map out the previously uncharted territory in resource type–trade‐off space that surrounds these earlier studies.

### Critical essentiality

Another important discovery is the threshold of ‘critical essentiality’ of resources beyond which ecological (and thus evolutionary) coexistence is impossible in homogeneous environments. On a first glance, this result seems surprising, given that coexistence of competitors for strictly essential resources is a textbook example of resource competition theory (e.g. Begon *et al.* 2006). Yet, the result only extends earlier findings. León & Tumpson (1975) showed that stable ecological coexistence of two resource competitors requires that each competitor removes more of the resource which contributes more to its own growth. For substitutable resources, this can be accomplished by differences in affinity alone. In contrast, when background mortalities of consumers are equal, differences in affinity alone cannot engender coexistence if resources are strictly essential; in that case differences in the ratio of conversion efficiencies are required (León & Tumpson 1975; Abrams 1988; Vincent *et al.* 1996).

The finding of a threshold of critical resource essentiality hinges on our assumption that consumers remove relatively more of the resource that has the higher effective availability *a_ij_R_j_*. While this assumption is straightforward for substitutable resources, it may not necessarily apply to essential resources. For example, the uptake of essential nutrients by primary producers can be dictated by the elemental stoichiometry of the producer rather than by effective nutrient availability (Grover *et al.* 1997). In that case, the assumption that consumers take up proportionally more of the resources for which *a_ij_R_j_* is the highest can be violated, because the consumption of a non‐limiting nutrient is entirely governed by the effective availability of the limiting nutrient and the consumer's elemental stoichiometry. Ecological coexistence then becomes possible if consumers are limited by different nutrients and are appropriately niche‐differentiated with respect to elemental stoichiometry (Tilman 1980).

This raises the question: when resources are essential, what traits are selected for if both elemental stoichiometry and uptake affinity can evolve? In models where both uptake affinity and elemental stoichiometry of a monomorphic consumer can vary adaptively, the resulting optimal phenotype adjusts its uptake and stoichiometry such that growth is exactly co‐limited (Branco *et al.* 2018). Graphically, this means that the optimal phenotype consumes resources in a ratio pointing from the supply point to the corner of its ZNGI. Under such conditions, any given supply point would select for a single optimal phenotype, making coexistence of competitors for essential resources evolutionarily implausible.

In conclusion, our results suggest that, if affinity is the main trait under selection, there will often be a sharp transition from ‘substitutable‐like’ to ‘essential‐like’ resources where the qualitative dynamics of the system change. An important novelty is that we can identify a critical generalist ZNGI where this transition occurs. Although the specific form of the critical generalist ZNGI depends on the specific form of the per capita growth functions, we suggest that the phenomenon of two distinct competitive regimes for substitutable‐ vs. essential‐like resources is a general principle (see also Abrams 1988). Interestingly, this critical ZNGI is crucial to an understanding of the evolution of resource specialisation also in spatially heterogeneous environments.

### Evolution of metacommunities in heterogeneous landscapes

In spatially heterogeneous environments, several consumers can coexist in competition for two resources, with the most diverse communities evolving for combinations of either clearly substitutable resources and near‐linear trade‐offs or clearly essential resources and moderately specialist‐favouring trade‐offs. We explained these findings using the curvatures of the generalist ZNGI and the trade‐off curve. Koffel *et al.* (2016) recently developed a complementary framework, which uses the curvature of the outer envelope tracing the ZNGIs of competitors (see caption of Fig. 6) to predict the evolutionary stable trait composition of metacommunities in spatially variable resource supply landscapes. We discuss links between the two approaches in Appendix S1.2.

The degree to which environments can be considered heterogeneous depends not only on local differences in resource supply but also on the movement rates of consumers and resources. Ecologically, low or no movement corresponds to the species sorting scenario of metacommunity theory (Leibold *et al.* 2004), for which regional coexistence of many resource competitors is possible if the landscape contains a broad range of local resource supply ratios (Tilman 1982). Wickman *et al.* (2017) showed that this also holds evolutionarily for perfectly substitutable resources and generalist‐favouring trade‐offs. The number of evolutionarily coexisting consumers increases as diffusion rates decrease, and as diffusion rates approach zero, an infinite number of consumers can coexist, which adapt locally to a continuum of spatially varying supply ratios (Wickman *et al.* 2017).

Alternative states at the local level have been debated as another potential mechanism sustaining regional metacommunity diversity (Chase 2010; Fukami 2015). In homogeneous environments, we found that essential resources and specialist‐favouring trade‐offs engender the evolution of alternative states that are dominated by one of two specialists with contrasting affinities (Fig. 5c). Essential resources and specialist‐favouring trade‐offs might therefore promote the evolution of local alternative states also in heterogeneous landscapes. These conditions do indeed engender diverse metacommunities of specialists from which generalists are conspicuously absent (Fig. 6c). Intriguingly, these regionally coexisting specialists show low spatial overlap (results not shown), suggesting that the evolution of local alternative states merits further exploration.

### Sympatric vs. parapatric diversification

For perfectly substitutable resources and specialist‐favouring trade‐offs, neither spatial heterogeneity nor rates of diffusion matter for the outcome, which will always be two consumers, each only consuming one of the resources. The mechanism generating this two‐consumer metacommunity can thus be said to be sympatric. The debate over the feasibility of sympatric diversification (and speciation) is long‐standing (Dieckmann & Doebeli 1999; Via 2001; Sousa & Hey 2013; Gavrilets 2014). Here, we surprisingly find that the conditions promoting diversification in sympatry may actually prevent greater diversification in parapatry, because, under a specialist‐favouring trade‐off, two global specialists can together monopolise two substitutable resources everywhere in a landscape of locally varying supply ratios. This tension between sympatric and parapatric mechanisms of trait diversification has gone unseen in studies which have modelled the evolution of consumer traits when resources are continuously distributed (e.g. seeds of different sizes) (Dieckmann & Doebeli 1999; Doebeli & Dieckmann 2000; Doebeli 2011). In such models, trait evolution affects the abilities of consumers to exploit different parts of the resource spectrum. Such models have also been used to study evolution in heterogeneous environments (Doebeli & Dieckmann 2003; Haller *et al.* 2013) and Pontarp *et al.* (2015) found that conditions that generate diversity in sympatry also generate greater diversity in parapatry. This contrast with our model comes about through the lack of a monopolisation effect, which can only operate when resources are discrete. As many consumer–resource interactions in nature are well described using discrete resources, this effect may play an important role in many eco‐evolutionary systems.

Taken together, our results provide a new picture of specialisation‐mediated, evolutionarily stable diversity of consumers competing for limiting resources. By considering a continuum of resource types and trade‐offs, we showed that substitutable and essential resources give rise to qualitatively different coexistence regimes, and that the consequences of this dichotomy are very different in spatially homogeneous and heterogeneous environments. Since resource competition is a fundamental ecological interaction, the insights presented here should be applicable to a broad range of systems.

## Author Contributions

JW conceived and designed the study with input from AB and SD. JW performed the mathematical and numerical analysis. JW drafted the manuscript with revisions from SD and comments from AB.

## Data Accessibility Statement

The data for the evolutionarily stable metacommunities have been deposited to the Dryad digital repository, with the DOI being: https://doi.org/10.5061/dryad.js18230.
