# Supplementary material for: Evolution of resource specialisation in competitive metacommunities
Source: Ecol Lett. 2019 Aug 7;22(11):1746–56. doi: 10.1111/ele.13338 (PMC6852178; doi:10.1111/ele.13338)
Supplement: Supplementary file 1 [file ELE-22-1746-s001.pdf]

Appendix S1: Derivation of analytical results

In this appendix we derive the analytical results for the homogeneous model, and some limited results for the heterogeneous model.

S1.1 Homogeneous environments

For spatially homogeneous systems, we study the model

$$\frac{\mathrm{d}u_i}{\mathrm{d}t} = (G(a_{i1}R_1, a_{i2}R_2) - \mu)u_i \tag{S1.1a}$$

$$\frac{\mathrm{d}R_1}{\mathrm{d}t} = 1 - R_1 - \sum_i C_1(a_{i1}R_1, a_{i2}R_2)G(a_{i1}R_1, a_{i2}R_2)u_i \tag{S1.1b}$$

$$\frac{\mathrm{d}R_2}{\mathrm{d}t} = 1 - R_2 - \sum_i C_2(a_{i1}R_1, a_{i2}R_2)G(a_{i1}R_1, a_{i2}R_2)u_i\,. \tag{S1.1c}$$

Here we have assumed for simplicity that the supply rate as well as the renewal rate of both resources are 1 (this is only a matter of scaling). a_1 and a_2 are the affinities for resources 1 and 2, and are nonnegative. We assume that trade-off curves pass through the point $a_1 = a_2 = 1$, and are symmetric around the diagonal line in the a_1a_2-plane. For a *generalist consumer*, i.e., a consumer with $a_1 = a_2 = 1$, we assume that the zero net growth isocline (ZNGI) passes through the point $R_1 = R_2 = R_0$, and that this *generalist ZNGI* is symmetric around the diagonal in the R_1R_2-plane, or stated differently, that $G(X,Y) = G(Y,X)$. Note that this assumption implies that the function G is the same function for all consumers, and the only traits that differentiate the consumers are their affinities. Also note that if $X = Y = R_0$, then $G(X,Y) - \mu = 0$. Furthermore we assume that $C_1 + C_2 \equiv 1$, and that $C_1 \geq 0$ and $C_2 \geq 0$. Since we are interested in the evolution of resource uptake, we also assume that $\partial C_1/\partial(a_1R_1) \geq 0$ and $\partial C_2/\partial(a_2R_2) \geq 0$, and that $a_1R_1 \geq a_2R_2 \implies C_1 \geq C_2$.

Given these premises, we will show that there exists a generalist ZNGI, which we term the "critical generalist ZNGI", that separates two distinct ecological and evolutionary regimes. This critical generalist ZNGI is given by $R_1R_2 = R_0^2$ when $a_1 = a_2 = 1$. We will show that for generalist ZNGIs lying entirely below this curve, evolutionarily stable coexistence between two consumers is always possible given a sufficiently specialist-favoring trade-off. Furthermore, we will show that resource types characterized by generalist ZNGIs lying entirely above this critical curve do not permit stable ecological coexistence between two consumers. For the purposes of our investigations, we can restate this as follows. If the zero level curve of $G(X,Y) - \mu$ lies entirely below the critical level curve $XY = R_0^2$, then evolutionarily stable coexistence is always possible given a sufficiently specialist-favoring trade-off. Conversely if the zero level curve of the function $G(X,Y) - \mu$ lies entirely above the critical level curve, then ecological coexistence is impossible.

We do this by considering three different scenarios. First, we determine when evolutionary branchings are possible. We then determine when evolutionary branchings are not possible but evolutionarily stable coexistence nevertheless is. Finally, we determine when ecologically, and thus also evolutionarily, stable coexistence is impossible.

S1.1.1 Homogeneous environments: Evolutionary branchings

We start our investigations by determining when the point $(a_1, a_2) = (1, 1)$ will be an evolutionary branching point in the adaptive-dynamics sense (Geritz *et al.* 1998). We will show that if the generalist

ZNGI of the consumer lies entirely above the critical generalist ZNGI, evolutionary branching is not possible for any trade-off. Conversely we will show that if the generalist ZNGI of the consumer lies entirely below the critical generalist ZNGI, we can always find a trade-off that engenders evolutionary branching.

We consider the monomorphic one-consumer system

$$\frac{\mathrm{d}u}{\mathrm{d}t} = (G(a_1R_1, a_2R_2) - \mu)u \tag{S1.2a}$$

$$\frac{\mathrm{d}R_1}{\mathrm{d}t} = 1 - R_1 - C_1(a_1R_1, a_2R_2)Gu \tag{S1.2b}$$

$$\frac{\mathrm{d}R_2}{\mathrm{d}t} = 1 - R_2 - C_2(a_1R_1, a_2R_2)Gu\,. \tag{S1.2c}$$

We make the assumptions on the trade-off curves and ZNGIs as described after Eqs. S1.1. We first note that due to the symmetry of G, C_1, and C_2, if $a_1 = a_2 = 1$, then there must exist an equilibrium solution to the system above with $R_1 = R_2 = R_0$ (given that any positive solution for the consumer exists). For a generalist consumer to be in equilibrium, the equation for the ZNGI, $G(1 \cdot R_1, 1 \cdot R_2) - \mu = 0$ is satisfied. For any resource densities above this curve, the consumer would have positive growth rate. Since the resource densities are both R_0, this implies that we have a corresponding equation in affinity space, $G(a_1R_0, a_2R_0) - \mu = 0$, which will have the same shape as the generalist ZNGI, but scaled to go through the point $a_1 = a_2 = 1$. This curve is known as the invasion boundary (de Mazancourt & Dieckmann 2004). The invasion fitness of a rare mutant with a combination of affinities above this curve will be positive, whereas it will be negative for affinity combinations below this curve. For illustration, we show three examples of generalist ZNGIs (Fig. S1.1A) and their corresponding invasion boundaries (Fig. S1.1B). Since the generalist ZNGI is symmetric around the diagonal, so will the invasion boundary, and the slope of the invasion boundary at $a_1 = a_2 = 1$ will be -1. Since trade-off curves are assumed to be symmetric around the diagonal line in affinity space, any trade-off curve will also have a slope of -1 at $a_1 = a_2 = 1$. Since the invasion boundary and the trade-off curve have the same slope at this point, the point $(a_1, a_2) = (1, 1)$ is an evolutionarily singular point (de Mazancourt & Dieckmann 2004).

If the trade-off curve lies entirely below the invasion boundary, it is not possible for any mutant with a combination of affinities on the trade-off curve to invade the resident, and the evolutionarily singular point is an evolutionarily stable strategy (ESS). Locally around the point $a_1 = a_2 = 1$, the behavior of the system will be determined by the curvatures of three curves: the trade-off curve, the invasion boundary, and something known as the attainability boundary, which delineates the areas in affinity space that can be reached through gradual small mutations (de Mazancourt & Dieckmann 2004). We denote these curvatures at the point $a_1 = a_2 = 1$ by κ_T for the trade-off, κ_Z for the invasion boundary, and κ_A for the attainability boundary. The curvature of the invasion boundary κ_Z is thus a scaled version of what we call the generalist ZNGI curvature in the main text, scaled into affinity space. In the following derivations we will express all analytical results in terms of the curvatures κ_T and κ_Z. However, in the main text and in all figures (in both the main text and the appendices), we have scaled κ_T and κ_Z by a factor $\sqrt{2}$ to reduce notational clutter. Table S1.1 gives an overview over all symbols and definitions of scaled and unscaled curvatures used in the main text, figures and appendices.

By de Mazancourt & Dieckmann (2004), evolutionary branching occurs if and only if

$$\kappa_\mathrm{Z} < \kappa_\mathrm{T} < \kappa_\mathrm{A}\,. \tag{S1.3}$$

The curvatures κ_Z and κ_A can both be calculated from the generalist ZNGI, and the curvature κ_T can be chosen independently of these, by choosing some specific trade-off curve. Thus, whether evolutionary

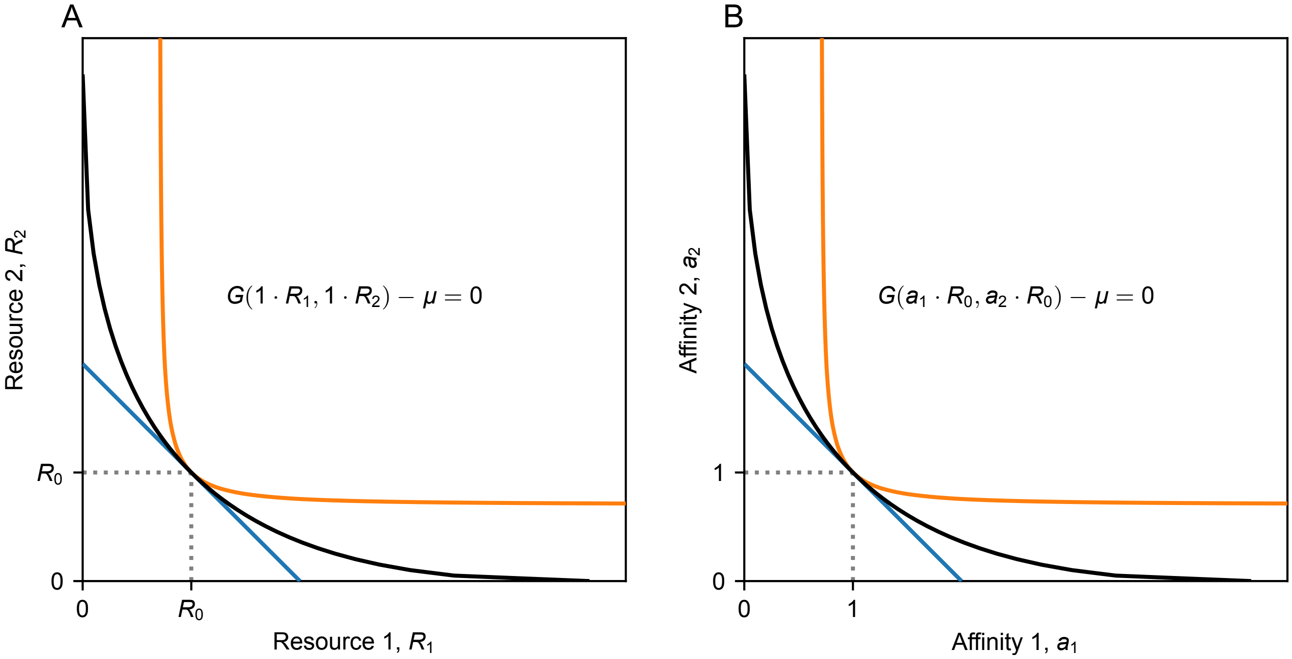

Figure S1.1: **A**: Examples of zero net growth isoclines (ZNGIs) in resource space for generalist consumers with affinities $a_1 = a_2 = 1$. **B**: Corresponding invasion boundaries for the consumers in affinity space in an equilibrium system where $R_1 = R_2 = R_0$. Due to the construction of G, the ZNGIs and the corresponding invasion boundaries have the same shape, and only the scaling of the axes is different. Three cases are illustrated where resources are assumed to be perfectly substitutable (blue lines), complementary substitutable (black lines), and interactively essential (orange lines).

Table S1.1: Symbols and definitions of curvatures used to characterize the trade-off curve, the generalist ZNGI, the invasion boundary, and the attainability boundary.

Symbol	Definition	Where it is used
κ_T	Unscaled curvature of the trade-off curve in affinity space at the point $(a_1, a_2) = (1, 1)$	Appendix S1.1.1 and S1.1.2
$\hat{\kappa}_\mathrm{T}$	Normalized curvature of the trade-off curve in affinity space at the point $(a_1, a_2) = (1, 1)$, scaled by $\sqrt{2}$; $\hat{\kappa}_\mathrm{T} = \sqrt{2}\kappa_\mathrm{T}$	Main text and all figures in main text and appendices
κ_U	Unscaled curvature of the generalist ZNGI in resource space at the point $(R_1, R_2) = (R_0, R_0)$	Derivation of invasion boundary curvature (Eq. S1.6)
$\kappa_\mathrm{U}^\mathrm{crit}$	Unscaled curvature of the critical generalist ZNGI in resource space at the point $(R_1, R_2) = (R_0, R_0)$; $\kappa_\mathrm{U}^\mathrm{crit} = 1/(\sqrt{2}R_0)$	Derivation of critical invasion boundary curvature (Eq. S1.18)
κ_Z	Curvature of the invasion boundary in affinity space at the point $(a_1, a_2) = (1, 1)$; equals the curvature of the generalist ZNGI scaled into affinity space; $\kappa_\mathrm{Z} = R_0\kappa_\mathrm{U}$	Appendix S1.1.1 and S1.1.2
$\kappa_\mathrm{Z}^\mathrm{crit}$	Curvature of the critical invasion boundary in affinity space at the point $(a_1, a_2) = (1, 1)$; $\kappa_\mathrm{Z}^\mathrm{crit} = 1/\sqrt{2}$	Eq. S1.18
$\hat{\kappa}_\mathrm{Z}$	Normalized curvature of the invasion boundary in affinity space at the point $(a_1, a_2) = (1, 1)$, scaled by $\sqrt{2}$; equals the normalized curvature of the generalist ZNGI scaled into affinity space; $\hat{\kappa}_\mathrm{Z} = \sqrt{2}\kappa_\mathrm{Z}$	Main text and all figures in main text and appendices
$\hat{\kappa}_\mathrm{Z}^\mathrm{crit}$	Normalized curvature of the critical generalist ZNGI at the point $(a_1, a_2) = (1, 1)$, scaled by $\sqrt{2}$; $\hat{\kappa}_\mathrm{Z}^\mathrm{crit} = 1$	Eq. S1.19
κ_A	Curvature of the attainability boundary in affinity space at the point $(a_1, a_2) = (1, 1)$	Appendix S1.1.1 and S1.1.2

branching is possible depends on whether $\kappa_Z < \kappa_A$, in which case we can always pick a trade-off such that κ_T satisfies Eq. S1.3.

As noted above, the invasion boundary has the same shape as the generalist ZNGI, but scaled so that it goes through the point $(1, 1)$ in affinity space, rather than (R_0, R_0) in resource space. This implies that, if the unscaled curvature of the generalist ZNGI in resource space is κ_U at the point (R_0, R_0), then the curvature of the invasion boundary at $(1, 1)$ in affinity space will be $\kappa_Z = R_0\kappa_U$. Hence, we start by computing κ_U in terms of derivatives of G. We let $X = a_1R_1$, and $Y = a_2R_2$. At the point $a_1 = a_2 = 1$, we have that $\partial G/\partial R_1 = \partial G/\partial X$ and $\partial G/\partial R_2 = \partial G/\partial Y$. The formula for the curvature of an implicit curve $f(x, y) = K$ is in general given by:

$$\kappa_f = -\frac{f_{xx}f_y^2 - 2f_{xy}f_xf_y + f_x^2 f_{yy}}{(f_x^2 + f_y^2)^{3/2}} \,. \tag{S1.4}$$

For our problem the symmetry yields that

$$\partial G/\partial X = \partial G/\partial Y =: G' \tag{S1.5}$$

for $a_1 = a_2 = 1$ and $R_1 = R_2 = R_0$. Throughout the rest of this derivation, we will take all derivatives to be evaluated for these values of the affinities and resources without further comment. Using the expression for the curvature of an implicit curve yields:

$$\kappa_U = -\frac{\frac{\partial^2 G}{\partial X^2}G'^2 - 2\frac{\partial^2 G}{\partial X\partial Y}G'G' + G'^2\frac{\partial^2 G}{\partial Y^2}}{(G'^2 + G'^2)^{3/2}} = -\frac{1}{2^{3/2}G'}\left(\frac{\partial^2 G}{\partial X^2} - 2\frac{\partial^2 G}{\partial X\partial Y} + \frac{\partial^2 G}{\partial Y^2}\right) . \tag{S1.6}$$

Since $\kappa_Z = R_0\kappa_U$, we thus have an expression for κ_Z:

$$\kappa_Z = -\frac{R_0}{2^{3/2}G'}\left(\frac{\partial^2 G}{\partial X^2} - 2\frac{\partial^2 G}{\partial X\partial Y} + \frac{\partial^2 G}{\partial Y^2}\right) . \tag{S1.7}$$

We can also derive an expression for the curvature of the attainability boundary, κ_A. By de Mazancourt & Dieckmann (2004) this is given by:

$$\kappa_A = -\frac{\frac{\partial S_A}{\partial a_1} + S_A\frac{\partial S_A}{\partial a_2}}{(1 + S_A^2)^{3/2}} \,, \quad S_A = -G_{a_1}/G_{a_2} \,, \tag{S1.8}$$

where G_{a_1} and G_{a_2} are the partial selection gradients at the point of interest. These are given by

$$G_{a_1} = \frac{\partial G}{\partial a_1} = \frac{\partial G}{\partial X}R_1 \tag{S1.9a}$$

$$G_{a_2} = \frac{\partial G}{\partial a_2} = \frac{\partial G}{\partial Y}R_2 \,. \tag{S1.9b}$$

Due to symmetry, $S_A = -1$ evaluated at $(a_1, a_2) = (1, 1)$. For notational convenience we let $R_s := \partial R_1/\partial a_1 = \partial R_2/\partial a_2$ and $R_d := \partial R_1/\partial a_2 = \partial R_2/\partial a_1$ and compute

$$\frac{\partial S_A}{\partial a_1} = \frac{\frac{\partial G_{a_1}}{\partial a_1}G_{a_2} - \frac{\partial G_{a_2}}{\partial a_1}G_{a_1}}{G_{a_2}^2} = \frac{1}{G'R_0}\left(\frac{\partial}{\partial a_1}\left(\frac{\partial G}{\partial X}R_1\right) - \frac{\partial}{\partial a_1}\left(\frac{\partial G}{\partial Y}R_2\right)\right) =$$
$$= \frac{1}{G'R_0}\left(\frac{\partial^2 G}{\partial X^2}(R_0 + R_s)R_0 - \frac{\partial^2 G}{\partial Y^2}R_0R_d + \frac{\partial^2 G}{\partial X\partial Y}(R_d - R_0 - R_s)R_0 + \frac{\partial G}{\partial X}R_s - \frac{\partial G}{\partial Y}R_d\right) \tag{S1.10a}$$

$$\frac{\partial S_A}{\partial a_2} = \frac{\frac{\partial G_{a_1}}{\partial a_2} G_{a_2} - \frac{\partial G_{a_2}}{\partial a_2} G_{a_1}}{G_{a_2}^2} = \frac{1}{G' R_0} \left(\frac{\partial}{\partial a_2} \left(\frac{\partial G}{\partial X} R_1 \right) - \frac{\partial}{\partial a_2} \left(\frac{\partial G}{\partial Y} R_2 \right) \right) =$$

$$= \frac{1}{G' R_0} \left(\frac{\partial^2 G}{\partial X^2} R_d R_0 - \frac{\partial^2 G}{\partial Y^2} (R_0 + R_s) R_0 + \frac{\partial^2 G}{\partial X \partial Y} (R_0 + R_s - R_d) R_0 + \frac{\partial G}{\partial X} R_d - \frac{\partial G}{\partial Y} R_s \right) . \tag{S1.10b}$$

This yields:

$$\kappa_{\mathrm{A}} = -\frac{1}{2^{3/2} G' R_0} \left(2G'(R_s - R_d) + (R_0 + R_s - R_d) R_0 \left(\frac{\partial^2 G}{\partial Y^2} - 2 \frac{\partial^2 G}{\partial X \partial Y} + \frac{\partial^2 G}{\partial Y^2} \right) \right) . \tag{S1.11}$$

To calculate R_s and R_d we investigate the equilibrium equations:

$$0 = (G - \mu) u \tag{S1.12a}$$

$$0 = 1 - R_1 - C_1 G u \tag{S1.12b}$$

$$0 = 1 - R_2 - C_2 G u \, . \tag{S1.12c}$$

Combining the second and third line, using that $C_1 + C_2 = 1$, and assuming $u > 0$ we get the equations

$$0 = (G - \mu) \tag{S1.13a}$$

$$0 = 1 - R_1 - C_1 (2 - R_1 - R_2) \, . \tag{S1.13b}$$

Differentiating the top equation with respect to a_1 yields:

$$0 = \frac{\partial G}{\partial a_1} = \frac{\partial G}{\partial X} \frac{\partial X}{\partial a_1} + \frac{\partial G}{\partial Y} \frac{\partial Y}{\partial a_1} = \frac{\partial G}{\partial X} (R_0 + R_s) + \frac{\partial G}{\partial Y} R_d \iff R_0 + R_s + R_d = 0 \, , \tag{S1.14}$$

using that $\partial G / \partial X = \partial G / \partial Y = G'$. Differentiating the bottom equation with respect to a_1 yields:

$$0 = -R_s - \frac{\partial C_1}{\partial a_1} (2 - 2R_0) - \frac{1}{2} (-R_s - R_d) =$$

$$= \frac{1}{2} (R_d - R_s) - \left(\frac{\partial C_1}{\partial X} (R_0 + R_s) + \frac{\partial C_1}{\partial Y} R_d \right) (2 - 2R_0) =$$

$$= \frac{1}{2} (R_d - R_s) - C' (R_0 + R_s - R_d)(2 - 2R_0) \, , \tag{S1.15}$$

where $C' := \partial C_1 / \partial X = \partial C_2 / \partial Y$, and we have used that $\partial C_1 / \partial Y = -\partial C_1 / \partial X$. Solving for R_s and R_d, we can compute:

$$R_s - R_d = \frac{4C' R_0 (R_0 - 1)}{1 + 4C'(1 - R_0)}, \quad R_0 + R_s - R_d = \frac{R_0}{1 + 4C'(1 - R_0)} \, . \tag{S1.16}$$

Rewriting our expression for κ_{A} (Eq. S1.11), we get:

$$\kappa_{\mathrm{A}} = -\frac{R_s - R_d}{\sqrt{2} R_0} - \frac{R_0 + R_s - R_d}{R_0} \frac{R_0}{2^{3/2} G'} \left(\frac{\partial^2 G}{\partial Y^2} - 2 \frac{\partial^2 G}{\partial X \partial Y} + \frac{\partial^2 G}{\partial Y^2} \right) =$$

$$= \frac{2\sqrt{2} C'(1 - R_0) + \kappa_{\mathrm{Z}}}{1 + 4C'(1 - R_0)} \iff$$

$$\iff \kappa_{\mathrm{A}} = \frac{\kappa_{\mathrm{Z}} + 2\sqrt{2} C'(1 - R_0)}{1 + 4C'(1 - R_0)} \, . \tag{S1.17}$$

Since $R_0 < 1$, and $C' \geq 0$, we can see that $\kappa_A > \kappa_Z$ for $\kappa_Z < 1/\sqrt{2}$, and vice versa, unless $C' = 0$, in which case $\kappa_A = \kappa_Z$. Since evolutionary branching is only possible when $\kappa_A > \kappa_Z$, we can conclude that evolutionary branching is only possible when $\kappa_Z < 1/\sqrt{2}$.

If the generalist ZNGI of the net growth function is given by the critical generalist ZNGI, $R_1 R_2 = R_0^2$, we can calculate the curvature of this to be $\kappa_U^{crit} = 1/(\sqrt{2} R_0)$, which implies that the curvature of the critical invasion boundary will be

$$\kappa_Z^{crit} = 1/\sqrt{2}\,. \tag{S1.18}$$

Hence, if a generalist ZNGI lies entirely below the critical generalist ZNGI, its curvature will be less than the curvature of the critical generalist ZNGI, which in turn means that the curvature of the associated invasion boundary will be less than $1/\sqrt{2}$. So, whenever a generalist ZNGI lies entirely below the critical generalist ZNGI, evolutionary branching is always possible, and will occur for a suitably chosen trade-off curve.

To simplify notation in the main text and in figures involving curvatures, we introduce the following normalized curvatures of ZNGIs and trade-off curves

$$\hat{\kappa}_T = \sqrt{2}\kappa_T\,, \quad \hat{\kappa}_Z = \sqrt{2}\kappa_Z\,, \quad \hat{\kappa}_Z^{crit} = \sqrt{2}\kappa_Z^{crit} = 1\,. \tag{S1.19}$$

In the main text and in all figures (including appendix figures), we use only these normalized curvatures. For notational consistency, however, we continue to use the original curvatures κ_T, κ_Z and κ_A in all following analytical derivations. Table S1.1 summarizes all symbols, definitions and scaling relationships between the different curvatures that are used in the main text and in the appendices.

S1.1.2 Homogeneous environments: Coexistence for trade-offs that do not yield branching

Here we will show that, if the generalist ZNGI of the consumers lies entirely below the critical generalist ZNGI, evolutionarily stable coexistence is possible for sufficiently specialist-favoring trade-offs, even if evolutionary branching does not occur.

Above, we showed that if $\kappa_Z < 1/\sqrt{2}$, then evolutionary branching leading to evolutionary coexistence is possible, and that this requires that the curvature of the invasion boundary satisfies $\kappa_Z < 1/\sqrt{2}$. However, not all trade-offs will yield evolutionary branchings. We make the assumption that the trade-off curve lies entirely below the invasion boundary if $\kappa_T < \kappa_Z$. In this case there will be no evolutionary branching and only a single generalist consumer can persist evolutionarily in the system.

The case that remains to be determined is when $\kappa_Z < \kappa_T$ and $\kappa_A < \kappa_T$ (whether $\kappa_Z < \kappa_A$ or vice versa does not matter). When $\kappa_Z < \kappa_T$, the evolutionarily singular point $(a_1, a_2) = (1, 1)$ will be an evolutionary repellor (de Mazancourt & Dieckmann 2004), implying that any small perturbation away from the point will cause the consumer to evolve even further away from it. There are two types of possible endpoints of this directional evolution. In the first case, evolution continues until either a_1 or a_2 is zero. This leaves one of the resources unused and, due to symmetry, a mutant where the other affinity is zero can invade the system. Eventually, these two consumers will reach evolutionarily stable coexistence, each being specialized on an exclusive resource.

In the second case, evolution stops at some new evolutionarily singular point away from $(a_1, a_2) = (1, 1)$, where a_1 and a_2 are both nonzero. In the remainder of this section we will show that, if the generalist ZNGI of the consumers lies entirely below the critical generalist ZNGI, it is possible for a mutant to invade such a resident if the mutant's affinities are a mirror image of the resident's affinities, and that this results in a protected bimorphism that ensures evolutionary coexistence. This amounts to

a scenario where the resident drives the resources to an equilibrium point (R_1^*, R_2^*) that can be invaded by a mutant with exactly reversed affinities compared to the resident. Due to symmetry, switching the roles of mutant and resident would also have led to invasion, and hence mutual invasibility ensures that coexistence is possible. The proof is rather technical and requires both algebraic and graphical arguments.

Assume that $(a_1, a_2) = (1, 1)$ is an evolutionary repellor for a single consumer, leading to a new evolutionarily singular point at (a_1^*, a_2^*), with $a_1^* > a_2^*$. The case with $a_1^* < a_2^*$ will be the same due to symmetry. At this point, the system is in ecological equilibrium, with resource densities R_1^*, and R_2^*, and consumer density u^*, obeying the equations

$$0 = G(a_1^* R_1^*, a_2^* R_2^*) - \mu \tag{S1.20a}$$

$$0 = 1 - R_1^* - C_1(a_1^* R_1^*, a_2^* R_2^*)Gu^* \tag{S1.20b}$$

$$0 = 1 - R_2^* - C_2(a_1^* R_1^*, a_2^* R_2^*)Gu^* \,. \tag{S1.20c}$$

Recall our assumptions that $C_1 + C_2 \equiv 1$, that $a_1 R_1 > a_2 R_2 \iff C_1 > C_2$, and that $a_1 R_1 = a_2 R_2 \iff C_1 = C_2 = 1/2$. Given these premises we will show that

$$a_1^* > a_2^* \implies \begin{cases} a_1^* R_1^* & > a_2^* R_2^* \\ R_2^* & > R_1^* \,. \end{cases} \tag{S1.21}$$

These conditions are a prerequisite for our graphical argumentation further down. We will prove Eq. S1.21 by showing that any other combination, where one or more of the above inequalities is reversed, is impossible, case by case.

<u>Case 1:</u> $a_1^* > a_2^*$ and $a_1^* R_1^* < a_2^* R_2^*$.

Since $a_1^* R_1^* < a_2^* R_2^*$ it follows that $a_1^*/a_2^* R_1^* < R_2^* \implies R_2^* > R_1^*$. From our assumptions we also know that $C_2 > C_1$. Since we are in equilibrium, it must be true that

$$1 - R_1^* - C_1 G u^* = 0 \,. \tag{S1.22}$$

However, by the above inequalities this implies that

$$1 - R_2^* - C_2 G u^* < 0 \,, \tag{S1.23}$$

and so the system cannot be in equilibrium. This excludes case 1.

<u>Case 2:</u> $a_1^* > a_2^*$ and $a_1^* R_1^* = a_2^* R_2^*$.

Under these circumstances we know that $C_1 = C_2 = 1/2$, and we get that $R_2^* = a_1^*/a_2^* R_1^*$. Since resource 1 is in equilibrium we have:

$$1 - R_1^* - \frac{1}{2} G u^* = 0 \,. \tag{S1.24}$$

However, this implies that

$$1 - R_2^* - \frac{1}{2} G u^* = 1 - \frac{a_1}{a_2} R_1^* - \frac{1}{2} G u^* < 0 \,, \tag{S1.25}$$

since $a_1 > a_2$. This excludes case 2.

<u>Case 3:</u> $a_1^* > a_2^*$ and $a_1^* R_1^* > a_2^* R_2^*$.

Here $C_1 > C_2$, and the system

$$\begin{cases} 1 - R_1^* - C_1 G u^* &= 0 \\ 1 - R_2^* - C_2 G u^* &= 0 \end{cases} \tag{S1.26}$$

can be fulfilled, if and only if $R_1^* < R_2^*$. This proves Eq. S1.21.

Once the system described by Eqs. S1.20 has reached an evolutionarily singular point at (a_1^*, a_2^*), it will have driven the system to a resource equilibrium (R_1^*, R_2^*), which will be a point somewhere on the resident's ZNGI (blue line in Fig. S1.2A). We now ask ourselves whether a rare mutant with some given affinity combination on the trade-off curve will be able to invade the resident. Specifically, consider a mutant whose affinities a_1' and a_2' are the mirror images of the resident's affinities, so that $a_1' = a_2^*$ and $a_2' = a_1^*$ (Fig. S1.2B). For this given affinity combination, the mutant also has a ZNGI (dashed green line in Fig. S1.2A). If (R_1^*, R_2^*) is a point above the mutant's ZNGI, then the mutant will be able to grow in the environment set by the resident, and thus be able to invade. So far, however, this is merely an illustration, and in order to show that an invader will always be successful given the premises detailed above, we need to make a somewhat roundabout argument.

Consider the curves in Fig. S1.2. Here, we see the resident's ZNGI (blue line in Fig. S1.2A) and the trade-off curve (orange line in Fig. S1.2B), both mapped into the scaled space with axes $X = a_1 R_1$, and $Y = a_2 R_2$ (blue and orange lines in Fig. S1.2C). The resident's ZNGI is scaled by a_1^* and a_2^*, and the trade-off is scaled by R_1^* and R_2^*. This maps (a_1^*, a_2^*) in Fig. S1.2B and (R_1^*, R_2^*) in Fig. S1.2A onto the same point in scaled space, $(X^*, Y^*) = (a_1^* R_1^*, a_2^* R_2^*)$ in Fig. S1.2C. As proved above it must hold that $X^* > Y^*$ (since $a_1^* > a_2^*$, Eq. S1.21), so the point (X^*, Y^*) is below the diagonal in scaled space. Recall that the ZNGI in scaled space is the same for all consumers, as the per capita growth rate of all consumers is governed by the same function $G(X, Y)$.

We now consider whether a mutant with $a_1' = a_2^*$ and $a_2' = a_1^*$ is able to invade the system. We know that the point (a_1', a_2') is on the trade-off curve, due to the symmetry of the trade-off (Fig. S1.2B). For invasion to be possible, the net growth rate of the mutant must be positive, i.e., $G(a_1' R_1^*, a_2' R_2^*) - \mu > 0$. This is equivalent to the statement that the point (a_1', a_2') in affinity space, when mapped into scaled space to the point $(a_1' R_1^*, a_2' R_2^*)$ must lie above the ZNGI in scaled space (Fig. S1.2C). We will now show that this must be so.

Since it must be true that $X^* Y^* = a_1^* R_1^* a_2^* R_2^* = a_1' R_1^* a_2' R_2^*$ we can conclude that the point $(a_1' R_1^*, a_2' R_2^*)$ must lie on a curve described by $XY = X^* Y^*$ (black line in Fig. S1.2C). This curve is symmetrical about the diagonal in scaled space. Figure S1.2D shows a zoomed in and rotated part of Fig. S1.2C. We have assumed that the ZNGI in scaled space lies entirely below the critical generalist ZNGI (dashed line in Fig. S1.2D). Hence, both (X^*, Y^*) and the curve $XY = X^* Y^*$ lie below the critical generalist ZNGI as well. We can then conclude that the curve $XY = X^* Y^*$ must intersect the ZNGI at two points that are symmetrical about the diagonal, (X^*, Y^*) and (Y^*, X^*), and that the curve $XY = X^* Y^*$ lies below the ZNGI between these points and above the ZNGI outside of this interval. Hence, the curve $XY = X^* Y^*$ lies above the ZNGI to the left of the point (Y^*, X^*) (Fig. S1.2D). Thus, in order to show that the mutant with affinities (a_1', a_2') has positive invasion fitness, it suffices to show that the point $(a_1' R_1^*, a_2' R_2^*)$ lies to the left of $(Y^*, X^*) = (a_2^* R_2^*, a_1^* R_1^*)$ on the curve $XY = X^* Y^*$. This will be fulfilled if $a_1' R_1^* < a_2^* R_2^*$. Recall that $a_1^* > a_2^*$, that $a_1' = a_2^*$ and $a_2' = a_1^*$, and that $R_2^* > R_1^*$ by Eq. S1.21. Using these relations we can calculate

$$R_1^* < R_2^* \implies a_2^* R_1^* < a_2^* R_2^* \implies a_1' R_1^* < a_2^* R_2^* \,. \tag{S1.27}$$

Hence the point $(a_1' R_1^*, a_2' R_2^*)$ does indeed lie to the left of the point $(Y^*, X^*) = (a_2^* R_2^*, a_1^* R_1^*)$ on the curve $XY = X^* Y^*$, and thus also above the ZNGI (Fig. S1.2C).

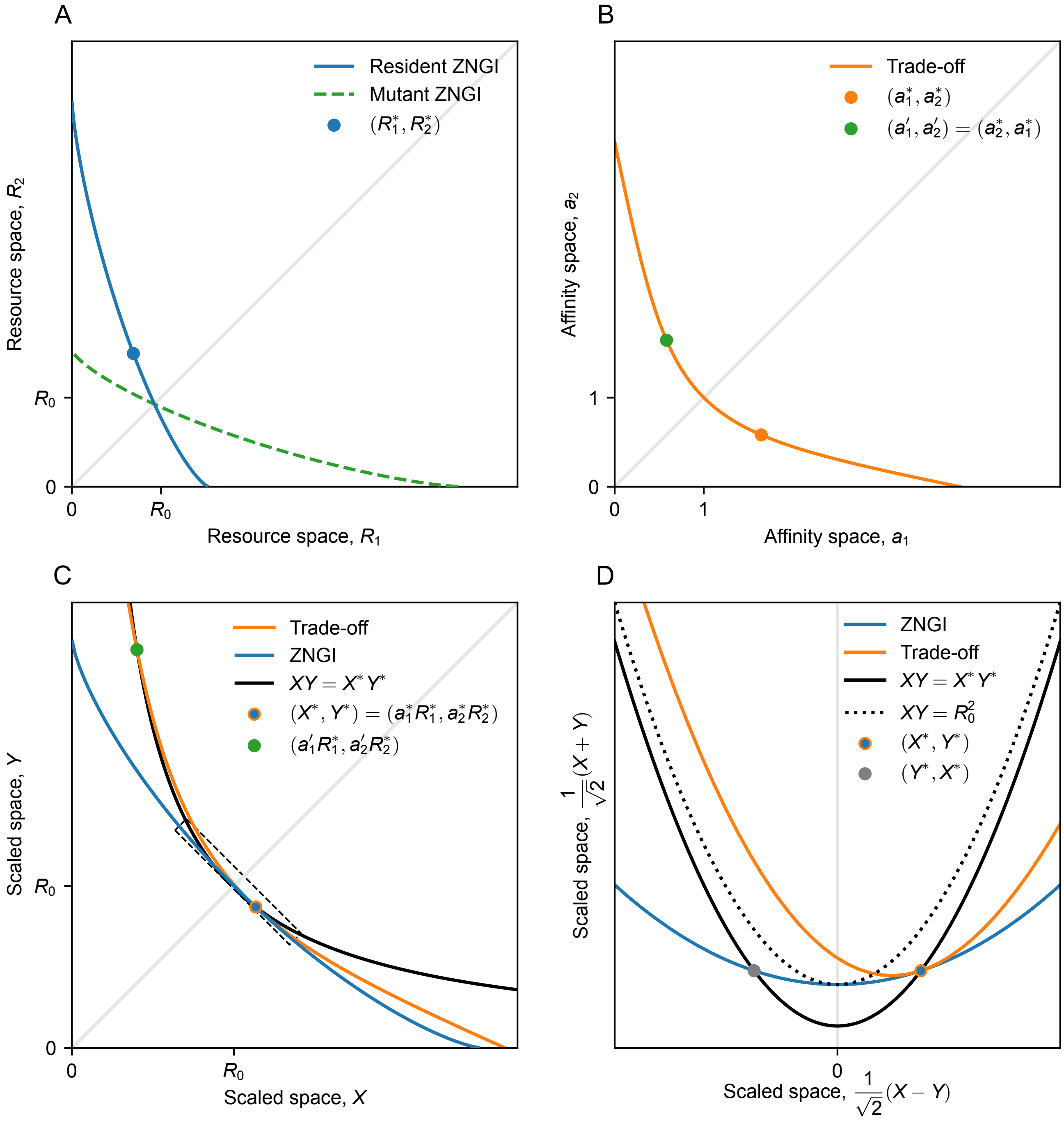

Figure S1.2: **A**: Zero net growth isocline (ZNGI) of a resident consumer plotted in resources space. Also shown are the equilibrium resource densities (R_1^*, R_2^*) in a system with only the resident, and the ZNGI of an invading mutant with affinities that are opposite to the resident's affinities. **B**: Trade-off curve, the evolutionarily singular point (a_1^*, a_2^*), and the affinity combination of the invading mutant $(a_1', a_2') = (a_2^*, a_1^*)$. **C**: The points and curves in A and B mapped into scaled space $(X, Y) = (a_1R_1, a_2R_2)$, where the quantities in A have been scaled by a_1^* and a_2^*, and the quantities in B have been scaled by R_1^* and R_2^*. **D**: Rotated zoom-in of C, where the area depicted is indicated by the thin dashed box in C, with the critical generalist ZNGI added as a dotted line. We do not draw the critical generalist ZNGI in panel C to avoid visual clutter. In all panels the gray line indicates where $x = y$, for all axis variables. The figure is drawn based on a numerical simulation, as described in Appendix S2.1.1, with $s = 0.75$, $a_0 = 5$, and other parameters as in Table S2.1.

What this means is that a mutant with $a'_1 = a^*_2$ and $a'_2 = a^*_1$ will always be able to invade in this scenario, and due to the symmetry the reverse condition will also be true. This implies mutual invasibility and a protected bimorphism, as long as the generalist ZNGI lies entirely below the curve $R_1 R_2 = R_0^2$. After the mutant and resident have achieved a new ecological equilibrium, they may still have to evolve directionally to achieve evolutionarily stable coexistence.

It is worth noting that, just as we have assumed ecological equilibrium dynamics, so, too, have we assumed evolutionary equilibrium dynamics. In principle, our arguments above cannot rule out evolutionary cycling, where the eco-evolutionary system oscillates between one and two consumers. However, under the assumption that no such cycles will occur, the arguments presented in this and the previous section suffice to prove that two consumers can coexist with evolutionary stability for sufficiently substitutable resources, and sufficiently specialist-favoring trade-offs.

S1.1.3 Homogeneous environments: Ecological coexistence is only possible if the generalist ZNGI lies below the critical generalist ZNGI

A necessary prerequisite for evolutionarily stable coexistence is stable ecological coexistence. Stable ecological coexistence of two resource competitors is only possible if, at equilibrium, each competitor is relatively more limited by a different resource, and if each competitor consumes relatively more of its more limiting resource (Tilman 1980). In the following, we will show that this condition is only fulfilled when the generalist ZNGI lies entirely below the critical generalist ZNGI. Conversely, if the generalist ZNGI of the consumers lies entirely above the critical generalist ZNGI, stable ecological coexistence—and hence evolutionarily stable coexistence—is impossible. We will only prove in detail the case for which coexistence is impossible, as the converse case follows from a fully analogous argument.

Consider an equilibrium system with two coexisting consumers:

$$0 = G^1(a_1^1 R_1^*, a_2^1 R_2^*) - \mu \tag{S1.28a}$$

$$0 = G^2(a_1^2 R_1^*, a_2^2 R_2^*) - \mu \tag{S1.28b}$$

$$0 = 1 - R_1^* - C_1^1(a_1^1 R_1^*, a_2^1 R_2^*) G^1 u^{1*} - C_1^2(a_1^2 R_1^*, a_2^2 R_2^*) G^2 u^{2*} \tag{S1.28c}$$

$$0 = 1 - R_2^* - C_2^1(a_1^1 R_1^*, a_2^1 R_2^*) G^1 u^{1*} - C_2^2(a_1^2 R_1^*, a_2^2 R_2^*) G^2 u^{2*}\,. \tag{S1.28d}$$

Here, superscripts pertain to consumers, subscripts to resources, and asterisks indicate equilibrium densities of the resources and consumers. We note, again, that G^1 and G^2 are identical functions, but with different arguments, due to the differing affinities of the consumers. We assume that the affinities of the consumers are such that consumer 1 consumes relatively more of resource 1 than does consumer 2, i.e., $C_1^1/C_2^1 > C_1^2/C_2^2$, where C_1^i/C_2^i corresponds to the slope of the consumption vector of consumer i (thin arrows in Fig. S1.3A). For stable coexistence to be possible, consumer 1 must then be relatively more limited by resource 1 than is consumer 2, i.e., it must hold that

$$\frac{\partial G^1}{\partial R_1} \bigg/ \frac{\partial G^1}{\partial R_2} > \frac{\partial G^2}{\partial R_1} \bigg/ \frac{\partial G^2}{\partial R_2}\,, \tag{S1.29}$$

where the derivatives are evaluated at the equilibrium (R_1^*, R_2^*). Graphically, $(\partial G^i/\partial R_2)/(\partial G^i/\partial R_1)$ is the slope of the normal vector (i.e., a vector perpendicular to the tangent) of the ZNGI of consumer i at the equilibrium point, which points relatively more in the direction of resource 1 if resource 1 is more limiting (illustrated by the thick red arrow belonging to ZNGI 2 in Fig. S1.3A), and relatively more in the direction of resource 2 if resource 2 is more limiting (thick blue arrow belonging to ZNGI 1, Fig. S1.3A). Thus, if consumer 1 consumes proportionally more of resource 1 than consumer 2, then the normal vector

of the ZNGI for consumer 1, evaluated at the equilibrium, must point relatively more in the direction of resource 1 compared to consumer 2, in order for stable coexistence to be possible (note that the example in Fig. S1.3A illustrates the opposite case where coexistence is impossible).

Stated differently, if consumer 1 consumes proportionally more of resource 1 than consumer 2, then stable coexistence is only possible if the ZNGI of consumer 1 intersects the ZNGI of consumer 2 from above. In the following, we will show that this condition is only fulfilled when the generalist ZNGI lies entirely below the critical generalist ZNGI. We cannot prove this directly. Instead, we will introduce a third line $R_1R_2 = R_1^*R_2^*$ (gray line in Fig. S1.3A), which we can show is intersected from above by one of the ZNGIs, and from below by the other ZNGI.

As in the previous section, we introduce two new scaled variables, $X = a_1R_1$ and $Y = a_2R_2$. Furthermore, let $X^1 = a_1^1R_1^*$, $Y^1 = a_2^1R_2^*$, $X^2 = a_1^2R_1^*$, and $Y^2 = a_2^2R_2^*$. In scaled X–Y space, both consumers have the same scaled ZNGI given by $G(X, Y) - \mu = 0$ (Fig. S1.3B), which is identical in shape to their generalist ZNGI (see Fig. 3 in main text). We note that our assumptions regarding the proportions of consumption imply that

$$\frac{C_1^1}{C_2^1} > \frac{C_1^2}{C_2^2} \iff \frac{X^1}{Y^1} > \frac{X^2}{Y^2}\,. \tag{S1.30}$$

Hence, the point (X^1, Y^1) must lie to the right of the point (X^2, Y^2) on the scaled ZNGI (Fig. S1.3B). We now draw two additional curves in scaled space, $XY = X^1Y^1$, which goes through the point (X^1, Y^1), and $XY = X^2Y^2$, which goes through the point (X^2, Y^2) (Fig. S1.3B). Note that the scaled ZNGI of the consumers is tangent to the critical generalist ZNGI $XY = R_0^2$ in the point (R_0, R_0), but lies everywhere else either entirely above or entirely below the critical ZNGI. In contrast, the curves $XY = X^1Y^1$ and $XY = X^2Y^2$ do not touch or intersect the critical generalist ZNGI $XY = R_0^2$.

In the following, we assume that the scaled (= generalist) ZNGI lies above the critical generalist ZNGI. In this case, the two curves $XY = X^1Y^1$ and $XY = X^2Y^2$ must intersect the scaled ZNGI twice and in such a way that $XY = X^2Y^2$ crosses the ZNGI from below at the point (X^2, Y^2) and $XY = X^1Y^1$ crosses the ZNGI from above at the point (X^1, Y^1) (Fig. S1.3B). We can then go back from scaled space to resource space by rescaling the scaled ZNGI of consumer 1 and the curve $XY = X^1Y^1$ by $1/a_1^1$ and $1/a_2^1$ in the R_1 and R_2 directions, respectively, and by rescaling the scaled ZNGI of consumer 2 and the curve $XY = X^2Y^2$ by $1/a_1^2$ and $1/a_2^2$ in the R_1 and R_2 directions. Rescaling the curves in Fig. S1.3B into resource space will collapse the curves $XY = X^1Y^1$ and $XY = X^2Y^2$ onto a single curve described by $R_1R_2 = R_1^*R_2^*$, whereas the scaled ZNGIs will become separate ZNGIs for consumer 1 and 2, respectively (Fig. S1.3A). Since all we have done is rescale the coordinates, the order of slopes is preserved. Thus, since the scaled ZNGI crosses the curve $XY = X^1Y^1$ from below in the point (X^1, Y^1) (Fig. S1.3B), so will the ZNGI for consumer 1 cross the curve $R_1R_2 = R_1^*R_2^*$ from below in resource space at the point (R_1^*, R_2^*) (Fig. S1.3A). By the same argument, the ZNGI for consumer 2 will cross the curve $R_1R_2 = R_1^*R_2^*$—and thus also the ZNGI of consumer 1—from above in resource space. Thus, at equilibrium, the normal of the ZNGI of consumer 1 will point relatively more in the R_2-direction compared to the normal of consumer 2, which is opposite to the order of the slopes of the consumption vectors of the two consumers (Fig. S1.3A). Hence, coexistence is not possible if the scaled (= generalist) ZNGI lies above the critical generalist ZNGI $R_1R_2 = R_0^2$ (Fig. S1.3B). By an analogous argument, one can show that equilibria for generalist ZNGIs lying below the critical generalist ZNGI will yield stable equilibria.

In our argument (and in Fig. S1.3) we have for ease of illustration picked a case where $a_1^1 > a_2^1$ and $a_2^2 > a_1^2$. Since a constant scaling of coordinates will preserve the order of angles between curves, the argument holds even without this provision.

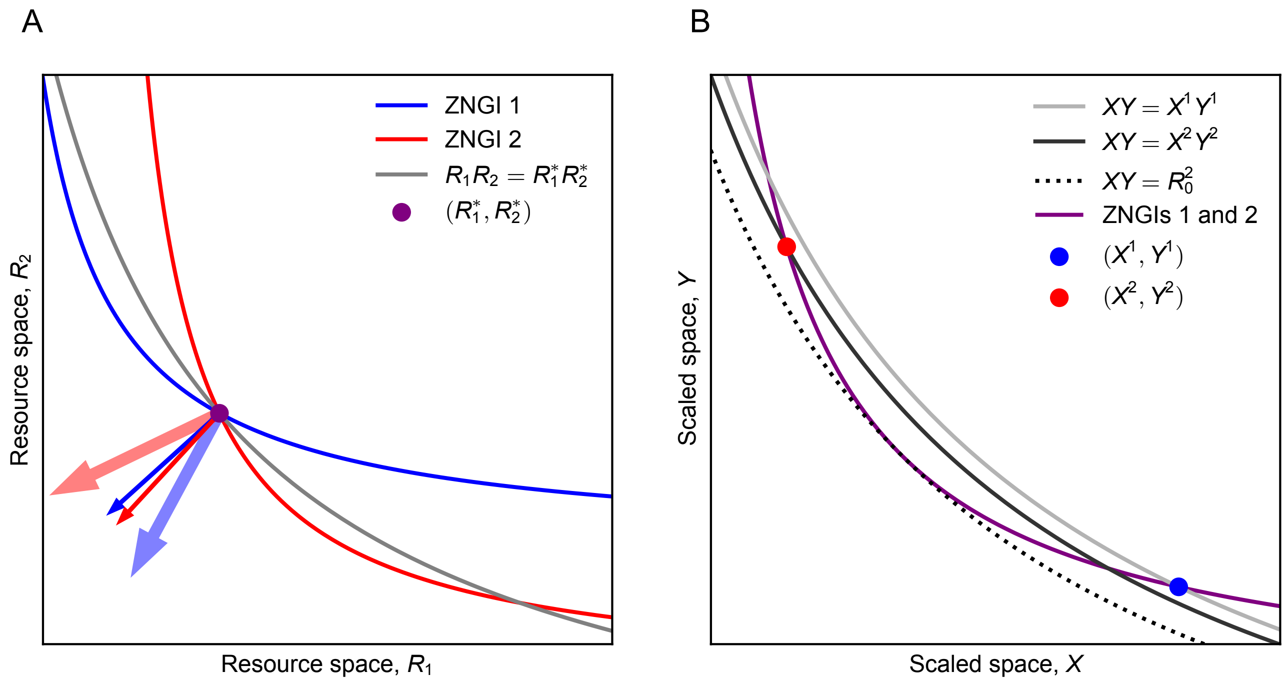

Figure S1.3: **A**: Zero net growth isoclines (ZNGIs) for two consumers that cannot coexist stably, the equilibrium resource densities (R_1^*, R_2^*), and the curve $R_1R_2 = R_1^*R_2^*$ in resource space. Thick arrows are the normal vectors of the ZNGIs, and thin arrows are the consumption vectors of the consumers, where the lengths of the vectors have been normalized. **B**: The curves and points in A, mapped into scaled space $(X, Y) = (a_1R_1, a_2R_2)$. The ZNGIs in A are mapped onto the same ZNGI in scaled space, by scaling ZNGI 1 by a_1^1 and a_2^1, and ZNGI 2 by a_1^2 and a_2^2. The point (R_1^*, R_2^*) in A is mapped onto the points $(X_1, Y_1) = (a_1^1R_1^*, a_2^1R_2^*)$ and $(X_2, Y_2) = (a_1^2R_1^*, a_2^2R_2^*)$. The curve $R_1R_2 = R_1^*R_2^*$ is mapped onto the curves $XY = X_1Y_1$ and $XY = X_2Y_2$. Note that to clearly show how lines intersect, the origin is not included in the scope of either panel. The figure is drawn based on a numerical simulation, as described in AppendixS2.1.1, with $s = -1$, $a_0 = 10$, and other parameters as in Table S2.1. The traits for the two consumers are $\chi_1 = -0.2$ and $\chi_2 = 0.1$.

S1.2 Heterogeneous environments

S1.2.1 Heterogeneous environments: Why diversity is low near the critical generalist ZNGI

Here, we consider why we only get one or two coexisting consumers near the critical generalist ZNGI with curvature $\kappa_Z = 1/\sqrt{2}$ (corresponding to $\hat{\kappa}_Z = 1$ in Fig. 6 of the main text). More specifically, we consider why, if the generalist ZNGI of the net growth function is given by the critical generalist ZNGI $R_1R_2 = R_0^2$, we do not get more coexisting consumers. For the critical generalist ZNGI, we know that $G(1 \cdot R_1, 1 \cdot R_2) - \mu = 0$ has the solution $R_1R_2 = R_0^2$, which results in that $G(X, Y) - \mu = 0$ will have the solution $XY = R_0^2$. If we now assume that the resources are of the same type for all resource densities (i.e., the resources will not, for example, change from substitutable to essential as resource densities increase), then all level curves of the function $G(X, Y) - \mu$ will satisfy $XY = c$ for some constant c that depends on which level curve we are considering. If so, then the function $G(X, Y)$ must be expressible as a function of only XY, so that $G(X, Y) = f(XY)$. For example, the per capita growth function used in our numerical implementation for heterogeneous environments is given by

$$G(X, Y) = \frac{1}{\frac{1}{G_{\max}} + \frac{1}{\sqrt{XY}}}, \tag{S1.31}$$

when $\kappa_Z = 1/\sqrt{2}$, which is indeed only a function of the product of X and Y (see Eq. S2.2 and the subsequent paragraph in Appendix S2.1.1 for additional details concerning the specification of the model used in our numerical investigations).

In order for the consumers to be at an eco-evolutinoary equilibrium, the selection gradient for all consumers must be zero. We will now show that, when $\kappa_Z = 1/\sqrt{2}$ only one or two consumers will be able to coexist by showing that the selection gradient for residents can be zero for maximally two consumers. By Wickman *et al.* (2017), the selection gradient $\mathcal{D}(\chi_{\text{res}})$ acting on a trait value χ_{res} for a resident with equilibrium density $u_{\text{res}}(\mathbf{x})$ in a reaction-diffusion system is given by

$$\mathcal{D}(\chi_{\text{res}}) = \frac{1}{\int u_{\text{res}}^2 \mathrm{d}\mathbf{x}} \int \left. \frac{\partial G(a_1(\chi)R_1, a_2(\chi)R_2)}{\partial \chi} \right|_{\chi=\chi_{\text{res}}} u_{\text{res}}^2 \mathrm{d}\mathbf{x}. \tag{S1.32}$$

Now, since $G(X,Y) = f(XY)$, we can calculate

$$\left. \frac{\partial G(a_1(\chi)R_1, a_2(\chi)R_2)}{\partial \chi} \right|_{\chi=\chi_{\text{res}}} = \left. \frac{\partial G(X,Y)}{\partial \chi} \right|_{\chi=\chi_{\text{res}}} = \left. \frac{\partial f(XY)}{\partial \chi} \right|_{\chi=\chi_{\text{res}}} =$$
$$= \left[\frac{\partial f(XY)}{\partial (XY)} \frac{\partial (XY)}{\partial \chi} \right]_{\chi=\chi_{\text{res}}} = \left[\frac{\partial f(XY)}{\partial (XY)} R_1 R_2 \frac{\mathrm{d}(a_1(\chi)a_2(\chi))}{\mathrm{d}\chi} \right]_{\chi=\chi_{\text{res}}}. \tag{S1.33}$$

In the expression above, $\partial f(XY)/\partial(XY)$ will be positive, as we assume that the per capita growth function is increasing when resource availability increases, and R_1 and R_2 must be positive since they are resource densities. Hence, the sign of the above expression is determined by the factor $\mathrm{d}(a_1(\chi)a_2(\chi))/\mathrm{d}\chi$. Since the affinities $a_j(\chi)$ of a given phenotype are the same everywhere in space, it follows that $\partial G(a_1(\chi)R_1, a_2(\chi)R_2)/\partial\chi|_{\chi=\chi_{\text{res}}}$ is of one sign everywhere in space, and so the only way for the selection gradient (Eq. S1.32) to be zero is if the integrand is everywhere zero, i.e., if $\mathrm{d}(a_1(\chi)a_2(\chi))/\mathrm{d}\chi$ is zero. When the trade-off is generalist-favoring or mildly specialist-favoring, $a_1(\chi)a_2(\chi)$ has a single maximum at the generalist trait combination $a_1 = a_2 = 1$. When the trade-off is sufficiently specialist-favoring, $a_1(\chi)a_2(\chi)$ can have two maxima at some trait combination $a_1 = a_f > 1$ and $0 < a_2 = a_g < 1$ and its mirror image $a_1 = a_g$ and $a_2 = a_f$, separated by a local minimum at $a_1 = a_2 = 1$. Thus, near the critical generalist ZNGI, the evolutionarily stable equilibrium will consist of either a single generalist consumer or of two consumers that are relatively more specialized on opposite resources, depending on the shape of the trade-off.

In ecological terms this means that, in parameter space near the critical generalist ZNGI, we can distinguish two cases. (1) If the trade-off curve is linear to generalist favoring ($\hat{\kappa}_T \leq 0$ in Fig. 6A), $a_1(\chi)a_2(\chi)$ has a single maximum at the trait combination $a_1 = a_2 = 1$. This means that the ZNGI of a perfect generalist is below the ZNGIs of all other trait combinations on the trade-off curve. Consequently, the generalist will locally outcompete all other trait combinations everywhere in space, regardless of how skewed the local resource-supply ratios may be. For this case, the generalist–specialist dichotomy therefore loses its meaning, because 'specialists' cannot depress their favored resource to lower levels than a perfect generalist. In fact, on the critical generalist ZNGI and for linear or generalist-favoring trade-offs, competitive ability depends exclusively on the product $a_1(\chi)a_2(\chi)$ (which is maximized for $a_1 = a_2 = 1$), and two consumers that are 'specialized' on opposite resources ($a_{i1} > a_{i2}$ and $a_{j1} < a_{j2}$) but have identical $a_{i1}a_{i2} = a_{j1}a_{j2}$ also have identical ZNGIs. (2) Alternatively, if the trade-off is sufficiently specialist-favoring ($\hat{\kappa}_T > 0$ in Fig. 6A), $a_1(\chi)a_2(\chi)$ can have two maxima, in which case two (relative) specialists with opposite trait combinations will outcompete all other trait combinations.

These two cases can also be understood in terms of the graphical niche theory of geometrical envelopes developed by Koffel *et al.* (2016). This theory uses the outer geometrical envelope of the ZNGIs of all possible phenotypes/trait combinations—which, in our model, are all trait combinations (a_1, a_2) on a given

trade-off curve—to graphically derive the trait combination(s) that competitively exclude all other trait combinations at any given resource supply point (K_1, K_2). In the case of resources of critical essentiality (i.e. resource types described by the critical generalist ZNGI), this outer geometrical envelope coincides with the critical generalist ZNGI when the trade-off is neutral to generalist-favoring (case 1 in the previous paragraph). The latter implies that the ZNGIs of all other phenotypes ($a_1 \neq a_2$) are nested inside (= above) the outer geometrical envelope represented by the critical generalist ZNGI $R_1R_2 = R_0^2$, and the generalist will locally outcompete all other trait combinations as long as the local resource supply point (K_1, K_2) is above the envelope. In contrast, when the trade-off is sufficiently specialist-favoring (case 2 in the previous paragraph), the outer geometrical envelope consists of the outer parts of the intersecting ZNGIs of two (relative) specialists with exactly opposite trait combinations (a_1, a_2). A combination of the upper left part of the solid ZNGI and the lower right part of the broken ZNGI in Fig. 5C of the main text can serve as an illustration of the general appearance of such a bisectional outer geometrical envelope. (Note, however, that the example of Fig. 5C is not located on the critical generalist ZNGI). In a spatially heterogeneous landscape, the two relative specialists will outcompete all other phenotypes, with the relative R_1-specialist dominating where the local supply K_1 is low, and the relative R_2-specialist dominating where the local supply K_2 is low.

S1.2.2 Heterogeneous environments: Correlation patterns of resource specialists with local resource supply

We state in the main text that, when resources are functionally substitutable ($\hat{\kappa}_Z < 1$ in Fig. 6A in the main text), consumers tend to be aligned with the local supply of their favored resource; i.e. resource specialists inhabit primarily those parts of the landscape where their favored resource has a high supply. Conversely, when resources are functionally essential ($\hat{\kappa}_Z > 1$), resource specialists tend to primarily inhabit parts of the landscape where their favored resource has low supply. While we cannot show this analytically, we can present supporting evidence from our numerical simulations in greater detail.

Let the resource supplies for the system be given by $K_1(\mathbf{x})$ and $K_2(\mathbf{x})$. For each consumer we calculate a measure of the experienced total supply of each resource as

$$K_{\mathrm{E},1} = \frac{1}{\int u(\mathbf{x})\mathrm{d}\mathbf{x}} \int K_1(\mathbf{x})u(\mathbf{x})\mathrm{d}\mathbf{x} \tag{S1.34a}$$

$$K_{\mathrm{E},2} = \frac{1}{\int u(\mathbf{x})\mathrm{d}\mathbf{x}} \int K_2(\mathbf{x})u(\mathbf{x})\mathrm{d}\mathbf{x}\,. \tag{S1.34b}$$

If local consumer density $u(\mathbf{x})$ is closely aligned with the local supply of resource j across the landscape, then $K_{\mathrm{E},j}$ will be large. Conversely, if local consumer density is inversely related to the local supply of resource j, then $K_{\mathrm{E},j}$ will be small. Let a_1 and a_2 be the resource affinities of the consumer. We now calculate a measure of how aligned the consumer is with the local supply of its favored resource (i.e., the resource for which it has the higher affinity) as

$$\begin{cases} A_{\mathrm{fav}} = \log \dfrac{K_{E,1}}{K_{E,2}}, & \text{if } a_1 > a_2 \\ A_{\mathrm{fav}} = \log \dfrac{K_{E,2}}{K_{E,1}}, & \text{if } a_2 > a_1\,. \end{cases} \tag{S1.35}$$

We calculated this favored resource alignment for all consumers across all communities from the numerical example in Fig. 6A of the main text, and depict the results in Fig. S1.4. There, we can clearly see that, for functionally substitutable resources ($\hat{\kappa}_Z < 1$), consumers are aligned with the local supply of their favored resource ($A_{\mathrm{fav}} > 0$), whereas for functionally essential resources ($\hat{\kappa}_Z > 1$), consumers are counter-aligned with the local supply of their favored resource ($A_{\mathrm{fav}} < 0$).

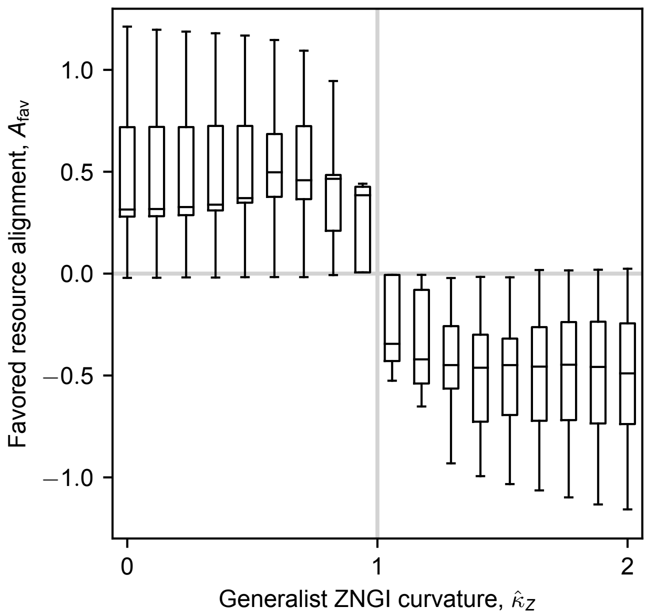

Figure S1.4: Alignment of consumers with their favored resource, A_{fav}, in the communities in Fig. 6A of the main text, binned by generalist zero net growth isocline (ZNGI) curvature $\hat{\kappa}_{\text{Z}}$. A_{fav} was calculated separately for all consumers in all communities. The boxplots show medians, quartiles, and whiskers covering the maximal and minimal favored resource alignments.

This pattern can, again, be understood in terms of the graphical niche theory of outer geometrical envelopes developed by Koffel *et al.* (2016), see section S1.2.1. When resources are sufficiently essential ($\hat{\kappa}_{\text{Z}} > 1$ in Fig. 6A), the ZNGIs of many different trait combinations can be tangent to different parts of the outer geometrical envelope (e.g., Figs. 6B and C of the main text). This means that different trait combinations can outcompete all other trait combinations at localities in space where the local resource supply ratio K_1/K_2 is inversely ranked to their affinity ratio a_1/a_2. Consequently, consumers are counter-aligned with the local supply of their favored resource when resources are essential (Figs. 6B and C of the main text). When resources are sufficiently substitutable ($\hat{\kappa}_{\text{Z}} < 1$ in Fig. 6A), it is also possible that the ZNGIs of several different trait combinations are tangent to different parts of the outer geometrical envelope, but only when the trade-off curvature fulfills $\hat{\kappa}_{\text{T}} < \hat{\kappa}_{\text{Z}}$ (e.g. Fig. 6E of the main text). When the reverse reverse is true ($\hat{\kappa}_{\text{T}} > \hat{\kappa}_{\text{Z}}$), i.e., when the trade-off is sufficiently specialist-favoring, the outer geometrical envelope will be made up by the ZNGIs of the two extreme specialists (e.g. Fig. 6F of the main text), and these two specialists will, together, outcompete all other trait combinations everywhere on the landscape. Regardless of the relative ranking of $\hat{\kappa}_{\text{T}}$ and $\hat{\kappa}_{\text{Z}}$, substitutable resources enable consumers to become most abundant where the local supply of their favored resource is highest. Consequently, consumers are aligned with the local supply of their favored resource when resources are substitutable (Figs. 6E and F of the main text). Note that diffusive movement of resources and consumers blurs this picture, implying that local exclusion of phenotypes can strictly only occur when a phenotype is completely absent from the landscape.

References

Geritz, S.A.H., Kisdi, E., Meszéna, G. & Metz, J.A.J. (1998). Evolutionarily singular strategies and the adaptive growth and branching of the evolutionary tree. *Evol. Ecol.*, 12, 35–57.

Koffel, T., Daufresne, T., Massol, F. & Klausmeier, C.A. (2016). Geometrical envelopes: Extending

graphical contemporary niche theory to communities and eco-evolutionary dynamics. *J. Theor. Biol.*, 407, 271–289.

de Mazancourt, C. & Dieckmann, U. (2004). Trade-off geometries and frequency-dependent selection. *Am. Nat.*, 164, 765–778.

Tilman, D. (1980). Resources: A graphical-mechanistic approach to competition and predation. *Am. Nat.*, 116, 362–393.

Wickman, J., Diehl, S., Blasius, B., Klausmeier, C.A., Ryabov, A. & Brännström, Å. (2017). Determining selection across heterogeneous landscapes: a perturbation-based method and its application to modeling evolution in space. *Am. Nat.*, 189, 381–395.
