# Supplementary material for: Evolution of resource specialisation in competitive metacommunities
Source: Ecol Lett. 2019 Aug 7;22(11):1746–56. doi: 10.1111/ele.13338 (PMC6852178; doi:10.1111/ele.13338)
Supplement: Supplementary file 2 [file ELE-22-1746-s002.pdf]

Appendix S2: Specific implementation of the ecological model, numerical methods, and additional examples of functions describing resource competition for substitutable to essential resources

S2.1 Specific implementation of the ecological model

To confirm our analytical results concerning when evolutionarily stable coexistence is possible in homogeneous environments, and to obtain results for heterogeneous environments we implement specific families of net growth functions, proportions of consumption, and trade-offs, and perform numerical simulations of these equations. No existing mechanistic ecological model allows resources to be varied continuously from fully substitutable to fully essential. We therefore designed phenomenological consumption and growth functions which have this property, with growth functions similar to those used by Schreiber & Tobiason (2003). In this appendix, we first describe these functions and how we used them to numerically implement the ecological dynamics in spatially homogeneous and heterogeneous environments. In section S2.3, we describe how several of the most widely used ecological models fit the generic equations under more restricted conditions of resource substitutability.

S2.1.1 Homogeneous environments

For homogeneous environments, our generic equations (Eqs. 1 in the main text) are:

$$\frac{\mathrm{d}u_i(t)}{\mathrm{d}t} = G(a_{i1}R_1, a_{i2}R_2)u_i - \mu u_i \tag{S2.1a}$$

$$\frac{\mathrm{d}R_1(t)}{\mathrm{d}t} = r(K - R_1) - \sum_{i=1}^{N} C_1(a_{i1}R_1, a_{i2}R_2)G(a_{i1}R_1, a_{i2}R_2)u_i \tag{S2.1b}$$

$$\frac{\mathrm{d}R_2(t)}{\mathrm{d}t} = r(K - R_2) - \sum_{i=1}^{N} C_2(a_{i1}R_1, a_{i2}R_2)G(a_{i1}R_1, a_{i2}R_2)u_i\,. \tag{S2.1c}$$

In order to have a full eco-evolutionary model, we need to specify the family of net growth functions G describing uptake of resources, the family of functions describing the proportions of consumption C_1 and C_2, as well as the trade-off curves between the affinities a_{i1} and a_{i2}. We have chosen phenomenological formulations that allow resources to range continuously from substitutable to essential and trade-offs to range continuously from strongly generalist- to strongly specialist-favoring. All state variables and parameters are defined and listed with their units in Table S2.1.

We specify the net growth functions as

$$G(a_{i1}R_1, a_{i2}R_2) = \frac{1}{\frac{1}{G_{\max}} + \frac{1}{T(a_{i1}R_1, a_{i2}R_2)}}\,, \tag{S2.2a}$$

$$T(a_{i1}R_1, a_{i2}R_2) = 2^{-\frac{1}{s}}\left[(a_{i1}R_1)^s + (a_{i2}R_2)^s\right]^{\frac{1}{s}}\,, \tag{S2.2b}$$

where G saturates at a maximal growth rate $G_{\max}$, and the parameter $s \in (-\infty, 1]$ governs to what extent resources are substitutable. When $s = 1$, resources are perfectly substitutable, and the per capita growth function is equivalent to a type II functional response with two resources with attack rates a_1 and a_2 and handling times $1/G_{\max}$ for both resources. When $s \to -\infty$ resources are strictly essential, and the per capita growth function is equivalent to a saturating function where only $\min\{a_1R_1, a_2R_2\}$ determines

the growth of the consumer. The function T is not defined for $s = 0$, but its limit can be calculated to be $\lim_{s\to 0} T(a_{i1}R_1, a_{i2}R_2) = \sqrt{a_{i1}R_1 a_{i2}R_2}$. Since this is a function that depends only on the product of a_1R_1 and a_2R_2, the ZNGI of the net growth function will in this limit be given by the "critical generalist ZNGI", defined in Appendix S1.1 as $R_1R_2 = R_0^2$ when $a_1 = a_2 = 1$. The per capita growth function specified by Eqs. S2.2 is similar to that of Schreiber & Tobiason (2003), with the differences that our growth function is saturating and that it is normalized so that if $a_{i1}R_1 = a_{i2}R_2 = c$, where c is some constant, then $T = c$ for all s.

We specify the proportions of consumption as

$$C_1(a_{i1}R_1, a_{i2}R_2) = \frac{(a_{i1}R_1)^E}{(a_{i1}R_1)^E + (a_{i2}R_2)^E} \tag{S2.3a}$$

$$C_2(a_{i1}R_1, a_{i2}R_2) = \frac{(a_{i2}R_2)^E}{(a_{i1}R_1)^E + (a_{i2}R_2)^E}\,, \tag{S2.3b}$$

where $E = \exp(s-1)$. These functional forms imply that when resources are completely substitutable ($s = 1$), resources are consumed in proportion to their effective availability $a_{ij}R_j$ as would be the case for Lotka-Volterra dynamics. When resources are completely essential ($s \to -\infty$), resources are consumed in equal proportion so that $C_1 = C_2 = 1/2$.

To specify the trade-off curves, we introduce a dimensionless parameter $\chi_i \in [-1, 1]$ for each consumer of which both $a_{i1}(\chi_i)$ and $a_{i2}(\chi_i)$ are functions. These functions satisfy that $a_{i1}(1) = 0$, $a_{i2}(-1) = 0$, and $a_{i1}(0) = a_{i2}(0) = 1$. The precise functions are given by

$$\frac{a_{i1}}{a_{\max}} = 1 + \frac{1}{a_0 - b\log\left(\frac{1}{2}(\exp(-a_0)+1)\right)}\left(b\log\left(\frac{1}{2}(\exp(-a_0\chi_i)+1)\right) - a_0\chi_i\right) \tag{S2.4a}$$

$$\frac{a_{i2}}{a_{\max}} = 1 + \frac{1}{a_0 - b\log\left(\frac{1}{2}(\exp(-a_0)+1)\right)}\left(b\log\left(\frac{1}{2}(\exp(a_0\chi_i)+1)\right) + a_0\chi_i\right)\,. \tag{S2.4b}$$

Here, b is a parameter controlling how large $a_{i1}(\chi_i = -1)$ and $a_{i2}(\chi_i = 1)$ can get when trade-offs favor specialization. Because the right-hand side of Eqs. S2.4 is unitless, we introduce a parameter $a_{\max}$ with identical units as a_{ij} which we set to 1. a_0 is a parameter controlling to what degree the trade-off favors generalists or specialists. When $a_0 < 0$, the trade-off favors generalists, and when $a_0 > 0$, the trade-off favors specialists. Fig. 1 in the main text depicts trade-off curves, where $b = 4$, and $a_0 = -5, -5/4, 2, 10$ for panels E, F, G, and H.

We nondimensionalize Eqs. S2.1 by introducing the variables $\hat{t} = t(G_{\max} - \mu)$, $\hat{u}_i = u_i/K$, $\hat{R}_j = R_j/K$. This leads to a set of scaled equations

$$\frac{\mathrm{d}\hat{u}_i(\hat{t})}{\mathrm{d}\hat{t}} = \hat{G}(\hat{a}_{i1}\hat{R}_1, \hat{a}_{i2}\hat{R}_2)\hat{u}_i + \left(1 - \frac{1}{\alpha}\right)\hat{u}_i \tag{S2.5a}$$

$$\frac{\mathrm{d}\hat{R}_1(\hat{t})}{\mathrm{d}\hat{t}} = \gamma(1 - \hat{R}_1) - \sum_{i=1}^{N} C_1(\hat{a}_{i1}\hat{R}_1, \hat{a}_{i2}\hat{R}_2)\hat{G}(\hat{a}_{i1}\hat{R}_1, \hat{a}_{i2}\hat{R}_2)\hat{u}_i \tag{S2.5b}$$

$$\frac{\mathrm{d}\hat{R}_2(\hat{t})}{\mathrm{d}\hat{t}} = \gamma(1 - \hat{R}_2) - \sum_{i=1}^{N} C_2(\hat{a}_{i1}\hat{R}_1, \hat{a}_{i2}\hat{R}_2)\hat{G}(\hat{a}_{i1}\hat{R}_1, \hat{a}_{i2}\hat{R}_2)\hat{u}_i \tag{S2.5c}$$

$$\hat{G}(\hat{a}_{i1}\hat{R}_1, \hat{a}_{i2}\hat{R}_2) = \frac{1}{\alpha + \frac{\beta}{T(\hat{a}_{i1}\hat{R}_1, \hat{a}_{i2}\hat{R}_2)}}\,, \quad T(\hat{a}_{i1}\hat{R}_1, \hat{a}_{i2}\hat{R}_2) = 2^{-\frac{1}{s}}\left[(\hat{a}_{i1}\hat{R}_1)^s + (\hat{a}_{i2}\hat{R}_2)^s\right]^{\frac{1}{s}} \tag{S2.5d}$$

$$C_1(\widehat{a}_{i1}\widehat{R}_1, \widehat{a}_{i2}\widehat{R}_2) = \frac{(\widehat{a}_{i1}\widehat{R}_1)^E}{(\widehat{a}_{i1}\widehat{R}_1)^E + (\widehat{a}_{i2}\widehat{R}_2)^E}\,, \quad C_2(\widehat{a}_{i1}\widehat{R}_1, \widehat{a}_{i2}\widehat{R}_2) = \frac{(\widehat{a}_{i2}\widehat{R}_2)^E}{(\widehat{a}_{i1}\widehat{R}_1)^E + (\widehat{a}_{i2}\widehat{R}_2)^E}\,, \tag{S2.5e}$$

$$E = \exp(s-1) \tag{S2.5f}$$

$$\alpha = \frac{G_{\max} - \mu}{G_{\max}}\,, \quad \beta = \frac{G_{\max} - \mu}{a_{\max} K}\,, \quad \gamma = \frac{r}{G_{\max} - \mu} \tag{S2.5g}$$

$$\widehat{a}_{i1} = \frac{a_{i1}}{a_{\max}}\,, \quad \widehat{a}_{i2} = \frac{a_{i2}}{a_{\max}}\,. \tag{S2.5h}$$

All parameters of the nondimensionalized model (Eqs. S2.5) are defined and listed with their values in table S2.1. For these values, and $\widehat{a}_{i1} = \widehat{a}_{i2} = 1$, the generalist zero net growth isoclines (ZNGIs) for $\widehat{G} + 1 - \frac{1}{\alpha}$ are depicted in Fig. 1A-D in the main text, with $s = 1, 1/2, -1/4, -100$ for panels A, B, C, and D. For all values of s, the generalist ZNGIs go through the point $(R_0, R_0) = (0.2, 0.2)$.

Table S2.1: Parameters and state variables of the homogeneous and heterogeneous equation systems. Ranges or specific values are only shown for parameters that were used in numerical simulations of the nondimensionalized equation systems (Eqs. S2.5 and S2.8, respectively).

Quantity	Definition	Value/range	Units
u_i	Density of consumer i		mass area^{-1}
$R_{1,2}$	Densities of resources 1 and 2		mass area^{-1}
G	Per-capita growth rate function for the consumers		time^{-1}
$G_{\max}$	Maximal per-capita growth rate of consumers		time^{-1}
μ	Background mortality rate of consumers		time^{-1}
r	Resource renewal rate		time^{-1}
$K_{1,2}$	Local supply density of resources 1 and 2†		mass area^{-1}
K	Mean supply density of resources 1 and 2†		mass area^{-1}
C_1	Function describing the proportional consumption of resource 1		-
C_2	Function describing the proportional consumption of resource 2		-
d	Diffusion rate of the consumers		area time^{-1}
d_R	Diffusion rate of the resources		area time^{-1}
s	Control parameter for resource substitutability	$[-1, 1]$	-
a_{i1}	Affinity of consumer i for resource 1		area mass^{-1} time^{-1}
a_{i2}	Affinity of consumer i for resource 2		area mass^{-1} time^{-1}
$a_{\max}$	Maximal affinity when $a_{i1} = a_{i2}$	1	area mass^{-1} time^{-1}
a_0	Curvature parameter of the affinity trade-off	$[-5.95, 24.2]$	-
b	Shape parameter of the affinity trade-off	4	-
L	Length of side of square environment		length
$x_{1,2}$	Spatial coordinates		length
α	Scaled maximal per-capita growth	1/2	-
β	Scaled resource-dependent growth parameter	1/10	-
γ	Scaled resource renewal rate	1	-
δ	Scaled consumer diffusion rate	10^{-4}	-
δ_R	Scaled resource diffusion rate	10^{-4}	-
χ_i	Trait value of consumer i	$[-1, 1]$	-

†: For the homogeneous system, the supply density of both resources is K. For the heterogeneous system, the local supply of the two resources varies in space, but fulfills that $(1/L^2)\int K_1(\mathbf{x})\mathrm{d}\mathbf{x} = (1/L^2)\int K_2(\mathbf{x})\mathrm{d}\mathbf{x} = K$.

With the given specification above, s is a parameter that controls to what extent resources are substitutable, and a_0 is a parameter that controls to what extent the trade-off favors either generalists or specialists (Eq. S2.4). However, to make meaningful predictions about when coexistence will be possible, we do not study the effects of s and a_0 directly, but rather calculate the curvature of the generalist ZNGIs and the trade-off curves. Letting the curvature of the generalist ZNGI multiplied by R_0 be κ_{Z} evaluated at the point $R_1 = R_2 = R_0$, and the curvature of the trade-off curve be κ_{T}, evaluated at the point $a_1 = a_2 = 1$, we can calculate the expressions

$$\kappa_{\mathrm{Z}} = \frac{1}{\sqrt{2}}(1-s) \tag{S2.6a}$$

$$\kappa_{\mathrm{T}} = \frac{1}{\sqrt{2}} b \frac{(1+b)a_0 - b\log\left(\frac{1}{2}(\exp(a_0)+1)\right)}{(b+2)^2}\,. \tag{S2.6b}$$

S2.1.2 Heterogeneous environments

Our generic equations for resource competition in heterogeneous environments, which extend the spatially homogeneous model in the main text (Eqs. 1) read

$$\frac{\partial u_i(\mathbf{x},t)}{\partial t} = G(a_{i1}R_1, a_{i2}R_2)u_i - \mu u_i + d\Delta u_i \tag{S2.7a}$$

$$\frac{\partial R_1(\mathbf{x},t)}{\partial t} = r(K_1(\mathbf{x}) - R_1) - \sum_{i=1}^{N} C_1(a_{i1}R_1, a_{i2}R_2)G(a_{i1}R_1, a_{i2}R_2)u_i + d_R\Delta R_1 \tag{S2.7b}$$

$$\frac{\partial R_2(\mathbf{x},t)}{\partial t} = r(K_2(\mathbf{x}) - R_2) - \sum_{i=1}^{N} C_2(a_{i1}R_1, a_{i2}R_2)G(a_{i1}R_1, a_{i2}R_2)u_i + d_R\Delta R_2\,. \tag{S2.7c}$$

We specify the per capita growth functions, proportions of consumption, and trade-off functions in the same way as above for the spatially homogeneous case (Eqs. S2.2–S2.4). We let $1/L^2 \int K_1(\mathbf{x})\mathrm{d}\mathbf{x} = 1/L^2 \int K_2(\mathbf{x})\mathrm{d}\mathbf{x} = K$, and $\mathbf{x} = (x_1, x_2) \in (0, L]^2$. The term $d\Delta u_i$ describes the random movement or transport in space of consumers where $\Delta = \partial^2/\partial x_1^2 + \partial^2/\partial x_2^2$ is the two-dimensional Laplacian, and d is the diffusion rate of consumers (see Britton 1986; Cantrell & Cosner 2004; Vinatier *et al.* 2011 for more on reaction-diffusion models). Similarly, the term $d_R\Delta R_j$ describes the random movement or transport of resources in space, and d_R is the resource diffusion rate. The boundary conditions are periodic in both spatial directions, so that the dynamics take place on a periodic square with side length L.

We once again nondimensionalize this system and let $\widehat{t} = t(G_{\max} - \mu)$, $\widehat{u}_i = u_i/K$, $\widehat{R}_j = R_j/K$, $\widehat{K_1} = K_1/K$, $\widehat{K_2} = K_2/K$, and $\widehat{\mathbf{x}} = (x_1/L, x_2/L)$. This yields the set of scaled equations

$$\frac{\partial \widehat{u}_i(\widehat{\mathbf{x}},\widehat{t})}{\partial \widehat{t}} = \widehat{G}(\widehat{a}_{i1}\widehat{R}_1, \widehat{a}_{i2}\widehat{R}_2)\widehat{u}_i + \left(1 - \frac{1}{\alpha}\right)\widehat{u}_i + \delta\Delta\widehat{u}_i \tag{S2.8a}$$

$$\frac{\partial \widehat{R}_1(\widehat{\mathbf{x}},\widehat{t})}{\partial \widehat{t}} = \gamma(\widehat{K_1}(\widehat{\mathbf{x}}) - \widehat{R}_1) - \sum_{i=1}^{N} C_1(\widehat{a}_{i1}\widehat{R}_1, \widehat{a}_{i2}\widehat{R}_2)\widehat{G}(\widehat{a}_{i1}\widehat{R}_1, \widehat{a}_{i2}\widehat{R}_2)\widehat{u}_i + \delta_R\Delta\widehat{u}_i \tag{S2.8b}$$

$$\frac{\partial \widehat{R}_2(\widehat{\mathbf{x}},\widehat{t})}{\partial \widehat{t}} = \gamma(\widehat{K_2}(\widehat{\mathbf{x}}) - \widehat{R}_2) - \sum_{i=1}^{N} C_2(\widehat{a}_{i1}\widehat{R}_1, \widehat{a}_{i2}\widehat{R}_2)\widehat{G}(\widehat{a}_{i1}\widehat{R}_1, \widehat{a}_{i2}\widehat{R}_2)\widehat{u}_i + \delta_R\Delta\widehat{u}_i \tag{S2.8c}$$

$$\delta = \frac{d}{(G_{\max} - \mu)L^2}\,, \qquad \delta_R = \frac{d_R}{(G_{\max} - \mu)L^2}\,, \tag{S2.8d}$$

where the functions and parameters $\widehat{G}$, C_1, C_2, $\hat{a}_{i1}$, $\hat{a}_{i2}$, α, β, and γ are the same as in homogeneous environments (Eqs. S2.5). All parameters of the nondimensionalized model (Eqs. S2.8) are defined and listed with their values in Table S2.1.

S2.2 Numerical methods

To illustrate our analytical predictions for homogeneous environments and to determine outcomes for heterogeneous environments, we performed extensive numerical simulations Below, we describe in detail the numerical methods that we used to simulate the ecological and evolutionary dynamics in both homogeneous and heterogeneous environments.

S2.2.1 Homogeneous environments

To solve the nondimensionalized equations describing the ecological dynamics (Eqs. S2.5), we used the routine `ode15s` in Matlab (Mathworks 2016), and integrated this in time until the invasion fitness, i.e., the per capita net growth of each consumer was less than 10^{-10}. After this, we simultaneously solved Eqs. S2.5 together with the equations

$$\frac{\mathrm{d}\chi_i}{\mathrm{d}t} = \epsilon \mathcal{D}_i = \epsilon \left. \frac{\partial \widehat{G}(\widehat{a}_{i1}(\chi)\widehat{R}_1, \widehat{a}_{i2}(\chi)\widehat{R}_2)}{\partial \chi} \right|_{\chi=\chi_i} , \tag{S2.9}$$

which describes the evolution over time of the trait value χ_i of consumer i, which sets the affinities $\widehat{a}_{i1}(\chi_i)$ and $\widehat{a}_{i2}(\chi_i)$. The quantity $\mathcal{D}_i$ is the selection gradient for consumer i. Equation S2.9 relies on a separation of time scales, where ϵ is a small number (10^{-6}). This ensures that the traits change slowly on ecological time scales, so that the ecological dynamics are always close to being in ecological equilibrium.

Once all selection gradients are sufficiently close to zero ($|\mathcal{D}_i| < 10^{-10}$), we test whether any mutant with trait value $\chi_{\mathrm{inv}} \in [-1, 1]$ can invade the ensemble of residents. If so, we insert the invader with the trait value that yields maximal invasion fitness into the ensemble of consumers and repeat the steps above. If no further invasions are possible we conclude that we have reached the maximally diverse ensemble of consumers coexisting evolutionarily stably. As we consider competition for two resources and equilibrium dynamics, this entails either one or two consumers.

S2.2.2 Heterogeneous environments

We discretized and solved the nondimensionalized equations describing the ecological dynamics in spatially heterogeneous environments (Eqs. S2.8), which we characterized by random resource supplies $K_1(\mathbf{x})$ and $K_2(\mathbf{x})$. To generate random resource supplies we discretized the periodic unit square into a 64×64 grid with coordinates (i, j), with $i, j \in \{1/64, 2/64, 3/64, ..., 1\}$. We then used Perlin noise (Perlin 1985) to generate 2 random autocorrelated 64×64 matrices, L_1 and L_2, which are both positive matrices normalized to have a mean value of 1. To set a specific correlation ρ between the discretized resource supplies K_1 and K_2 we used the following procedure.

Let l_1 and l_2 be the $64^2 \times 1$ vectors obtained by stacking all the columns of the matrices L_1 and L_2 respectively on top of one another. Let $l_{1\mathrm{c}} = l_1 - 1$ and $l_{2\mathrm{c}} = l_2 - 1$, which are centered versions of l_1 and l_2 with mean zero. Next, let $\hat{l}_{1\mathrm{c}} = l_{1\mathrm{c}}/\|l_{1\mathrm{c}}\|$ and $\hat{l}_{2\mathrm{c}} = l_{2\mathrm{c}}/\|l_{2\mathrm{c}}\|$, where the norms are the standard Euclidean vector norms. Let $l_\perp = \hat{l}_{2\mathrm{c}} - (\hat{l}_{2\mathrm{c}} \cdot \hat{l}_{1\mathrm{c}})\hat{l}_{1\mathrm{c}}$, where $\cdot$ denotes the scalar vector product. This will ensure that the vector $l_\perp$ is orthogonal to $\hat{l}_{1\mathrm{c}}$. Finally, we let either

$$\begin{cases} \hat{k}_2 = \sigma_1(\rho \hat{l}_{1\mathrm{c}} + \sqrt{1-\rho^2} l_\perp) + 1 \,, & (i) \\ \hat{k}_2 = m(\rho \hat{l}_{1\mathrm{c}} + \sqrt{1-\rho^2} l_\perp) + 1 \,, & (ii) \end{cases} \tag{S2.10}$$

where σ_1 is the standard deviation of l_1. We pick case (i) if all values of $\hat{k}_2$ are greater than 0.05, otherwise we pick case (ii), where m is a constant chosen so that the minimal value of $\hat{k}_2$ is 0.05. We let $\hat{k}_1 = l_1$, and reshape these vectors to be two 64×64 matrices, which gives us our scaled resource supply matrices $\widehat{K}_1$ and $\widehat{K}_2$, which are our discretizations of $\widehat{K}_1(\mathbf{x})$ and $\widehat{K}_2(\mathbf{x})$ in Eqs. S2.8.

To solve the ecological dynamics described by Eqs. S2.8, we discretized space using so-called pseudospectral methods (Trefethen 2000), where any quantity on the 64×64 grid described above (e.g., $\widehat{K}_1$) represents a trigonometric polynomial. This trigonometric polynomial can be reevaluated at arbitrary

spatial coordinates, and the figures showing spatial densities, such as Fig. 2 in the main text, show the trigonometric polynomial evaluated at 1024×1024 points.

As for the resource supplies, we considered $64^2 \times 1$ vectors that enumerate all the grid points in the 64×64 grid, and thus get spatially discretized versions of Eqs. S2.8

$$\frac{\mathrm{d}\mathbf{u}_i}{\mathrm{d}t} = \mathbf{G}(a_{i1}\mathbf{R}_1, a_{i2}\mathbf{R_2}) \odot \mathbf{u}_i + (1 - \frac{1}{\alpha})\mathbf{u}_i + \delta D_\triangle \mathbf{u}_i \tag{S2.11a}$$

$$\frac{\mathrm{d}\mathbf{R}_1}{\mathrm{d}t} = \gamma(\mathbf{K}_1 - \mathbf{R}_1) - \sum_{i=1}^{N} \mathbf{C}_1(a_{i1}\mathbf{R}_1, a_{i2}\mathbf{R_2}) \odot \mathbf{G} \odot \mathbf{u}_i + \delta_R D_\triangle \mathbf{R}_1 \tag{S2.11b}$$

$$\frac{\mathrm{d}\mathbf{R}_2}{\mathrm{d}t} = \gamma(\mathbf{K}_2 - \mathbf{R}_2) - \sum_{i=1}^{N} \mathbf{C}_2(a_{i1}\mathbf{R}_1, a_{i2}\mathbf{R_2}) \odot \mathbf{G} \odot \mathbf{u}_i + \delta_R D_\triangle \mathbf{R}_2\,. \tag{S2.11c}$$

Here, $\odot$ denotes element-wise vector multiplication, and $D_\triangle$ is a $64^2 \times 64^2$ square matrix, that approximates the Laplacian operator, as given in Trefethen (2000) for periodic square domains. The various bold symbols in the equation system above are vectors, which are the spatially discrete versions of the functions in Eqs. S2.8.

We performed the time-stepping of the semi-discretized equations using exponential integrator methods (Hochbruck & Ostermann 2010). We followed the same evolutionary assembly procedure as for the spatially homogeneous environments (section S2.2.1), where we used the methods of Wickman *et al.* (2017) to calculate the invasion fitnesses of rare mutants, and the selection gradients of the residents. As for the homogeneous case, this produces communities of several consumers that are closed to invasions by rare mutants.

S2.3 Additional examples of models that fit the generic equations

The generic equations from which we derived analytical results encompass a broad range of resource uptake and growth functions. In section S2.1.1 we described the specific implementation that allowed us to vary the growth function continuously from fully substitutable to essential resources (Eqs. S2.2–S2.4). To illustrate the braod scope of the generic equations (Eqs. 1 in the main text, restated as Eqs. S2.1) we list in the following a few additional, widely used models that represent specific cases of these generic equations.

S2.3.1 Lotka-Volterra dynamics

The simplest possible system fulfilling the generic equations and their associated assumptions describes resource–consumer interactions by Lotka-Volterra dynamics, which yields the equations

$$\frac{\mathrm{d}u_i(t)}{\mathrm{d}t} = (a_{i1}R_1 + a_{i2}R_2)u_i - \mu u_i \tag{S2.12a}$$

$$\frac{\mathrm{d}R_1(t)}{\mathrm{d}t} = r(K - R_1) - \sum_{i=1}^{N} a_{i1}R_1u_i \tag{S2.12b}$$

$$\frac{\mathrm{d}R_2(t)}{\mathrm{d}t} = r(K - R_2) - \sum_{i=1}^{N} a_{i2}R_2u_i\,. \tag{S2.12c}$$

Here, a_{i1} and a_{i2} are the consumers' attack rates on the two resources. Comparing with Eqs. S2.1, we can through some algebra compute that

$$G(a_{i1}R_1, a_{i2}R_2) = a_{i1}R_1 + a_{i2}R_2 \tag{S2.13a}$$

$$C_1(a_{i1}R_1, a_{i2}R_2) = \frac{a_{i1}R_1}{a_{i1}R_1 + a_{i2}R_2} \tag{S2.13b}$$

$$C_2(a_{i1}R_1, a_{i2}R_2) = \frac{a_{i2}R_2}{a_{i1}R_1 + a_{i2}R_2}\,. \tag{S2.13c}$$

From this, we see that the symmetries $G(X,Y) = G(Y,X)$ and $C_1(X,Y) = C_2(Y,X)$ are fulfilled, so that the resources, from a consumer's point of view, are only distinguished by the consumer's ability to specialize on either one. It is also evident that $\partial C_1/\partial X > 0$, so that the proportion of consumption of resource 1 increases when the effective availability of resource 1, $a_{i1}R_1$, increases. Note that Eqs. S2.12 assume that the conversion efficiency of consumed resources into consumer biomass equals 1. Our results hold also in the more general case of a conversion efficiency < 1, provided that this conversion efficiency is the same for both resources.

S2.3.2 Holling type II functional response

A common model for the uptake of substitutable resources is the Holling type II functional response model, where the growth of consumers saturates at high resource densities due to a handling time, so that consumers cannot process resources instantaneously. The equations are described by

$$\frac{\mathrm{d}u_i(t)}{\mathrm{d}t} = \frac{a_{i1}R_1 + a_{i2}R_2}{1 + ha_{i1}R_1 + ha_{i2}R_2}u_i - \mu u_i \tag{S2.14a}$$

$$\frac{\mathrm{d}R_1(t)}{\mathrm{d}t} = r(K - R_1) - \sum_{i=1}^{N} \frac{a_{i1}R_1}{1 + ha_{i1}R_1 + ha_{i2}R_2}u_i \tag{S2.14b}$$

$$\frac{\mathrm{d}R_2(t)}{\mathrm{d}t} = r(K - R_2) - \sum_{i=1}^{N} \frac{a_{i2}R_2}{1 + ha_{i1}R_1 + ha_{i2}R_2}u_i\,. \tag{S2.14c}$$

Some algebra once again yields that

$$G(a_{i1}R_1, a_{i2}R_2) = \frac{a_{i1}R_1 + a_{i2}R_2}{1 + ha_{i1}R_1 + ha_{i2}R_2} \tag{S2.15a}$$

$$C_1(a_{i1}R_1, a_{i2}R_2) = \frac{a_{i1}R_1}{a_{i1}R_1 + a_{i2}R_2} \tag{S2.15b}$$

$$C_2(a_{i1}R_1, a_{i2}R_2) = \frac{a_{i2}R_2}{a_{i1}R_1 + a_{i2}R_2}\,. \tag{S2.15c}$$

We see that as for the Lotka-Volterra equations, the symmetry conditions hold, and that the proportions of consumption are the same as for the Lotka-Volterra case. We can also see that the symmetry condition for the growth rate function only holds because the handling times h are the same for both resources. If the handling times were different for the two resources, they would no longer be indistinguishable from the perspective of the consumers.

S2.3.3 A classic minimum model

A model often used to describe resource competition for essential resources is described by

$$\frac{\mathrm{d}u_i(t)}{\mathrm{d}t} = G_{\max} \min\left\{\frac{R_1}{H_{i1}+R_1}, \frac{R_2}{H_{i2}+R_2}\right\} u_i - \mu u_i \tag{S2.16a}$$

$$\frac{\mathrm{d}R_1(t)}{\mathrm{d}t} = r(K-R_1) - \sum_{i=1}^{N} q_{i1} G_{\max} \min\left\{\frac{R_1}{H_{i1}+R_1}, \frac{R_2}{H_{i2}+R_2}\right\} u_i \tag{S2.16b}$$

$$\frac{\mathrm{d}R_2(t)}{\mathrm{d}t} = r(K-R_2) - \sum_{i=1}^{N} q_{i2} G_{\max} \min\left\{\frac{R_1}{H_{i1}+R_1}, \frac{R_2}{H_{i2}+R_2}\right\} u_i\,. \tag{S2.16c}$$

Here, consumers are assumed to depend on their more limiting resource as described by a Monod function, with half-saturation constants H_{i1} and H_{i2} for resources 1 and 2 respectively. Consumers are assumed to require q_{i1} and q_{i2} units of resources 1 and 2 respectively to construct one unit of consumer biomass. If we let $a_{i1} = 1/H_{i1}$, $a_{i2} = 1/H_{i2}$, and let $q_{i1} = q_{i2} = 1/2$ we get the system

$$\frac{\mathrm{d}u_i(t)}{\mathrm{d}t} = G_{\max} \min\left\{\frac{a_{i1}R_1}{1+a_{i1}R_1}, \frac{a_{i2}R_2}{1+a_{i2}R_2}\right\} u_i - \mu u_i \tag{S2.17a}$$

$$\frac{\mathrm{d}R_1(t)}{\mathrm{d}t} = r(K-R_1) - \sum_{i=1}^{N} \frac{1}{2} G_{\max} \min\left\{\frac{a_{i1}R_1}{1+a_{i1}R_1}, \frac{a_{i2}R_2}{1+a_{i2}R_2}\right\} u_i \tag{S2.17b}$$

$$\frac{\mathrm{d}R_2(t)}{\mathrm{d}t} = r(K-R_2) - \sum_{i=1}^{N} \frac{1}{2} G_{\max} \min\left\{\frac{a_{i1}R_1}{1+a_{i1}R_1}, \frac{a_{i2}R_2}{1+a_{i2}R_2}\right\} u_i\,. \tag{S2.17c}$$

From this we can immediately see that

$$G(a_{i1}R_1, a_{i2}R_2) = G_{\max} \min\left\{\frac{a_{i1}R_1}{1+a_{i1}R_1}, \frac{a_{i2}R_2}{1+a_{i2}R_2}\right\} \tag{S2.18a}$$

$$C_1(a_{i1}R_1, a_{i2}R_2) = \frac{1}{2} \tag{S2.18b}$$

$$C_2(a_{i1}R_1, a_{i2}R_2) = \frac{1}{2}\,. \tag{S2.18c}$$

It is easy to see that $G(X,Y) = G(Y,X)$, so the per capita growth function fulfills the requirement of symmetry. In order for the proportions of consumption to be symmetrical, $C_1 = C_2 = 1/2$ is the only possible choice, as we require both that $C_1 + C_2 = 1$, and that $C_1(X,Y) = C_2(Y,X)$, in order for the resources to be the same from the consumers' point of view apart from the consumer's ability to specialize on either one through evolving affinities.

S2.3.4 A model for interactive-essential resources

When resources are interactively essential, growth can be modeled as a product of Monod functions. The following equations describe competition for interactive-essential resources in a way that fulfills the requirements of the general model (Eqs. S2.1):

$$\frac{\mathrm{d}u_i(t)}{\mathrm{d}t} = G_{\max} \frac{a_{i1}R_1}{1+a_{i1}R_1} \frac{a_{i2}R_2}{1+a_{i2}R_2} u_i - \mu u_i \tag{S2.19a}$$

$$\frac{\mathrm{d}R_1(t)}{\mathrm{d}t} = r(K - R_1) - \sum_{i=1}^{N} \frac{1 + a_{i1}R_1}{2 + a_{i1}R_1 + a_{i2}R_2} G_{\max} \frac{a_{i1}R_1}{1 + a_{i1}R_1} \frac{a_{i2}R_2}{1 + a_{i2}R_2} u_i \tag{S2.19b}$$

$$\frac{\mathrm{d}R_2(t)}{\mathrm{d}t} = r(K - R_2) - \sum_{i=1}^{N} \frac{1 + a_{i2}R_2}{2 + a_{i1}R_1 + a_{i2}R_2} G_{\max} \frac{a_{i1}R_1}{1 + a_{i1}R_1} \frac{a_{i2}R_2}{1 + a_{i2}R_2} \,. \tag{S2.19c}$$

Here, we have not derived C_1 and C_2, but rather assumed a form that lies somewhere in between those of the Lotka-Volterra and completely essential models.

References

Britton, N.F. (1986). *Reaction-diffusion equations and their applications to biology.* Academic Press, London.

Cantrell, R.S. & Cosner, C. (2004). *Spatial ecology via reaction-diffusion equations.* John Wiley & Sons, Chichester.

Hochbruck, M. & Ostermann, A. (2010). Exponential integrators. *Acta Numerica*, 19, 209–286.

Mathworks (2016). *MATLAB version 9.1.0.441655 (R2016b).* The Mathworks, Inc., Natick, Massachusetts.

Perlin, K. (1985). An image synthesizer. *ACM SIGGRAPH Comput. Graphics*, 19, 287–296.

Schreiber, S.J. & Tobiason, G.A. (2003). The evolution of resource use. *J. Math. Biol.*, 47, 56–78.

Trefethen, L.N. (2000). *Spectral methods in MATLAB.* vol. 10 of *Software Environments Tools.* Siam, Philadelphia, PA.

Vinatier, F., Tixier, P., Duyck, P.F. & Lescourret, F. (2011). Factors and mechanisms explaining spatial heterogeneity: a review of methods for insect populations. *Methods Ecol. Evol.*, 2, 11–22.

Wickman, J., Diehl, S., Blasius, B., Klausmeier, C.A., Ryabov, A. & Brännström, Å. (2017). Determining selection across heterogeneous landscapes: a perturbation-based method and its application to modeling evolution in space. *Am. Nat.*, 189, 381–395.
