# Supplementary material for: Evolution of resource specialisation in competitive metacommunities
Source: Ecol Lett. 2019 Aug 7;22(11):1746–56. doi: 10.1111/ele.13338 (PMC6852178; doi:10.1111/ele.13338)
Supplement: Supplementary file 3 [file ELE-22-1746-s003.pdf]

Appendix S3: Robustness results

In this appendix we show that our results are, at least qualitatively, robust to various changes in our models. We first show that our results in homogeneous environments also extend to antagonistic resources. We then explore to what extent the homogeneous model is robust towards relaxing our various assumptions about resource symmetry. Finally, we show that the results in heterogeneous environments are qualitatively robust to changes in the resource-supply landscapes.

S3.1 Antagonistic resources

In this paper, we have focused on exploring the spectrum of resource types from substitutable to essential, as these encompass a majority of resource-competition scenarios. However, our analytical results are also valid for antagonistic resources, where the zero net growth isoclines (ZNGIs) of consumers are concave in resource space, implying that consumers require less total resources the more they specialize on either resource. In our general formulation, resources are antagonistic if $\kappa_\text{Z} < 0$. Since we never made any assumptions in our analytical derivations that resources could not be antagonistic, the conditions that evolutionarily stable coexistence is possible if and only if $\kappa_Z < 1/\sqrt{2}$ and $\kappa_\text{Z} < \kappa_\text{T}$ still continue to hold for antagonistic resources. In this section we illustrate how our specific formulation of the homogeneous model (Eqs. S2.1–S2.4) can be extended to include antagonistic resources.

In our specific implementation, resources are antagonistic when the control parameter for the degree of resource substitutability $s > 1$, and the greater s becomes the more antagonistic the resources are. As in other parts of this manuscript, we let κ_Z be the curvature of the invasion boundary, and κ_T be the curvature of the trade-off curve, both evaluated at the point $a_1 = a_2 = 1$ in affinity space. To extend our numerical simulations to antagonistic resources ($\kappa_\text{Z} < 0$), we distinguish two cases. (i) When $\kappa_\text{T} \geq 0$, i.e. when trade-offs are linear or specialist-favoring, we let the trade-off be as in Eqs. S2.4. (ii) When $\kappa_\text{T} < 0$, i.e., when trade-offs are generalist-favoring, Eqs. S2.4 can, however, violate our assumption that the trade-off curve should lie entirely below the invasion boundary of consumers (i.e., the ZNGI scaled into affinity space as in Fig. S1.1) when $\kappa_\text{T} < \kappa_\text{Z}$. In this case, we let the trade-off be given by

$$a_1 = (1-\chi)^{a_0} \tag{S3.1a}$$

$$a_2 = (1+\chi)^{a_0}\,, \tag{S3.1b}$$

for $\chi \in [-1, 1]$. This ensures that if $\kappa_\text{T} < \kappa_\text{Z}$, then the trade-off curve lies entirely below the invasion boundary. We can compute the trade-off curvature at the point $a_1 = a_2 = 1$ to be

$$\kappa_\text{T} = \frac{1}{\sqrt{2}}\frac{a_0 - 1}{a_0}\,. \tag{S3.2}$$

We performed numerical simulations for antagonistic resources for both of the above cases, using otherwise the same equations and parameter values as for substitutable and essential resources (see Appendix S2.1.1). These simulations extend the results depicted in Fig. 4 of the main text seamlessly in the direction of antagonsistic resources. Specifically, when resources are antagonistic, evolutionarily stable coexistence of two consumers becomes possible also for generalist-favoring trade-offs ($\kappa_\text{T} < 0$) when the condition $\kappa_\text{Z} < \kappa_\text{T}$ is fulfilled (see Fig. S3.1, where we have normalized the curvatures as $\hat{\kappa}_\text{T} = \sqrt{2}\kappa_\text{T}$ and $\hat{\kappa}_\text{Z} = \sqrt{2}\kappa_\text{Z}$).

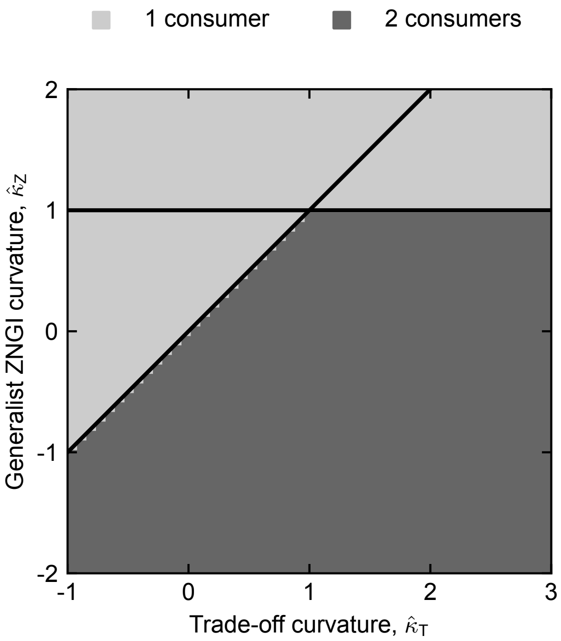

Figure S3.1: Extension of Fig. 4 in the main text, including antagonistic resources. Resources are antagonistic for $\hat{\kappa}_Z < 0$. The diagonal line indicates the condition $\hat{\kappa}_Z = \hat{\kappa}_T$. Evolutionarily stable coexistence of two consumers is possible in the dark gray area and impossible in the light gray area.

S3.2 Robustness with respect to asymmetries in the homogeneous model

To derive analytical results, one of our assumptions has been that resources have identical properties from the perspective of a consumer, with the exception that the consumer can specialize on one or the other resource by varying its affinities. This imposes a number of symmetry conditions on the equations describing the resource-competition system. Here, we investigate numerically to what extent our conclusions hold when these symmetries are broken.

The specific system we use for numerical investigations is described in detail in Appendix S2.1.1, and the nondimensionalized ecological dynamics are given by

$$\frac{\mathrm{d}\widehat{u}_i(\widehat{t})}{\mathrm{d}\widehat{t}} = \widehat{G}(\widehat{a}_{i1}\widehat{R}_1, \widehat{a}_{i2}\widehat{R}_2)\widehat{u}_i + \left(1 - \frac{1}{\alpha}\right)\widehat{u}_i \tag{S3.3a}$$

$$\frac{\mathrm{d}\widehat{R}_1(\widehat{t})}{\mathrm{d}\widehat{t}} = \gamma(\widehat{K} - \widehat{R}_1) - \sum_{i=1}^{N} C_1(\widehat{a}_{i1}\widehat{R}_1, \widehat{a}_{i2}\widehat{R}_2)\widehat{G}(\widehat{a}_{i1}\widehat{R}_1, \widehat{a}_{i2}\widehat{R}_2)\widehat{u}_i \tag{S3.3b}$$

$$\frac{\mathrm{d}\widehat{R}_2(\widehat{t})}{\mathrm{d}\widehat{t}} = \gamma(\widehat{K} - \widehat{R}_2) - \sum_{i=1}^{N} C_2(\widehat{a}_{i1}\widehat{R}_1, \widehat{a}_{i2}\widehat{R}_2)\widehat{G}(\widehat{a}_{i1}\widehat{R}_1, \widehat{a}_{i2}\widehat{R}_2)\widehat{u}_i \tag{S3.3c}$$

$$\widehat{G}(\widehat{a}_{i1}\widehat{R}_1, \widehat{a}_{i2}\widehat{R}_2) = \frac{1}{\alpha + \frac{\beta}{T(\widehat{a}_{i1}\widehat{R}_1, \widehat{a}_{i2}\widehat{R}_2)}}, \quad T(\widehat{a}_{i1}\widehat{R}_1, \widehat{a}_{i2}\widehat{R}_2) = 2^{-\frac{1}{s}}\left[(\widehat{a}_{i1}\widehat{R}_1)^s + (\widehat{a}_{i2}\widehat{R}_2)^s\right]^{\frac{1}{s}} \tag{S3.3d}$$

$$C_1(\widehat{a}_{i1}\widehat{R}_1, \widehat{a}_{i2}\widehat{R}_2) = \frac{(\widehat{a}_{i1}\widehat{R}_1)^E}{(\widehat{a}_{i1}\widehat{R}_1)^E + (\widehat{a}_{i2}\widehat{R}_2)^E}, \quad C_2(\widehat{a}_{i1}\widehat{R}_1, \widehat{a}_{i2}\widehat{R}_2) = \frac{(\widehat{a}_{i2}\widehat{R}_2)^E}{(\widehat{a}_{i1}\widehat{R}_1)^E + (\widehat{a}_{i2}\widehat{R}_2)^E}, \tag{S3.3e}$$

$$E = \exp(s - 1) \tag{S3.3f}$$

$$\hat{a}_{i1} = \frac{a_{i1}}{a_{\max}} = 1 + \frac{1}{a_0 - b \log\left(\frac{1}{2}(\exp(-a_0) + 1)\right)} \left(b \log\left(\frac{1}{2}(\exp(-a_0\chi_i) + 1)\right) - a_0\chi_i\right) \quad \text{(S3.3g)}$$

$$\hat{a}_{i2} = \frac{a_{i2}}{a_{\max}} = 1 + \frac{1}{a_0 - b \log\left(\frac{1}{2}(\exp(-a_0) + 1)\right)} \left(b \log\left(\frac{1}{2}(\exp(a_0\chi_i) + 1)\right) + a_0\chi_i\right) . \quad \text{(S3.3h)}$$

All symbols are defined in Table S2.1. We test, one by one, how breaking various symmetries of these equations affects how evolutionarily stable coexistence depends on the degree of resource substitutability and on the shape of the trade-off curve.

Below, we display analogues of Fig. 4 in the main text with various degrees of asymmetries for different parameters. In all these figures, the curvatures of both the generalist zero net growth isocline (ZNGI) and the trade-off curves, κ_{Z} and κ_{T}, were first calculated in the symmetric system, before the asymmetries were introduced. Apart from the imposed asymmetries, all parameter values are as in Table S2.1. It turns out that the different parameters we test are quantitatively differently sensitive to asymmetries. Yet, the qualitative pattern that emerges is similar, with the parameter space engendering coexistence shrinking as the asymmetries become stronger (Figs. S3.2–S3.9).

S3.2.1 Asymmetries in resource supplies

In the symmetric model given by Eqs. S3.3, the resource supplies are both equal so that the supply point is located on the diagonal in resource space, with $K_1 = K_2 = 1$. We test the effects of skewing the resource-supply ratios by assuming that

$$K_1 + K_2 = 2\,, \quad \frac{K_2}{K_1} = q\,, \tag{S3.4}$$

for $q = 0.75, 0.625, 0.5, 0.25$, with $\widehat{K_1} = 2/(1+q)$ and $\widehat{K_2} = 2q/(1+q)$. The results are depicted in Fig. S3.2. Reversed asymmetries ($q = 1/0.75$, $1/0.625$, $1/0.5$, $1/0.25$) would yield identical results.

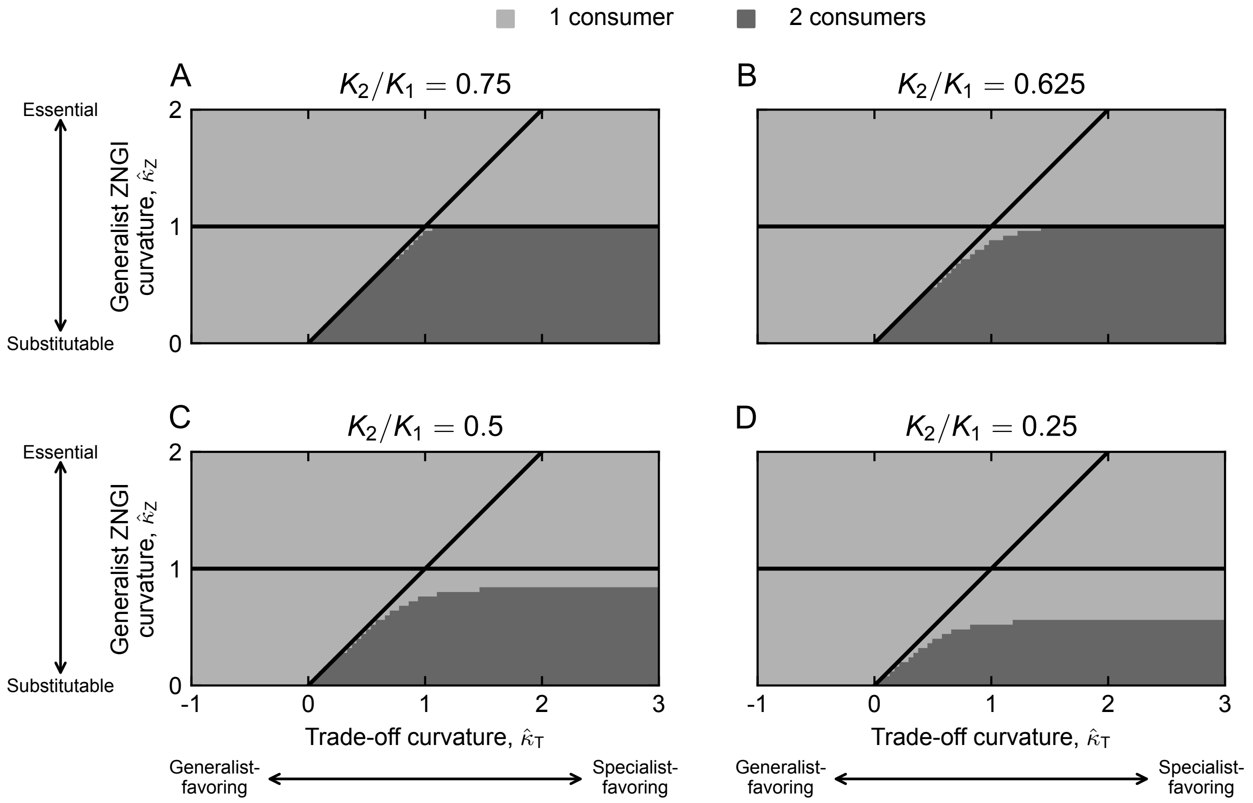

Figure S3.2: Effects of different degrees of asymmetry in resource supplies (indicated by the ratio $K_2/K_1 < 1$) on evolutionarily stable coexistence as a function of resource-type (substitutable to essential) and the shape of the afffinity trade-off (generalist- to specialist-favoring). The generalist zero net growth isocline (ZNGI) curvatures and trade-off curvatures were calculated in the symmetric system before the asymmetry in resource supply was introduced, and the panels can thus be compared with Fig. 4 in the main text. Each dot is the outcome of a numerical simulation, where light gray dots indicate that a single consumer can persist with evolutionary stability, and dark gray dots indicate that two different consumers can coexist with evolutionary stability.

S3.2.2 Asymmetries in resource renewal rates

In the symmetric model given by Eqs. S3.3, the resource renewal rates r_j are both equal so that $\gamma_1 = \gamma_2 = \gamma = 1$, where $\gamma_j = r_j/(G_{\max} - \mu)$. We test the effects of skewing the resource renewal rates by assuming that

$$\gamma_1 + \gamma_2 = 2\,, \quad \frac{\gamma_2}{\gamma_1} = \frac{r_2}{r_1} = q\,, \tag{S3.5}$$

for $q = 0.75, 0.5, 0.33, 0.25$. The nondimensionalized resource renewal rates in Eqs. S3.3b and S3.3c then become $\gamma_1 = 2/(1+q)$ and $\gamma_2 = 2q/(1+q)$. The results are depicted in Fig. S3.3. Reversed asymmetries ($q = 1/0.75$, $1/0.5$, $1/0.33$, $1/0.25$) would yield identical results.

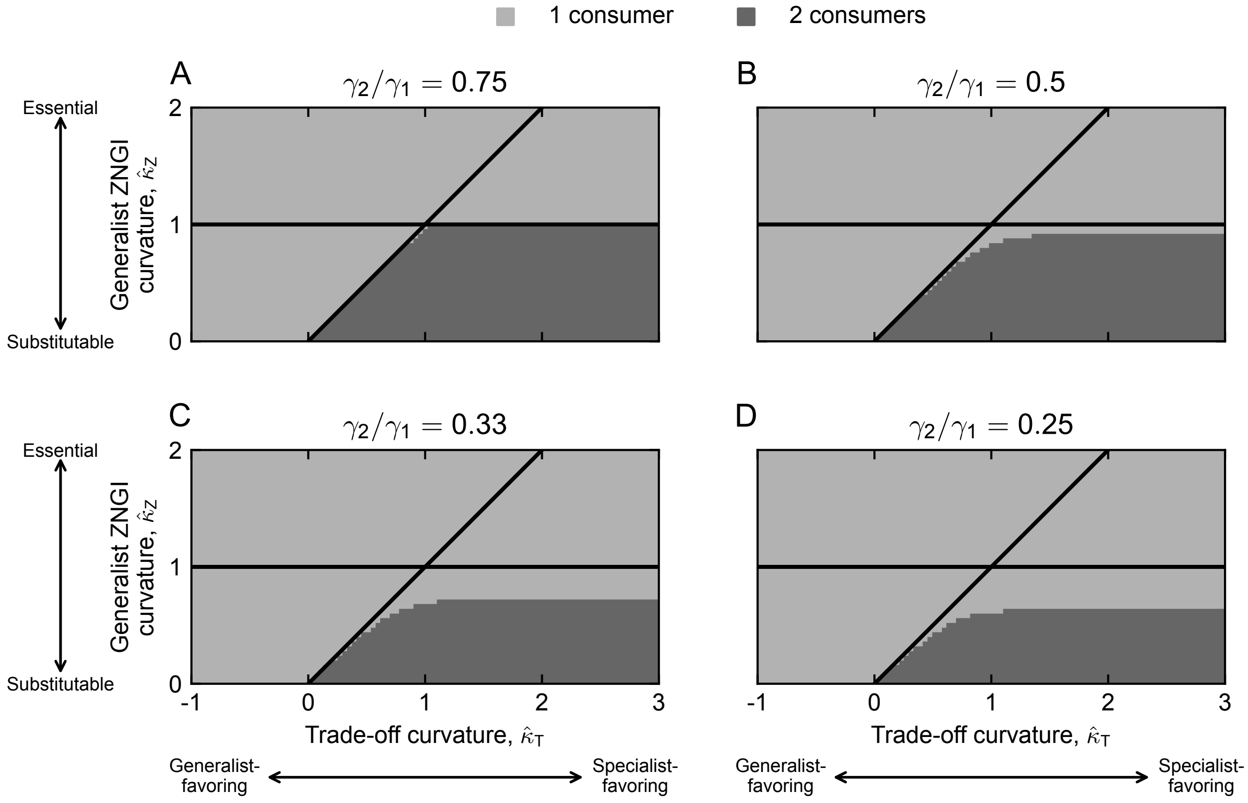

Figure S3.3: Effects of different degrees of asymmetry in resource renewal rates (indicated by the ratio $\gamma_2/\gamma_1 = r_2/r_1 < 1$) on evolutionarily stable coexistence as a function of resource-type (substitutable to essential) and the shape of the afffinity trade-off (generalist- to specialist-favoring). The generalist zero net growth isocline (ZNGI) curvatures and trade-off curvatures were calculated in the symmetric system before the asymmetry in resource renewal was introduced, and the panels can thus be compared with Fig. 4 in the main text. Each dot is the outcome of a numerical simulation, where light gray dots indicate that a single consumer can persist with evolutionary stability, and dark gray dots indicate that two different consumers can coexist with evolutionary stability.

S3.2.3 Asymmetries in resource affinities

In the symmetric model given by Eqs. S3.3, the resource affinities are described by a symmetric trade-off curve that goes through the point $a_1 = a_2 = 1$. We test the effects of skewing this trade-off curve by rescaling the nondimensionalized affinities in Eqs. S3.3 by letting the affinites of the consumers be given by new affinities $\tilde{a}_{i1}$ and $\tilde{a}_{i2}$ given by

$$\tilde{a}_{i1} = A_1 \hat{a}_{i1} \,, \quad \tilde{a}_{i2} = A_2 \hat{a}_{i2} \,, \tag{S3.6}$$

where the quantities A_1 and A_2 fulfill

$$A_1 + A_2 = 2 \,, \quad \frac{A_2}{A_1} = q \,, \tag{S3.7}$$

for $q = 0.5, 0.25, 0.15, 0.1$. This means that instead of going through the point $a_1 = a_2 = 1$ as in the symmetric case, all trade-offs now go through the point $(a_1, a_2) = (A_1, A_2) = (2/(1+q), 2q/(1+q))$, and are skewed so that the maximal affinity attainable for resource 2 is q times lower than for resource 1, see Fig. S3.4 for examples of skewed trade-offs. The results are depicted in Fig. S3.5. Reversed asymmetries ($q = 1/0.5$, $1/0.25$, $1/0.15$, $1/0.1$) would yield identical results.

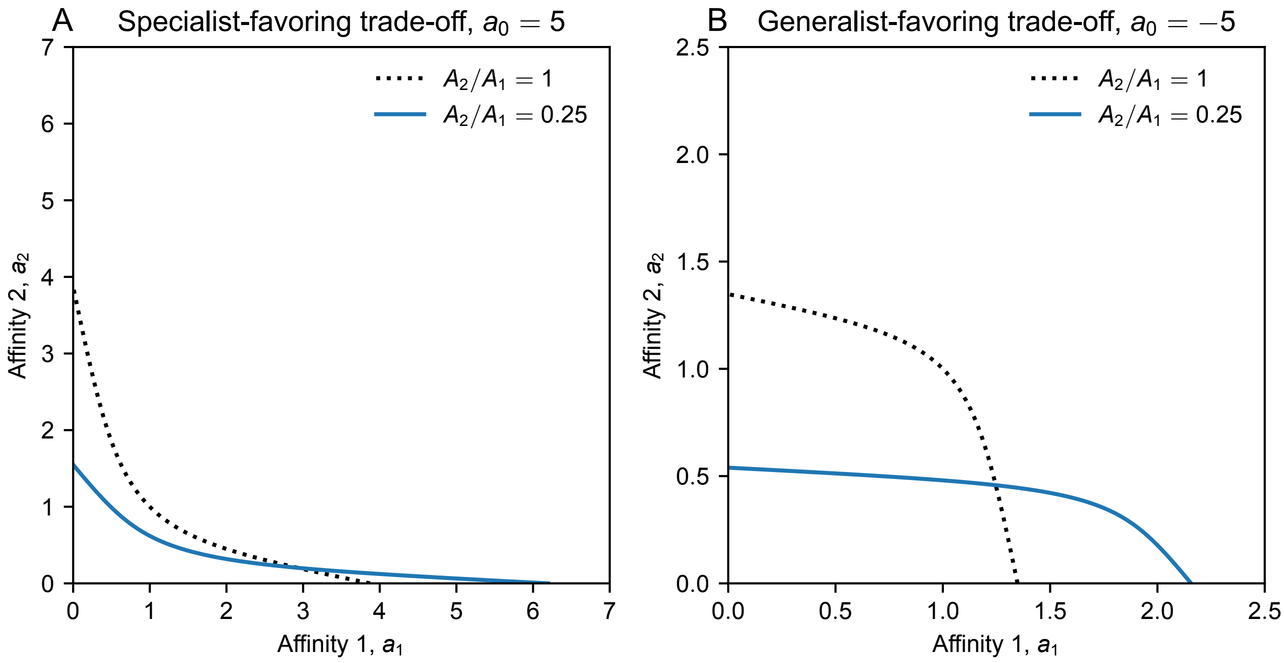

Figure S3.4: Examples of skewed trade-offs for a specialist-favoring trade-off (A), and a generalist-favoring trade-off (B). The dotted lines show the reference case, where the trade-off is symmetric, and the blue lines show skewed trade-offs with the skew factor being $A_2/A_1 = 0.25$. The trade-offs are drawn based on the trade-off specified in Eq. S2.4, with $b = 4$. In panel A, $a_0 = 5$, which implies that $\hat{\kappa}_T \approx 0.86$. In panel B, $a_0 = -5$, which implies that $\hat{\kappa}_T \approx -2.5$.

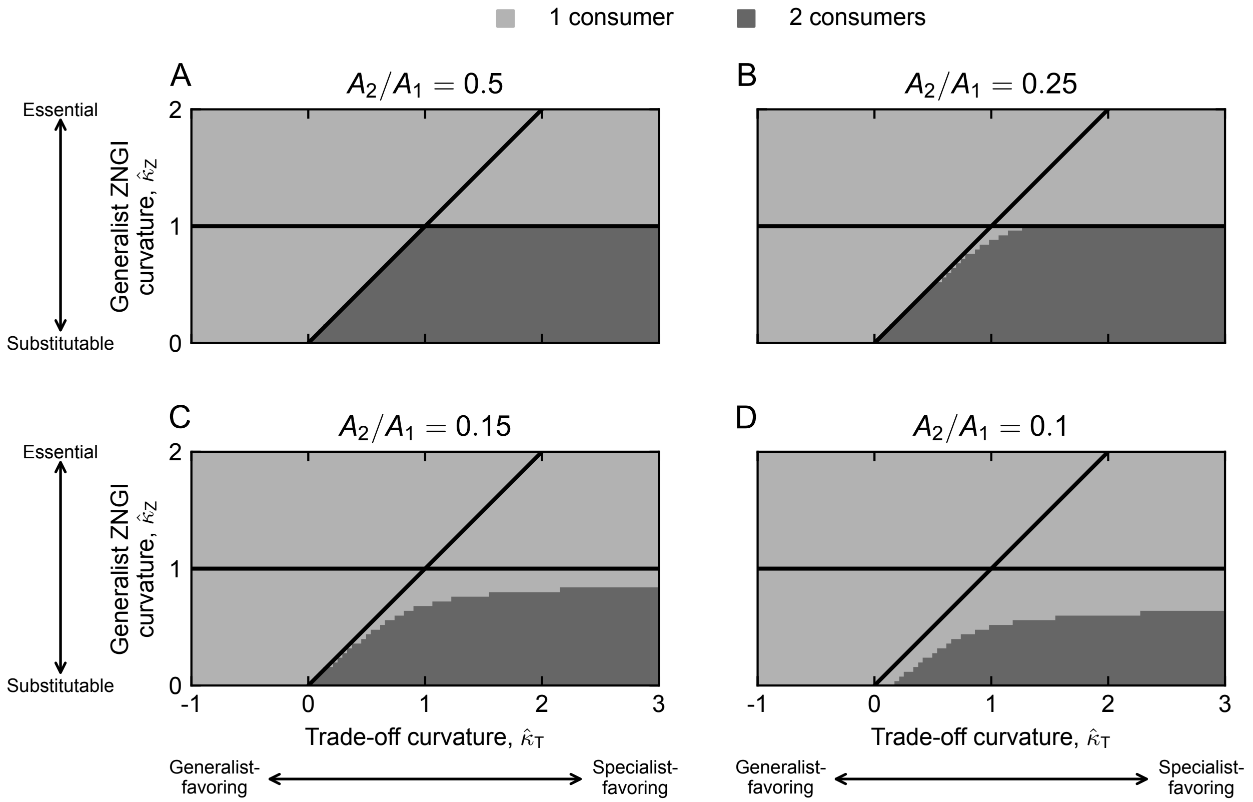

Figure S3.5: Effects of different degrees of asymmetry in affinity trade-off (expressed by the ratio of skew factors $A_2/A_1 < 1$) on evolutionarily stable coexistence as a function of resource-type (substitutable to essential) and the shape of the afffinity trade-off (generalist- to specialist-favoring). The generalist zero net growth isocline (ZNGI) curvatures and trade-off curvatures were calculated in the symmetric system before the asymmetry in the trade-off was introduced, and the panels can thus be compared with Fig. 4 in the main text. Each dot is the outcome of a numerical simulation, where light gray dots indicate that a single consumer can persist with evolutionary stability, and dark gray dots indicate that two different consumers can coexist with evolutionary stability.

S3.2.4 Asymmetries in the per capita growth function

In the symmetric model given by Eqs. S3.3, the per capita growth function is given by $\widehat{G}(\widehat{a}_{i1}\widehat{R}_1, \widehat{a}_{i2}\widehat{R}_2)$, and fulfilled that $\widehat{G}(X,Y) = \widehat{G}(Y,X)$, so that the growth function is symmetric in its arguments. We test deviations from this symmetry by introducing two parameters g_1 and g_2 that satisfy

$$g_1 + g_2 = 2\,, \quad \frac{g_2}{g_1} = q\,, \tag{S3.8}$$

for $q = 0.5, 0.33, 0.25, 0.15$. We then let the per capita growth function be given by

$$\widehat{G}(g_1\widehat{a}_1\widehat{R}_1, g_2\widehat{a}_2\widehat{R}_2) = \frac{1}{\alpha + \frac{\beta}{2^{-1/s}\left[(g_1\widehat{a}_1\widehat{R}_1)^s + (g_2\widehat{a}_2\widehat{R}_2)^s\right]^{1/s}}}\,. \tag{S3.9}$$

This skews the zero net growth isoclines of all consumers so that the resource requirements for resource 1 are lower than for resource 2, see Fig. S3.6 for examples of skewed ZNGIs. The results are depicted in Fig. S3.7. Reversed asymmetries ($q = 1/0.5$, $1/0.33$, $1/0.25$, $1/0.15$) would yield identical results.

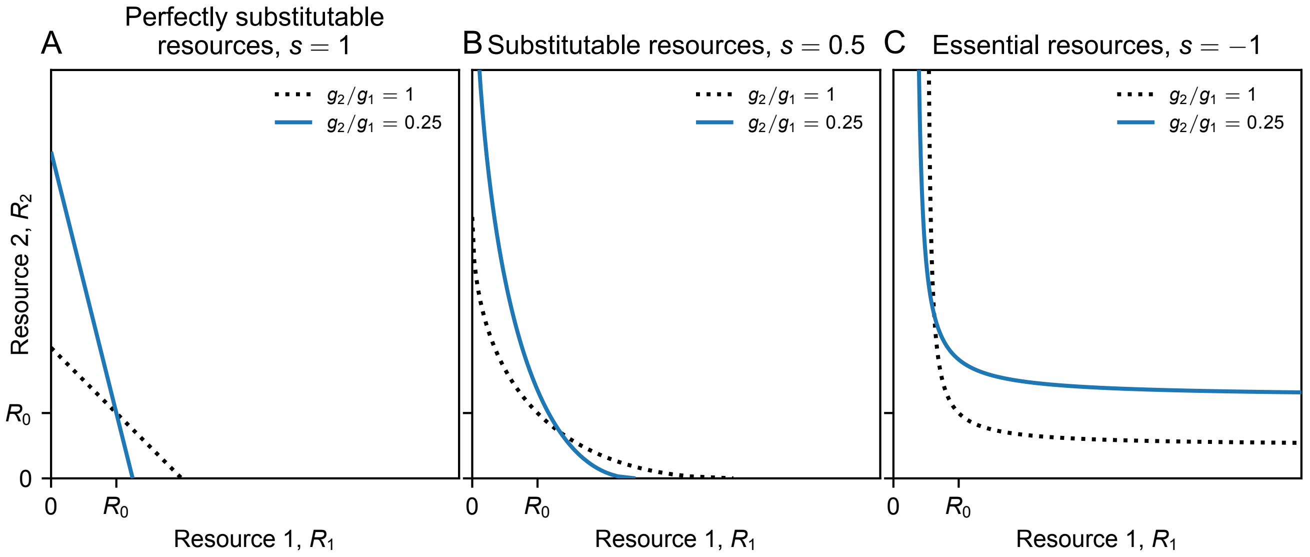

Figure S3.6: Examples of skewed generalist zero net growth isoclines (ZNGIs). The panels show generalist ZNGIs, i.e., ZNGIs of consumers with $a_1 = a_2 = 1$. The dotted lines show the symmetric reference case, and the blue lines show skewed generalist ZNGIs with the skew factor $g_2/g_1 = 0.25$. All ZNGIs were drawn based on Eqs. S2.5, and with parameter values as in Table S2.1. **A**: Perfectly substitutable resources with $s = 1$, which implies that $\hat{\kappa}_Z = 0$. **B**: Substitutable resources with $s = 0.5$, which implies that $\hat{\kappa}_Z = 0.5$. **C**: Essential resources with $s = -1$, which implies that $\hat{\kappa}_Z = 2$.

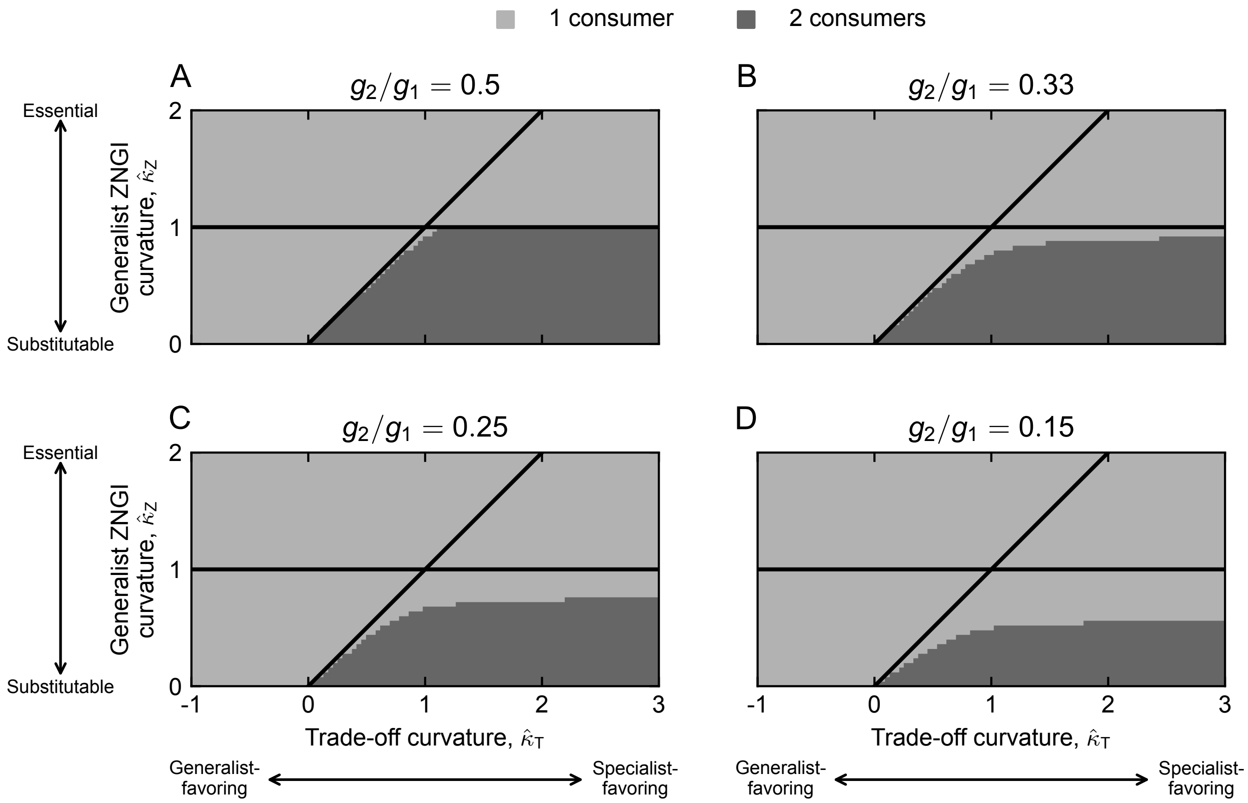

Figure S3.7: Effects of different degrees of asymmetry in growth functions (expressed by the ratio of skew factors $g_2/g_1 < 1$) on evolutionarily stable coexistence as a function of resource-type (substitutable to essential) and the shape of the affinity trade-off (generalist- to specialist-favoring). The generalist zero net growth isocline (ZNGI) curvatures and trade-off curvatures were calculated in the symmetric system before the asymmetry in the growth function was introduced, and the panels can thus be compared with Fig. 4 in the main text. Each dot is the outcome of a numerical simulation, where light gray dots indicate that a single consumer can persist with evolutionary stability, and dark gray dots indicate that two different consumers can coexist with evolutionary stability.

S3.2.5 Asymmetries in the proportions of consumption

In the symmetric model given by Eqs. S3.3, the proportions of consumption are given by $C_1(\widehat{a}_{i1}\widehat{R}_1, \widehat{a}_{i2}\widehat{R}_2)$ and $C_2(\widehat{a}_{i1}\widehat{R}_1, \widehat{a}_{i2}\widehat{R}_2)$, and fulfill the symmetry condition that $C_1(\widehat{a}_{i1}\widehat{R}_1, \widehat{a}_{i2}\widehat{R}_2) = C_2(\widehat{a}_{i2}\widehat{R}_2, \widehat{a}_{i1}\widehat{R}_1)$. We test deviations from this symmetry by introducing two parameters c_1 and c_2 that satisfy

$$c_1 + c_2 = 2\,, \quad \frac{c_2}{c_1} = q\,, \tag{S3.10}$$

for $q = 0.5, 0.25, 0.15, 0.1$. We then let the proportion of consumption functions be given by

$$C_1(c_1\widehat{a}_{i1}\widehat{R}_1, c_2\widehat{a}_{i2}\widehat{R}_2) = \frac{(c_1\widehat{a}_{i1}\widehat{R}_1)^E}{(c_1\widehat{a}_{i1}\widehat{R}_1)^E + (c_1\widehat{a}_{i2}\widehat{R}_2)^E} \tag{S3.11a}$$

$$C_2(c_1\widehat{a}_{i1}\widehat{R}_1, c_2\widehat{a}_{i2}\widehat{R}_2) = \frac{(c_2\widehat{a}_{i2}\widehat{R}_2)^E}{(c_1\widehat{a}_{i1}\widehat{R}_1)^E + (c_1\widehat{a}_{i2}\widehat{R}_2)^E}\,, \tag{S3.11b}$$

where $E = \exp(s-1)$. This skews the proportions of consumption for all consumers so that relatively more of resource 1 tends to be consumed, see Fig. S3.8 for examples of skewed consumption vectors. The results are depicted in Fig. S3.9. Reversed asymmetries (q = 1/0.5, 1/0.25, 1/0.15, 1/0.1) would yield identical results.

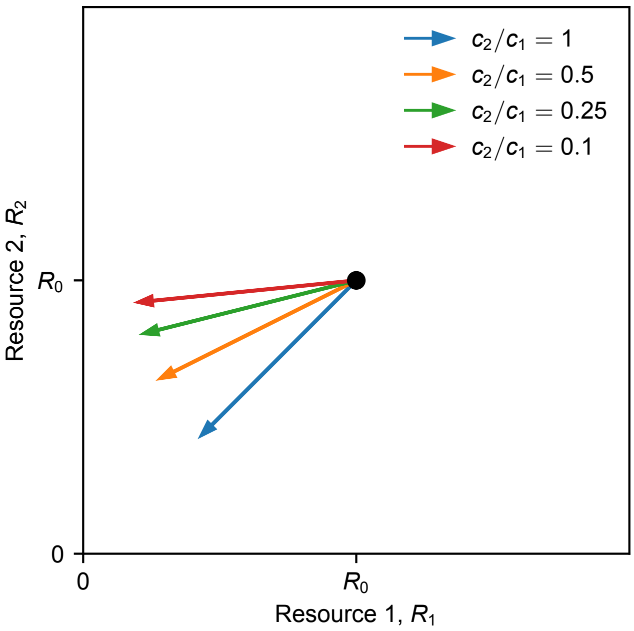

Figure S3.8: Examples of skewed consumption vectors. Each arrow depicts the vector $-(C_1, C_2)$, drawn with its base at the point (R_0, R_0), with $C_1 = C_1(c_1\widehat{a}_1\widehat{R}_1, c_2\widehat{a}_2\widehat{R}_2)$ and $C_2 = C_2(c_1\widehat{a}_1\widehat{R}_1, c_2\widehat{a}_2\widehat{R}_2)$, evaluated for $\widehat{R}_1 = \widehat{R}_2 = R_0$, and $\widehat{a}_1 = \widehat{a}_2 = 1$. C_1 and C_2 were calculated from Eqs. S3.11 with $s = 1$, using four different skew factors $c_2/c_1 = 1, 0.5, 0.25, 0.1$, where $c_2/c_1 = 1$ is the symmetric reference case. The lengths of the arrows were normalized to fit into the panel.

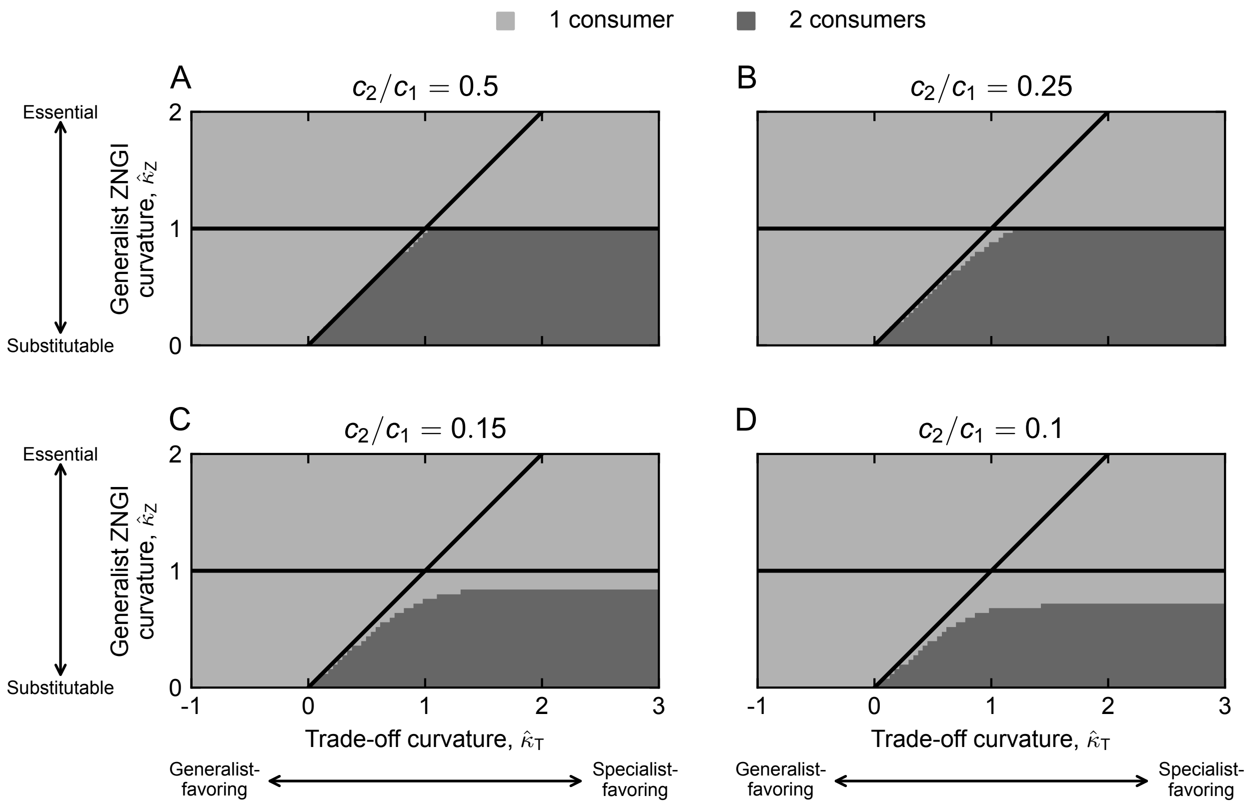

Figure S3.9: Effects of different degrees of asymmetry in proportions of consumption (expressed by the ratio of skew factors $c_2/c_1 < 1$) on evolutionarily stable coexistence as a function of resource-type (substitutable to essential) and the shape of the afffinity trade-off (generalist- to specialist-favoring). The generalist zero net growth isocline (ZNGI) curvatures and trade-off curvatures were calculated in the symmetric system before the asymmetry in proportional consumption was introduced, and the panels can thus be compared with Fig. 4 in the main text. Each dot is the outcome of a numerical simulation, where light gray dots indicate that a single consumer can persist with evolutionary stability, and dark gray dots indicate that two different consumers can coexist with evolutionary stability.

S3.3 Additional supply landscapes

To verify the robustness of our numerical simulations with respect to different configurations of supply landscapes, we performed numerical simulations for 3 different, randomly generated pairs of supply landscapes for resource 1 and resource 2. We then adjusted the supply landscape for resource 2 to have a fixed correlation ρ with the supply landscape for resource 1. The spatial correlations were $\rho = -1$ representing fully anticorrelated resource supplies, $\rho = 0$ representing uncorrelated resource supplies, and $\rho = 0.95$ representing highly correlated resource supplies. The details of the supply landscape generation procedure are described in in Appendix S2.2.2.

In total, we thus generated 9 combinations of resource supply landscapes $K_1(\mathbf{x})$ and $K_2(\mathbf{x})$. For each of these 9 supply-landscape combinations we numerically solved for the evolutionarily stable community of coexisting consumers (as described in Appendix S2.2.2), and did so across the same range of combinations of resource types (substitutable to essential) and trade-off curves (generalist- to specialist-favoring) as used in the example presented in the main text (Fig. 6). In the following, we present the results for the remaining eight supply landscape combinations in figures showing the numbers of evolutionarily stably coexisting consumers as functions of the curvatures of the generalist ZNGIs and trade-off curves. While the different supply landscapes generate significant quantitative variation in the outcomes, the qualitative patterns are all very similar to Fig. 6A in the main text, and are consistent with what we describe and analyze there.

Note that landscapes in which the local supplies of resources 1 and 2 are fully correlated ($\rho = 1$) would yield the same outcome as the homogeneous case (Fig. 4 in main text), because the local supply ratios $K_1(\mathbf{x})/K_2(\mathbf{x})$ would be equal to 1 everywhere in space. The maximum numbers of coexisting consumers are therefore lowest in the numerical examples of highly correlated resource supplies ($\rho = 0.95$).

S3.3.1 Supply-landscape pair 1, $\rho = -1$

This is the set of resource supplies that was used in the main text to illustrate evolutionary outcomes in spatially heterogeneous landscapes. The resource 1 and resource 2 supply landscapes and the resulting evolutionary outcomes are shown in Figs. 2 and 6A in the main text. The remaining combinations of resource 1 and resource 2 supply landscapes and the resulting evolutionary outcomes are shown in the remainder of this appendix.

S3.3.2 Supply-landscape pair 1, $\rho = 0$

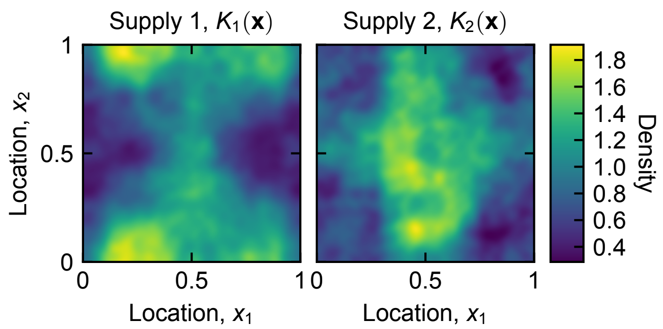

Figure S3.10: Resource supply for resource 1, $K_1(\mathbf{x})$, and resource supply for resource 2, $K_2(\mathbf{x})$, for supply-landscape pair 1. The Pearson correlation between $K_1(\mathbf{x})$ and $K_2(\mathbf{x})$ is 0. Both panels share the same color scale.

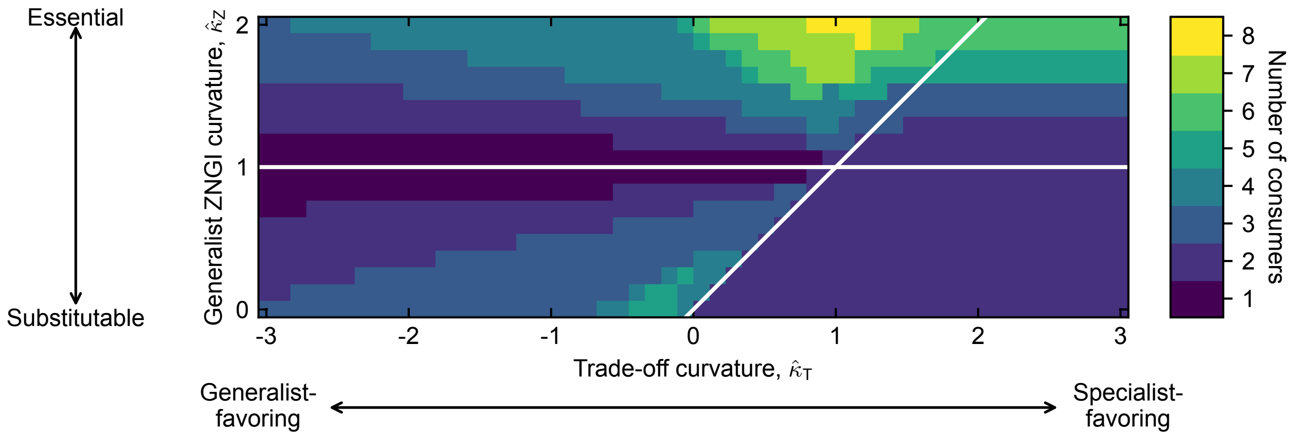

Figure S3.11: Number of consumers that coexist evolutionarily stably for different trade-off and generalist zero net growth isocline (ZNGI) curvatures, for the resource supplies depicted in Fig. S3.10.

S3.3.3 Supply-landscape pair 1, $\rho = 0.95$

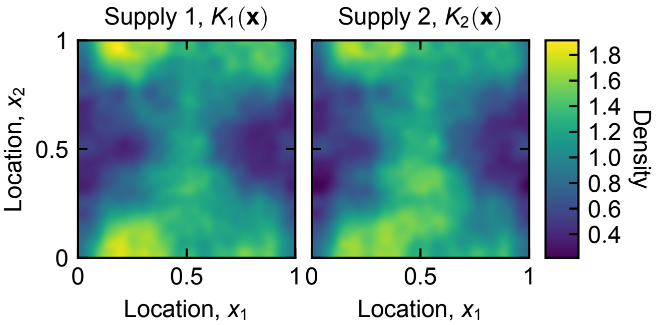

Figure S3.12: Resource supply densities for resource 1, $K_1(\mathbf{x})$, and resource 2, $K_2(\mathbf{x})$, for supply-landscape pair 1. The Pearson correlation between $K_1(\mathbf{x})$ and $K_2(\mathbf{x})$ is 0.95. Both panels share the same color scale.

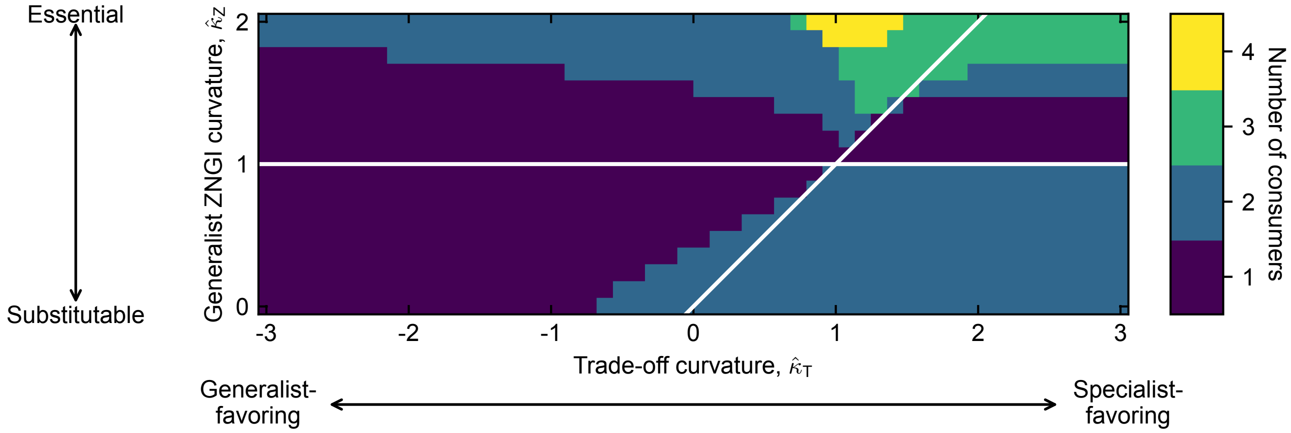

Figure S3.13: Number of consumers that coexist evolutionarily stably for different trade-off and generalist zero net growth isocline (ZNGI) curvatures, for the resource supplies depicted in Fig. S3.12.

S3.3.4 Supply-landscape pair 2, $\rho = -1$

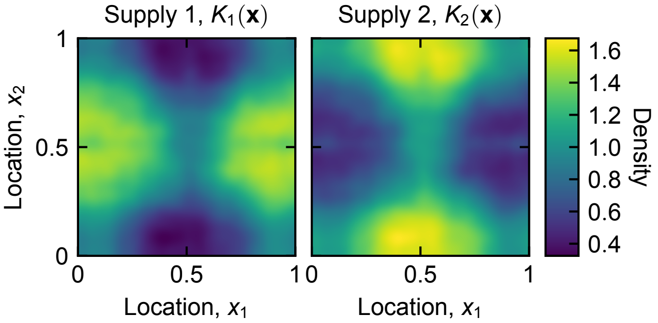

Figure S3.14: Resource supply densities for resource 1, $K_1(\mathbf{x})$, and resource 2, $K_2(\mathbf{x})$, for supply-landscape pair 2. The Pearson correlation between $K_1(\mathbf{x})$ and $K_2(\mathbf{x})$ is -1. Both panels share the same color scale.

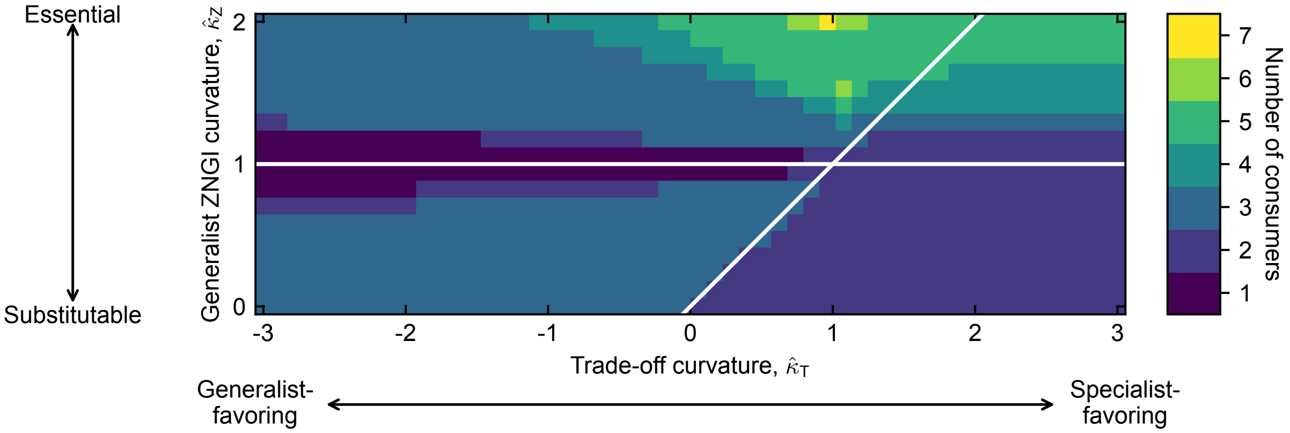

Figure S3.15: Number of consumers that coexist evolutionarily stably for different trade-off and generalist zero net growth isocline (ZNGI) curvatures, for the resource supplies depicted in Fig. S3.14.

S3.3.5 Supply-landscape pair 2, $\rho = 0$

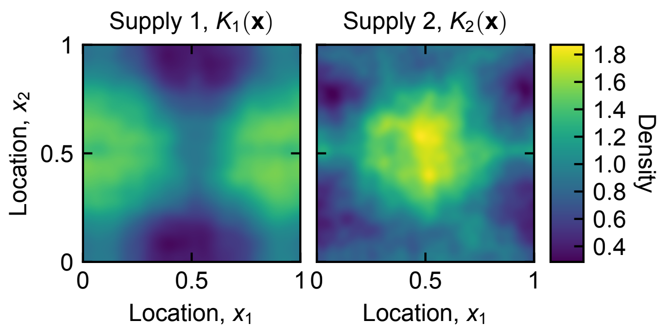

Figure S3.16: Resource supply densities for resource 1, $K_1(\mathbf{x})$, and resource 2, $K_2(\mathbf{x})$, for supply-landscape pair 2. The Pearson correlation between $K_1(\mathbf{x})$ and $K_2(\mathbf{x})$ is 0. Both panels share the same color scale.

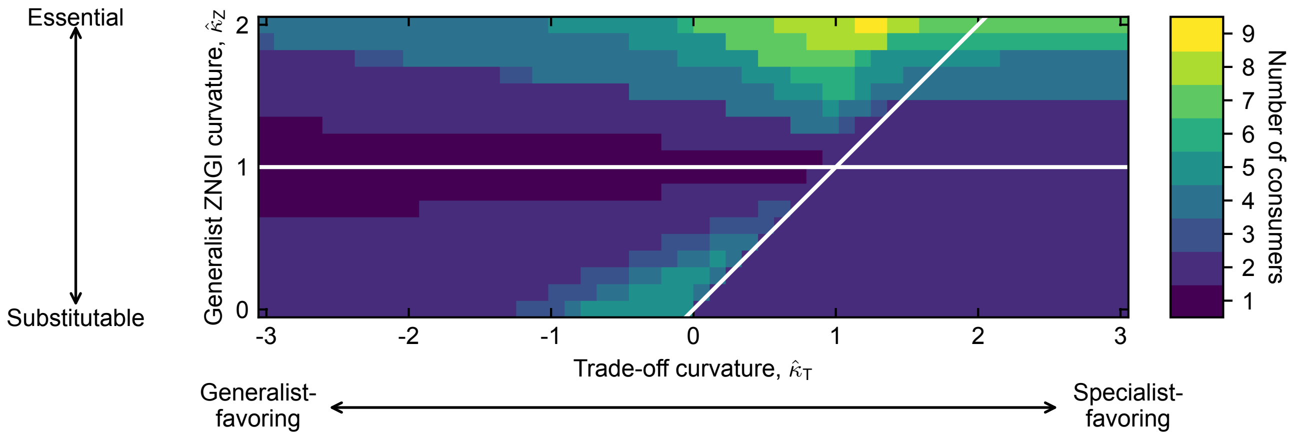

Figure S3.17: Number of consumers that coexist evolutionarily stably for different trade-off and generalist zero net growth isocline (ZNGI) curvatures, for the resource supplies depicted in Fig. S3.16.

S3.3.6 Supply-landscape pair 2, $\rho = 0.95$

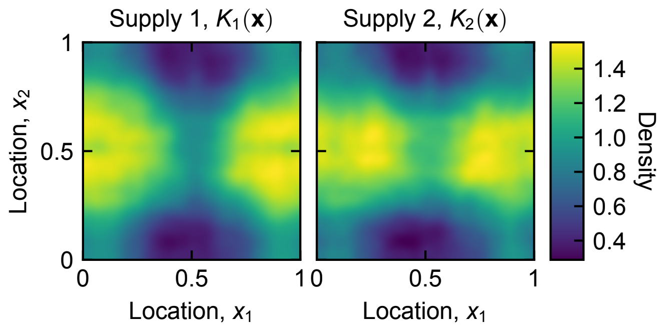

Figure S3.18: Resource supply densities for resource 1, $K_1(\mathbf{x})$, and resource 2, $K_2(\mathbf{x})$, for supply-landscape pair 2. The Pearson correlation between $K_1(\mathbf{x})$ and $K_2(\mathbf{x})$ is 0.95. Both panels share the same color scale.

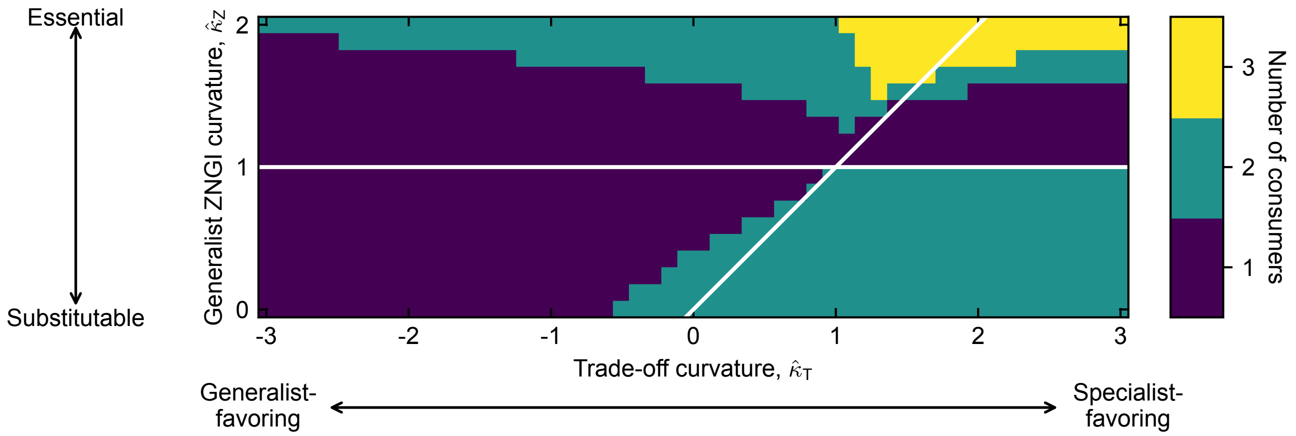

Figure S3.19: Number of consumers that coexist evolutionarily stably for different trade-off and generalist zero net growth isocline (ZNGI) curvatures, for the resource supplies depicted in Fig. S3.18.

S3.3.7 Supply-landscape pair 3, $\rho = -1$

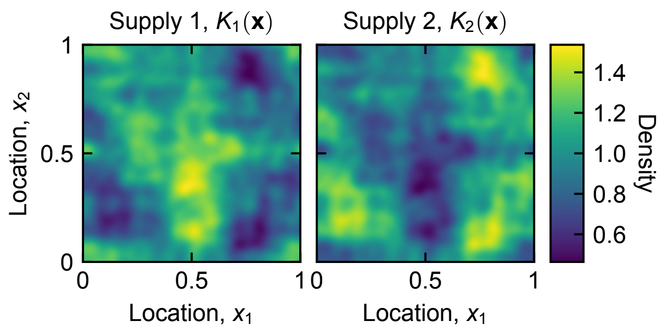

Figure S3.20: Resource supply densities for resource 1, $K_1(\mathbf{x})$, and resource 2, $K_2(\mathbf{x})$, for supply-landscape pair 3. The Pearson correlation between $K_1(\mathbf{x})$ and $K_2(\mathbf{x})$ is -1. Both panels share the same color scale.

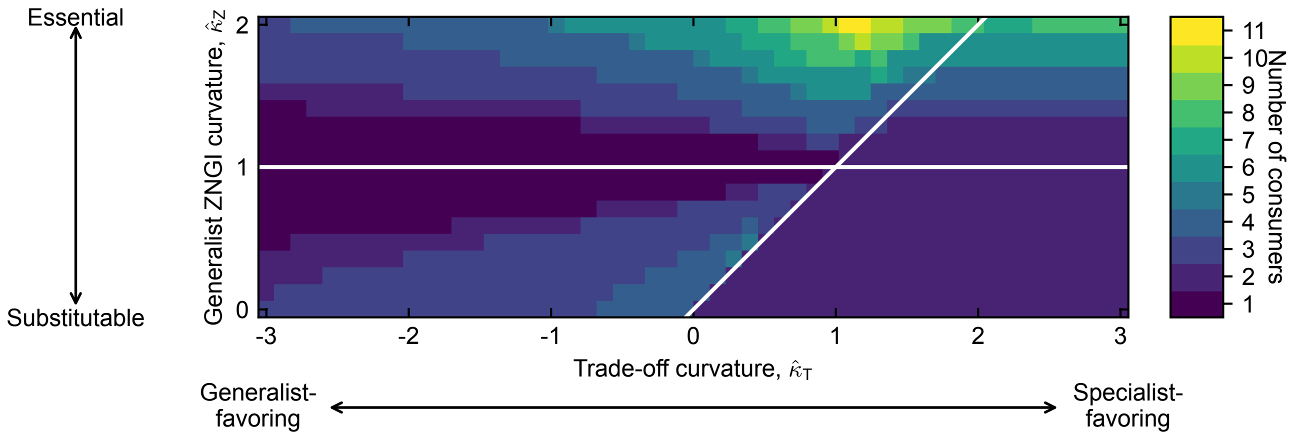

Figure S3.21: Number of consumers that coexist evolutionarily stably for different trade-off and generalist zero net growth isocline (ZNGI) curvatures, for the resource supplies depicted in Fig. S3.20.

S3.3.8 Supply-landscape pair 3, $\rho = 0$

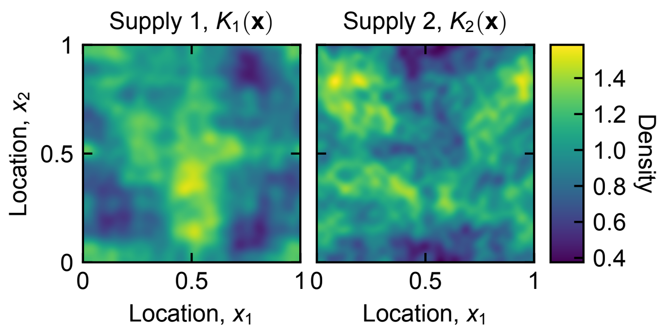

Figure S3.22: Resource supply densities for resource 1, $K_1(\mathbf{x})$, and resource 2, $K_2(\mathbf{x})$, for supply-landscape pair 3. The Pearson correlation between $K_1(\mathbf{x})$ and $K_2(\mathbf{x})$ is 0. Both panels share the same color scale.

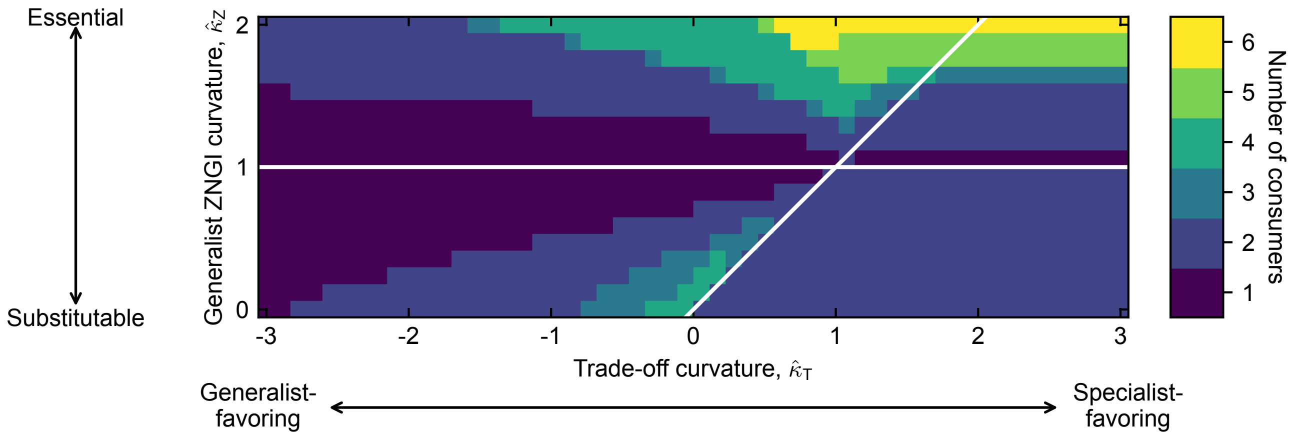

Figure S3.23: Number of consumers that coexist evolutionarily stably for different trade-off and generalist zero net growth isocline (ZNGI) curvatures, for the resource supplies depicted in Fig. S3.22.

S3.3.9 Supply-landscape pair 3, $\rho = 0.95$

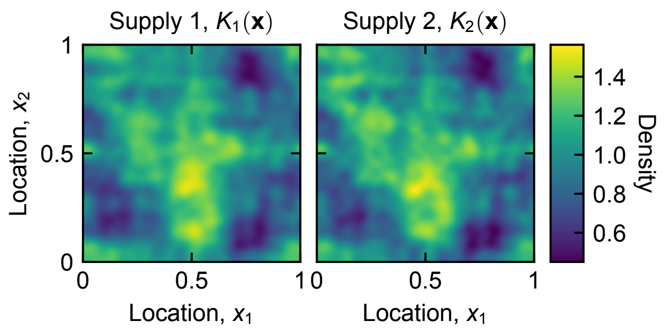

Figure S3.24: Resource supply densities for resource 1, $K_1(\mathbf{x})$, and resource 2, $K_2(\mathbf{x})$, for supply-landscape pair 3. The Pearson correlation between $K_1(\mathbf{x})$ and $K_2(\mathbf{x})$ is 0.95. Both panels share the same color scale.

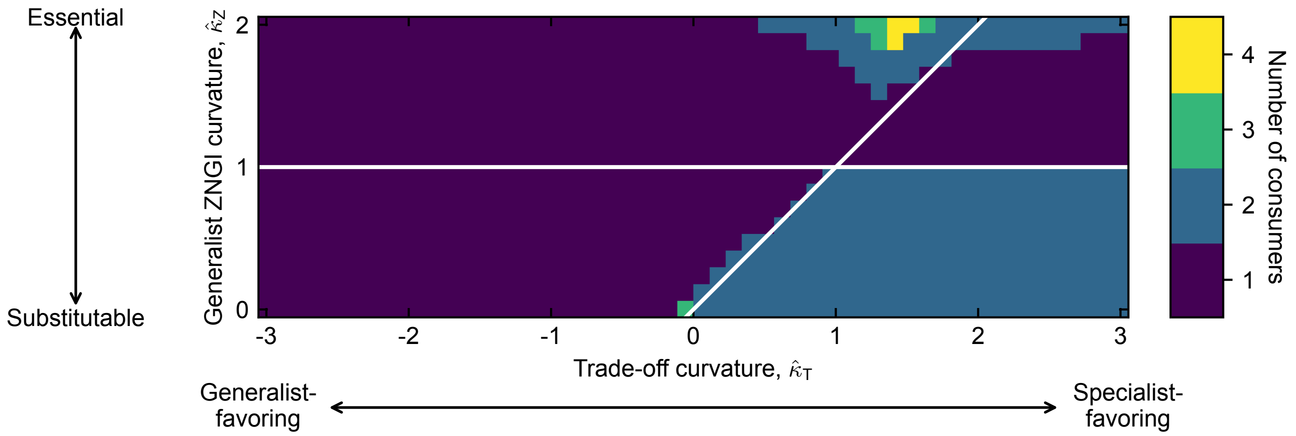

Figure S3.25: Number of consumers that coexist evolutionarily stably for different trade-off and generalist zero net growth isocline (ZNGI) curvatures, for the resource supplies depicted in Fig. S3.24.
